# CDK12/13 inhibition overcomes platinum resistance by impairing RNA metabolism

**DOI:** 10.1038/s41392-026-02932-w

**Published:** 2026-09-03

**Authors:** Ilenia Pellarin, Valentina Rossi, Riccardo Giuseppe Margiotta, Javad Karimbayli, Alessio Polacchini, Ashwani Bahl, Milena Nicoloso, Maura Sonego, Sara D’Andrea, Laura Sala, Simone Canesi, Andrea Favero, Anna Nespolo, Danilo Licastro, Michele Bartoletti, Fabio Puglisi, Antonino Ditto, Vincenzo Canzonieri, Monica Schiappacassi, Iain McNeish, Guido Sanguinetti, Barbara Belletti, Gustavo Baldassarre

**Affiliations:** 1 Molecular Oncology Unit, Centro di Riferimento Oncologico di Aviano (CRO) IRCCS, National Cancer Institute, Aviano (PN), Italy; 2 University of Trieste, Trieste (TS), Italy; 3 Theoretical and Scientific Data Science, Scuola Internazionale Superiore di Studi Avanzati, Trieste, Italy; 4 Carrick Therapeutics, Blanchardstown Corporate Park, Dublin, Ireland; 5 Mouse and Animal Pathology Laboratory, UNIMI Foundation, Milan, Italy; 6 Department of Veterinary Medicine and Animal Sciences, University of Milan, Lodi, Italy; 7 AREA Science Park, Padriciano, Trieste, Italy; 8 Department of Medical Oncology, Centro di Riferimento Oncologico di Aviano (CRO) IRCCS, National Cancer Institute, Aviano (PN), Italy; 9 Department of Medicine, University of Udine, Udine (UD), Italy; 10 Gynecological Surgery Unit, Centro di Riferimento Oncologico di Aviano (CRO) IRCCS, National Cancer Institute, Aviano (PN), Italy; 11 Pathology Unit, Centro di Riferimento Oncologico di Aviano (CRO) IRCCS, National Cancer Institute, Aviano (PN), Italy; 12 Department of Medical, Surgical and Health Sciences, University of Trieste, Trieste (TS), Italy; 13 Ovarian Cancer Action Research Centre, Department of Surgery and Cancer, Imperial College, London, UK

**Keywords:** Gynaecological cancer, Cancer therapy, Gene expression analysis

## Abstract

Epithelial ovarian cancer (EOC) is a highly lethal disease characterized by a high rate of platinum (PT)-resistant recurrence. Dysregulation of transcriptional and alternative splicing (AS) has been suggested as a possible mechanism of PT resistance involving transcriptional cyclin-dependent kinases (CDKs). We report that pharmacological CDK12/CDK13 inhibition (CDK12i) synergistically enhances PT-induced cytotoxicity in a large number of PT-sensitive and PT-resistant EOC models. CDK12i effectively delayed the emergence of PT-resistant recurrences in vitro and in vivo. Acquired resistance to CDK12i enhanced PT sensitivity, revealing a therapeutically exploitable vulnerability to PT. CDK12i strongly reshapes transcriptional elongation and RNA processing, impacting multiple categories of AS, with marked enrichment of transcripts involved in the DNA damage repair (DDR) pathway. We proved that CDK12 interacts with the splicing master regulator SFPQ/p54^nrb^ complex, driving its distribution in transcriptional condensates. The p54^nrb^ subunit of the complex is required for the CDK12-SFPQ interaction and to ensure splicing reprogramming and CDK12i-induced cytotoxicity in all the EOC models tested. The use of CT7439, a novel clinical-grade CDK12i, which is now in phase I/II clinical trials, confirmed the therapeutic potential of CDK12i, either as monotherapy or in combination with PT. These findings emphasize that the therapeutic potential of CDK12i extends beyond DDR regulation, encompassing a critical role in RNA processing and AS. This dual impact affects EOC survival and may provide a novel, clinically relevant therapeutic strategy for hard-to-treat PT-resistant patients for whom effective treatment options remain limited.

## Introduction

Epithelial ovarian cancer (EOC) is a relatively uncommon but highly lethal malignancy and remains the fifth leading cause of cancer-related death among women in developed countries.^1^ Mortality is largely driven by the absence of symptoms at early stages, which results in late clinical diagnosis, widespread peritoneal dissemination, and the rapid emergence of chemoresistance.^2^ Although EOC encompasses multiple molecular subtypes, including high- and low-grade serous, endometrioid, and clear cell carcinomas, the standard of care remains quite invariably represented by cytoreductive surgery followed by platinum (PT) and taxane-based chemotherapy, with subsequent maintenance therapy using bevacizumab (BEV) and/or PARP inhibitors (PARPi).^1–4^ Despite high initial response rates, approximately 75% of patients with advanced disease ultimately relapse and develop PT-resistant recurrences, often accompanied by acquired cross-resistance to PARPis,^1,5^ underscoring the urgent need for novel therapeutic strategies to overcome PT resistance.

Emerging evidence suggests that PT resistance is not driven primarily by the progressive accumulation of genetic mutations, but rather by the establishment of a transcriptionally dysregulated state, supported by both genetic and nonmutational epigenetic mechanisms.^6,7^ This concept does not imply that genetic alterations are irrelevant. Instead, genetic lesions and epigenetic regulation cooperate to shape resistance. Mutations in genes involved in homologous recombination, chromatin remodeling, or signaling pathways may predispose cells to adaptive responses, but, in this setting, the maintenance of the resistant phenotype often depends on sustained transcriptional programs rather than continued genetic evolution. This phenomenon, known as “transcriptional addiction”, refers to the dependence of cancer cells on continuous activity of specific transcriptional programs for survival. Similar to oncogene addiction, where tumors rely on a particular signaling pathway, transcriptionally addicted cells become highly dependent on the machinery that maintains their aberrant gene expression networks. In this scenario, transcriptional cyclin-dependent kinases (CDKs) are placed at the core of adaptive survival programs in therapy-challenged cancer cells.^8^ Among these, cyclin-dependent kinase 12 (CDK12) has attracted particular attention because of its central role in RNA polymerase II (Pol II) transcriptional elongation, DNA damage response (DDR) gene expression, and RNA processing.^9–13^ CDK12 forms an active complex with Cyclin-K that phosphorylates Pol II at Ser2, thereby sustaining the efficient transcription of long, structurally complex DDR genes.^14–16^ Loss or pharmacological inhibition of CDK12 disrupts Pol II processivity, promotes premature transcriptional termination, and induces a “BRCAness” phenotype without global suppression of transcription.^11,17–19^ In contrast, CDK13 inhibition predominantly affects pathways related to cellular growth and metabolism, with only a minimal effect on DDR gene expression.^18^ In this context, CDK12 is not merely regulator of gene expression, but it represents a molecular hub that sustain the plasticity and survival of therapy-resistant cancer cells. Targeting this hub may thus offer the possibility of disrupting the transcriptional architecture that underlies platinum resistance.

Genomic alterations affecting CDK12 have been detected in high-grade serous ovarian cancer (HGSOC), as well as in prostate and breast cancers, where they contribute to genomic instability and create therapeutically exploitable vulnerabilities.^11,20^ Pharmacological inhibition of CDK12/13 (CDK12i) sensitizes cancer cells to DNA-damaging agents and, possibly, PARPi while simultaneously exposing a broad landscape of actionable dependencies, thereby supporting CDK12/13 as emerging therapeutic targets.^21^ Recent drug development efforts have led to the generation of multiple dual CDK12/13 inhibitors that have shown therapeutic potential in triple negative breast cancer models by causing a BRCAness phenotype.^22–25^ Among these inhibitors, the orally bioavailable selective CDK12/13 inhibitor CT7439^26^ is the first clinical-stage compound currently being evaluated in a phase I/II trial (NCT06600789).

Despite the extensive characterization of transcriptional outputs following CDK12/13 inhibition, the molecular mechanisms coupling CDK12 activity to cotranscriptional RNA-processing complexes remain largely unresolved. Emerging evidence indicates that perturbation of the transcription cycle rapidly reshapes the localization and functional landscape of RNA-binding proteins (RBPs), revealing a tight dependence of RBP networks on active transcription.^27,28^ In this context, the SFPQ and p54^nrb^ proteins, members of the Drosophila behavior/human splicing (DBHS) family, have emerged as particularly compelling candidates as multifunctional nuclear regulators involved in both splicing and transcriptional control.^29^ SFPQ/p54^nrb^ associates with promoter-proximal chromatin and Pol II to support transcriptional elongation, pre-mRNA 3′ end processing, and the efficient expression of long genes.^30–34^ However, the mechanism through which SFPQ/p54^nrb^ is integrated with CDK12 activity is largely unexplored.

Here, we identify a functional axis between CDK12 and the SFPQ/p54^nrb^ complex that is essential for sustaining transcriptional elongation and splicing, thus regulating the expression of key DDR genes, including ATM and RAD51. We further show that CDK12/13 inhibition disrupts this axis, revealing an actionable vulnerability in EOC. From a therapeutic perspective, we demonstrate that CDK12i markedly enhances PT-induced cytotoxicity in both PT-sensitive and PT-resistant models and significantly delays or even prevents the emergence of PT-resistant recurrences in vitro and in vivo. Importantly, these effects extend to CT7439, a CDK12/13 inhibitor already in clinical development, underscoring the strong translational relevance of targeting the CDK12-SFPQ/p54^nrb^ axis, particularly in PT-resistant EOC, for which effective therapeutic options remain extremely limited.

## Results

**Fig. 1:**
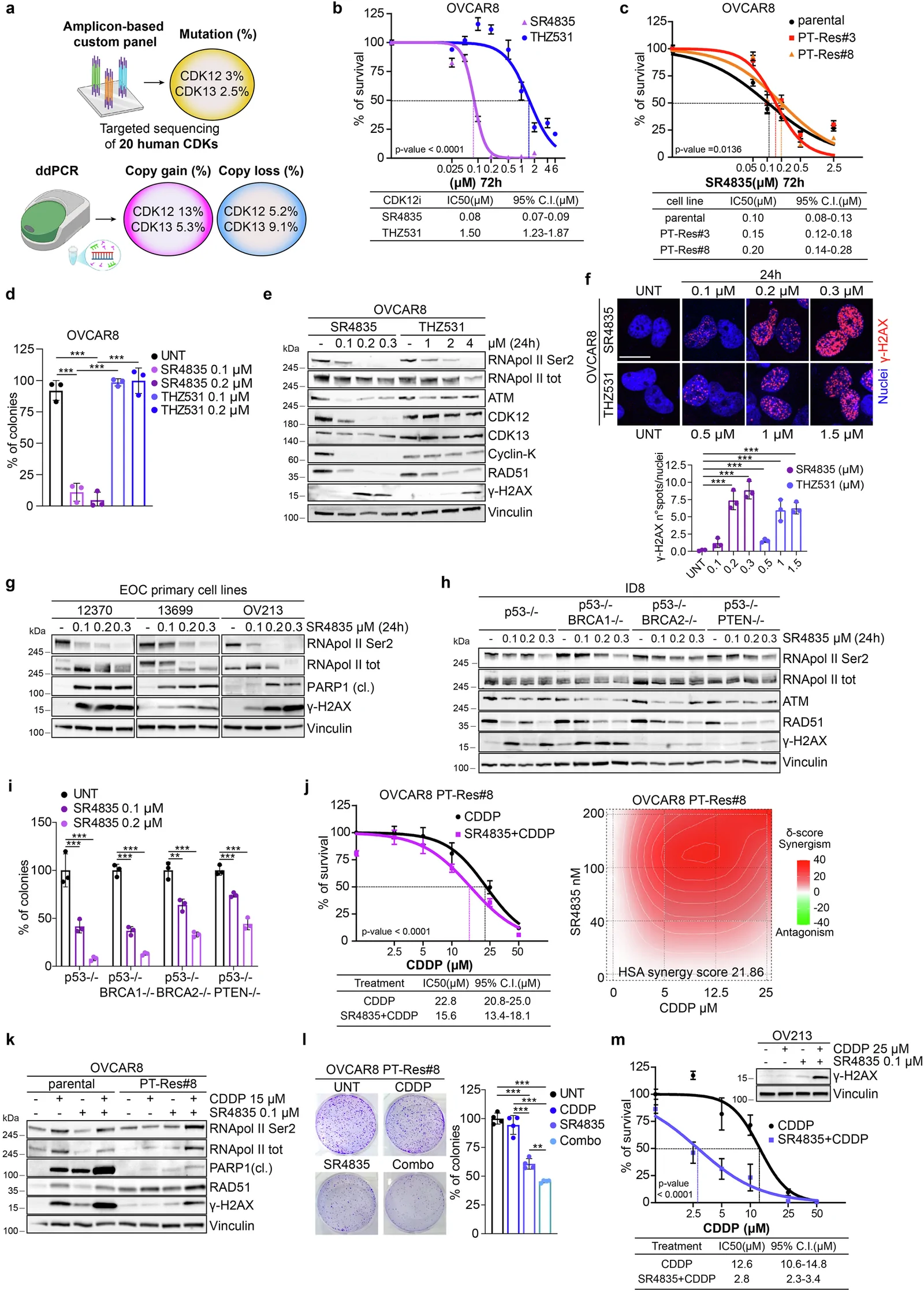
CDK12i impairs EOC cell viability, affecting the DDR and potentiating the efficacy of cisplatin (CDDP) in PT-Res models. **a** Schematic representation of next-generation sequencing (NGS) and droplet digital PCR (ddPCR) approaches used to profile all CDK mutations and copy number alterations (CNAs) in 196 samples from 186 patients. The percentages indicate the alteration frequencies of CDK12 and CDK13. **b**, **c** Nonlinear regression fitting of cell viability. In (**b**), OVCAR8 cells were treated for 72 h with increasing doses of CDK12/13 inhibitors (CDK12i, THZ531 and SR4835), and in (**c**), OVCAR8 parental and isogenic PT-Res clones (#3 and #8) were treated with increasing doses of SR4835 for 72 h. The data represent the percentage mean (±SD; *n* = 3 biological replicates) of viable cells relative to that of untreated cells. The IC50 values and confidence intervals (CIs) are reported in the tables. **d** Quantification of the clonogenic activity of OVCAR8 cells treated with THZ531 or SR4835 (100 and 200 nM) for 72 h. The graphs show the percentage of counterstained colonies normalized to the untreated condition, with *n* = 3 independent experiments. **e** WB was used to evaluate the expression of the indicated proteins in OVCAR8 cells treated with SR4835 or THZ531 for 24 h. Vinculin was used as a loading control. **f** Immunofluorescence analysis (IF) and quantification of γ-H2AX foci in OVCAR8 cells treated with SR4835 or THZ531 for 24 h. Scale bars, 20 μm. The data represent the mean number of γ-H2AX puncta/nuclei (±SD; *n* = 3 biological replicates). **g** Western blot (WB) analysis of 12370, 13699, and OV213 cells treated with SR4835 for 24 h to evaluate the expression of RNA Pol II Ser2 phosphorylation and the expression of the markers of DNA damage (γ-H2AX) and apoptosis, such as cleaved PARP1 (reported as PARP1 cl.). Vinculin served as a loading control. **h** WB was used to evaluate the expression of the indicated proteins in ID8 murine cells with different molecular backgrounds treated with SR4835 for 24 h. Vinculin was used as a loading control. **i** Quantification of the clonogenic activity of ID8 murine cells treated with increasing doses of SR4835 for 72 h (*n* = 3 biological replicates). **j** Nonlinear regression analysis of cell viability in OVCAR8 PT-Res#8 cells pretreated with ±SR4835 (100 nM) for 16 h and then treated with increasing doses of cisplatin (CDDP) for 48 h. The data represent the percentage mean (±SD; *n* = 3 biological replicates) of viable cells relative to that of untreated cells. The IC50 values and confidence intervals (CIs) are reported in the table. On the right, two-dimensional drug interaction maps showing synergistic and antagonistic dose combinations in red and green, respectively. The darkest red area identifies the region of maximal synergy. Highest single agent (HSA) scores are indicated; values > 10 denote synergy, whereas values <−10 denote antagonism. **k** WB was used to evaluate the expression of the indicated proteins in OVCAR8 parental and PT‑Res#8 cells pretreated with ± SR4835 (100 nM) for 16 h and then exposed to CDDP (15 μM) for 48 h. Vinculin was used as a loading control. **l** Representative images and quantification of the results of the clonogenic assay in OVCAR8 PT-Res#8 cells pretreated with ± SR4835 (100 nM) for 16 h and then exposed to CDDP (15 μM) for 48 h. The data represent the mean (±SD) of *n* = 3 biological replicates. **m** Nonlinear regression fitting of the viability of OV213 PT‑Res primary cells pretreated with ± SR4835 (100 nM) for 16 h and then exposed to increasing doses of CDDP for 48 h. The data represent the percentage mean (±SD; *n* = 3 biological replicates) of viable cells relative to that of untreated cells. The IC50 values and confidence intervals (CIs) are reported in the table. In the inset, the WB image shows the expression of γ-H2AX in cells treated as described above. Vinculin was used as a loading control. In the figure, significance was calculated using Fisher’s exact test (**b**, **c**, **j** and **m**) or ordinary one-way ANOVA (**d**, **f**, **i** and **l**) and is expressed as follows: *p < 0.05, **p < 0.01, and ***p < 0.001

Dysregulation of CDK activity in ovarian cancer could represent a potential therapeutic target.^17,35^ However, to date, a specific analysis of the mutational status of all CDKs in EOC has never been reported. To evaluate the possible implications of CDK alterations in EOC, we designed an amplicon-based custom NGS panel to unbiasedly sequence the 20 human CDKs in a cohort of 196 EOC samples collected at our institute from 186 patients and 29 ovarian cancer-derived cell lines. All samples were sequenced at high coverage (i.e., median above 500X) to detect subclonal alterations (see Supplementary Table 1 and “Methods” for details). In agreement with previous data,^9,12^ CDK12 and CDK13 were the most frequently mutated CDKs (3% and 2,5% of the cases, respectively) in EOC. CDK13 mutations and variants of uncertain significance (VUS), which are predicted to be likely pathogenic, cluster within the kinase domain. In contrast, CDK12 mutations are more dispersed within different protein domains and are all annotated in OncoKB (Supplementary Fig. 1a–b). No mutation was detected in the cell lines, except for COV362, which was mutated for CDK17. Focusing on CDK12/13, we then evaluated their copy number alteration (CNA) using a droplet digital PCR approach. CDK12 was amplified in 13% of EOC samples, while CDK13 was amplified in 5.3%. Conversely, copy number loss was more frequent for CDK13 (9.1%) than for CDK12 (5.2%) (see Supplementary Table 1 for details) (Fig. 1a). The analysis of ascites samples derived from the same patients in the cohort but collected at different stages of disease progression (*n* = 14 patients, for a total of 32 matched samples) revealed the absence of CNA in either CDK12 or CDK13, except for one patient who acquired CDK13 amplification at relapse.

We then evaluated the correlation of CDK12 and CDK13 genomic alterations with clinical variables and response to therapies (Supplementary Table 2). Patients with CDK12 gain-of-function (GoF) alterations had a median progression-free survival (PFS) of 16 months and overall survival (OS) of 38 months, compared to patients with wild-type CDK12 (WT = PFS 21 months, OS 48 months) and loss-of-function alterations (LoF = PFS 24, OS 52 months) (Supplementary Fig. 1c–d). Patients with CDK13 GoF alterations had a median OS of 40 months with respect to their WT (51 months) and LoF counterparts (68.5 months), with no difference in PFS (Supplementary Fig. 1e–f). However, these differences did not reach statistical significance in our EOC cohort, possibly because of the low number of available cases. To evaluate a larger cohort, we interrogated publicly available databases, which revealed that high CDK12 and CDK13 mRNA, but not protein expression correlated with shorter patient overall survival, although the hazard ratio was marginally modified (Supplementary Fig. 1g–l). Overall, these data suggest that CDK12 and CDK13 expression has limited prognostic significance in EOC. However, high CDK12 but not CDK13 expression significantly predicted a poor response to platinum (PT) in EOC patients, as assessed by receiver operating characteristic (ROC) curve analyses (Supplementary Fig. 1m–n).

### CDK12/CDK13 inhibition modulates the DDR and is cytotoxic to EOC cells

We next evaluated whether CDK12 and CDK13 could be functionally involved in EOC pathogenesis. To date, some studies have tested this hypothesis,^36,37^ focusing on a single inhibitor and/or on a few selected models, which leaves uncertainty as to whether CDK12/13 inhibition could be clinically useful, especially in the context of platinum-resistant EOC.^10,20,36–38^ To broaden the value of these observations, we used two distinct selective CDK12/13 inhibitors, THZ531 and SR4835 (hereafter collectively referred to as CDK12i), which differ in chemical structure, mechanism of action and efficacy.^23,24^ Using a large panel of EOC cell lines, we confirmed that CDK12i impaired EOC cell viability in a concentration-dependent manner, independent of histological subtype or *BRCA* mutation status (Supplementary Table 3, Fig. 1b and Supplementary Fig. 2a). Notably, CDK12i treatment significantly reduced the viability of both PT-sensitive and PT-resistant (PT-Res) isogenic cell lines established in our laboratory,^39–41^ although in some cases, compared with their PT-sensitive counterparts, PT-Res cells displayed a relative but incomplete reduction in sensitivity (Fig. 1c and Supplementary Fig. 2a).

Furthermore, CDK12i was particularly effective at killing HER2-amplified cell lines, including OV90 (EOC), SKBR3 and BT474 (breast cancer) (Supplementary Fig. 2a), likely because of the frequent coamplification of HER2 and CDK12. In total, using 39 different in vitro models, we established that both THZ531 and SR4835 were effective at killing cancer cells at nanomolar concentrations. In all the cases, the noncovalent inhibitor SR4835 was the most potent drug (Supplementary Fig. 2a).

Similar results were observed in long-term colony assays, where both THZ531 and SR4835 reduced colony formation, with the latter showing greater efficacy (Fig. 1d and Supplementary Fig. 2b). This cytotoxicity was accompanied by an increased DNA damage response (DDR), as assessed by γ−H2AX expression, in all the tested models (Fig. 1e–f and Supplementary Fig. 2c–e), suggesting that CDK12i could induce defects in the DNA repair pathway, as proposed by others.^18,24^ SR4835, a CDK12/13 inhibitor and Cyclin-K degrader, displayed greater potency than the covalent CDK12i THZ531 in the induction of DNA damage (Fig. 1e–f and Supplementary Fig. 2c–e).

CDK12i resulted in decreased phosphorylation of RNA polymerase II at Ser2, and for SR4835, CDK12i reduced cyclin-K expression, which is a well-established readout of activity for these inhibitors (Fig. 1e and Supplementary Fig. 2e). CDK12i leads to reduced expression of several genes involved in the DDR (Fig. 1e and Supplementary Fig. 2e), as previously reported.^19^ While the modulation of ATM and FANCD2 expression was partially cell line dependent, RAD51 expression was consistently affected across all the cellular models, with a dose-dependent decrease in both expression and foci formation following treatment with CDK12i (Fig. 1e and Supplementary Fig. 2c–e). This reduction was accompanied by the accumulation of DNA damage, as shown by increased γ-H2AX levels and PARP1 cleavage, ultimately leading to the upregulation of the expression of the apoptosis marker cleaved caspase 3 (Fig. 1e–f and Supplementary Fig. 2e). Notably, the phenotypic effects observed with pharmacological inhibitors were partially recapitulated by shRNA-mediated CDK12/CDK13 silencing in several cell lines (Supplementary Fig. 2f–h), suggesting that the effects of CDK12i were specific to the inhibition of these CDKs. Given that the potency of SR4835 was greater than that of THZ531 across all the experiments, with effective activity at nanomolar concentrations (Fig. 1b–f and Supplementary Fig. 2a–e), we selected it for all the subsequent experiments. CDK12i also demonstrated efficacy in genetically modified mouse ID8 EOC cells^42,43^ and in different primary EOC cultures^39,44^ obtained from patients who either responded (13699) or did not respond (12370) to platinum (PT)^45^ or who were derived from patient ascites and were collected at different stages of disease, including the PT-sensitive (OV206), PT-Res (OV213) and PARPi-resistant (PARPi-Res, OV225) stages (Fig. 1g and Supplementary Fig. 2i). Notably, CDK12/13 expression was retained during progression in this model (Supplementary Fig. 2j).

Our analyses of EOC and breast cancer cells did not reveal a clear association between CDK12i efficacy and the presence or absence of BRCA1/2 or homologous recombination (HR) defects. To better assess the impact of BRCA1/2 mutation status on the response to CDK12i, we used CRISPR-engineered mouse epithelial ovarian ID8 cells lacking TP53 and BRCA1, BRCA2, or PTEN.^42,43^ All ID8-modified cells were highly sensitive to SR4835, and BRCA1/2 alterations did not significantly modify CDK12i sensitivity, whereas PTEN deletion induced slightly increased resistance to the treatment, which was in line with their increased resistance to PT (Fig. 1h-i and Supplementary Fig. 3a-c).

Overall, these data showed that CDK12/13 expression is maintained during EOC progression and that CDK12i are active as a single agent in EOCs, regardless of their HR status, making these kinases attractive therapeutic targets for EOC patients.

### CDK12/CDK13 inhibition potentiates chemotherapy-induced EOC cell death

Our in vitro results revealed that CDK12i strongly affected DDR expression in both PT-sensitive and PT-Res EOC cell lines and that defects in the HR-DDR pathway are linked to an enhanced response to PT and/or PARPi.^46,47^ We thus evaluated the use of CDK12i as a strategy to restore PT and/or PARPi sensitivity in PT-Res EOC models. To test this hypothesis, we treated several PT-Res isogenic cellular models with SR4835 in combination with cisplatin (CDDP) or the PARPi niraparib. When administered in a sequential schedule, the nanomolar concentration of SR4835 effectively resensitized all the tested PT-Res cells to both CDDP and, partially, niraparib (Fig. 1j and Supplementary Fig. 4a–d). The strong synergistic effect of SR4835 and PT treatment on PT-Res EOC was confirmed by the corresponding highest single agent (HSA) score (Fig. 1j), which was further accompanied by increased expression of DNA damage (γ-H2AX) and apoptosis markers (cleaved PARP1), as determined by Western blot analyses (Fig. 1k), and by increased cytotoxicity, as determined by a colony formation assay (Fig. 1l). SR4835 synergized with niraparib in parental OVCAR8 cells, whereas in the PT-resistant counterpart, the effect was additive only (Supplementary Fig. 4e). Importantly, SR4835 improved the efficacy of CDDP and niraparib in primary cultures from a PT-resistant patient (OV213, Fig. 1m) that recurred after PARPi treatment (OV232, Supplementary Fig. 4f). Consistent with these findings, in genetically modified ID8 models, SR4835 improved the efficacy of CDDP and doxorubicin and partially improved the efficacy of niraparib (Supplementary Fig. 4g).

**Fig. 2:**
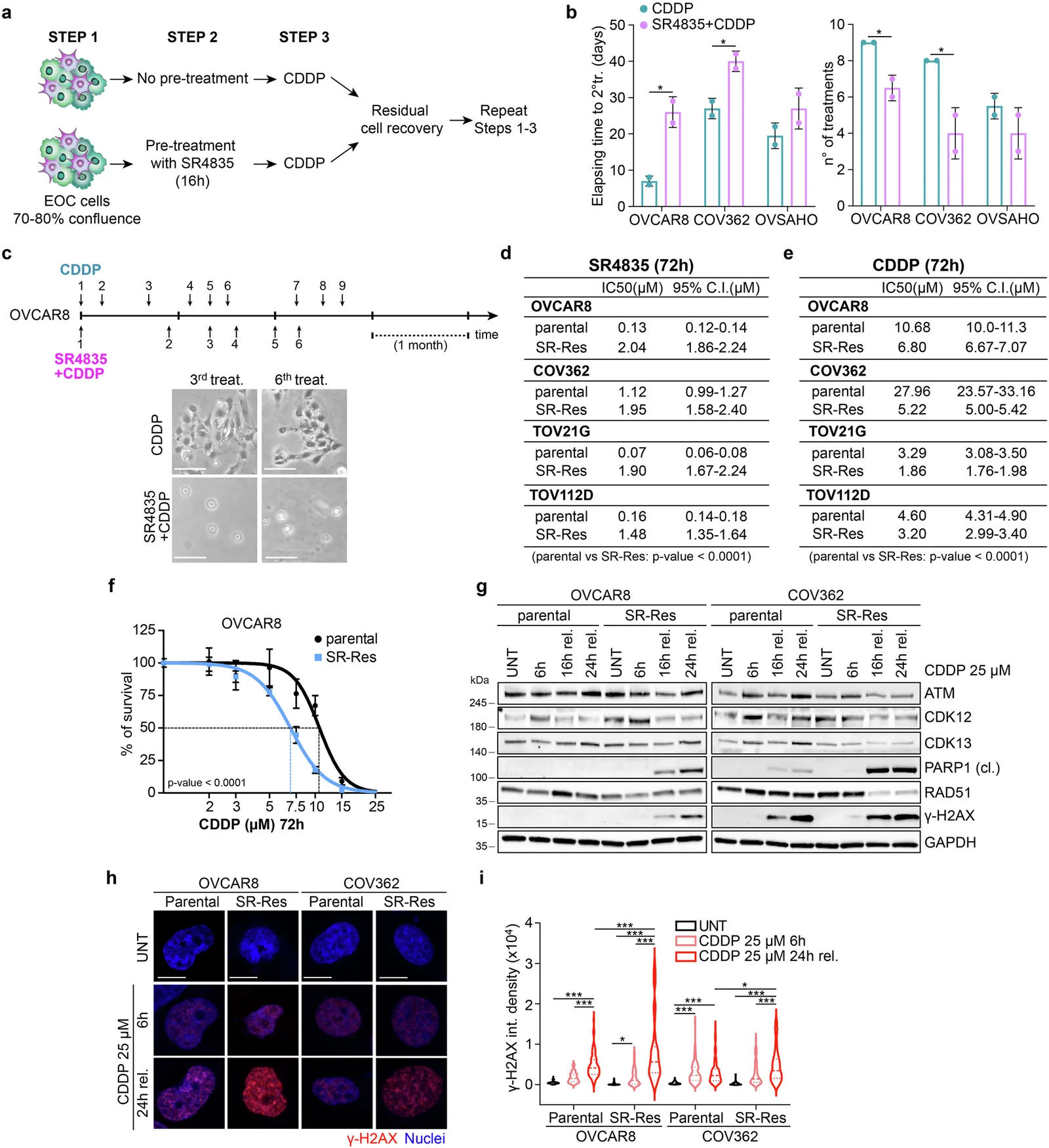
SR4835 resistance is associated with increased in vitro sensitivity to CDDP. **a** Scheme of the experiment used to evaluate the appearance of PT-Res clones in EOC cells treated with CDDP alone or in combination with SR4835. **b** Graphs showing the interval between the first and second treatment (left) and the number of CDDP ± SR4835 cycles (right) that could be performed in OVCAR8, COV362, and OVSAHO cells, using the schedule outlined in (**a**), (*n* = 2 biological replicates). Two-tailed, unpaired Student’s t tests were used. **c** Timeline of treatment cycles in OVCAR8 cells treated with CDDP alone or with SR4835 + CDDP, as detailed in (**a**). The lower panels show images of cells thawed after cryopreservation. Scale bar, 100 µm. **d**, **e** Table reporting the IC50 and CI values of nonlinear regression analysis of cell viability in OVCAR8, COV362, TOV21G, TOV112D parental and SR4835-Res (hereafter SR-Res) cells treated with SR4835 (**d**) or CDDP (**e**) for 72 h (*n* = 3 biological replicates). Fisher’s exact test corresponds to a *p* value < 0.0001 for all parental vs. SR-Res comparisons. **f** Nonlinear regression analysis of the cell viability of OVCAR8 parental and SR-Res cells treated for 72 h with increasing doses of CDDP. The data represent the mean (±SD; *n* = 3 biological replicates) number of viable cells relative to that of untreated cells. The IC50 values and confidence intervals (CIs) are reported in the table. **g** WB analysis evaluating the expression of the indicated proteins in OVCAR8 and COV362 parental and SR-Res cells treated with CDDP (25 μM) for 6 h and then released in CDDP-free medium for 16 ( + 16 h rel.) or 24 ( + 24 h rel.) h to allow DNA repair. GAPDH served as a loading control. Representative IF images (**h**) and a graph reporting the quantification of the integrated density of γH2AX (**i**) in OVCAR8 and COV362 cells treated as described in (**g**). Scale bars, 20 μm. In the figure, the statistical significance was assessed using Fisher’s exact test (**d**–**f**) or ordinary one-way ANOVA in (**i**) and is expressed as follows: *p < 0.05, **p < 0.01 and ***p < 0.001

These data indicate that CDK12i could resensitize PT-Res cells to chemotherapy and PARPi, but we also investigated whether CDK12i could prevent or delay the emergence of PT resistance. In accordance with the PT-Res isogenic cell line generation schedule,^41^ we sequentially pretreated or not PT-sensitive EOC cells with SR4835, followed by treatment with CDDP for different durations, and evaluated cell recovery after each treatment (Fig. 2a). In three EOC cell lines (OVCAR8, COV362, and OVSAHO), compared with CDDP alone, SR4835 pretreatment significantly prolonged the interval between the first and second treatment cycles (Fig. 2b, left panel), indicating a delayed onset of resistance. Furthermore, the combination was tolerated for significantly fewer treatment cycles than CDDP alone, which is consistent with impaired cell recovery after treatment and a progressive loss of viable cells (Fig. 2b, right panel). Notably, when the cultures were thawed after cryopreservation, the cells treated with only CDDP were able to attach and grow properly, whereas the cells treated with SR4835 + CDDP failed to recover over multiple attempts (Fig. 2c). Together, these findings demonstrate that CDK12i not only enhanced PT-induced cytotoxicity in both PT-sensitive and PT-Res models but also delayed and possibly reversed the development of PT resistance, preventing the long-term survival of treated cells and the emergence of PT-Res cell clones.

### SR4835-resistant cells are more sensitive to PT

To better understand whether the inability of EOC cells to recover from the combination treatment was due to effects related to prolonged CDK12i exposure or to a specific effect of the combination, we generated CDK12i-resistant models. Four different PT-sensitive EOC cell lines were treated for 72 h with sublethal nanomolar doses of CDK12i and allowed to recover. This procedure was repeated 20 times, using incremental doses of SR4835, until stably resistant clones, with up to a 20-fold higher SR4835 IC50 than the parental counterpart, were obtained (Fig. 2d and Supplementary Fig. 5a-c). SR4835-resistant cells (hereafter SR-Res) were also cross-resistant to THZ531 (Supplementary Fig. 2a). To the best of our knowledge, these represent the first EOC models of acquired resistance to CDK12i ever generated and compelling study models. Using comprehensive genomic profiling, complemented by our custom CDK-specific NGS panel, we observed that no mutation or CNA in any CDK gene—including CDK12/13—or in the 335 cancer-related genes covered by the panel was acquired during resistance acquisition (Supplementary Table 4). Importantly, these results indicated that the inability to recover from the combined treatment with CDDP and CDK12i was not due to prolonged treatment with CDK12i alone but instead to synergistic/additive effects derived specifically from the combined use of SR4835 and CDDP.

We next tested whether SR-Res cells acquired cross-resistance to other drugs commonly used to treat EOC patients, including PT, PARPi, doxorubicin and taxol. Surprisingly, SR-Res cells were particularly sensitive to PT (Fig. 2e–f and Supplementary Fig. 5d) and strongly resistant to taxol, while no appreciable difference was observed in the response to niraparib or doxorubicin (Supplementary Fig. 5e–f). Western blot analyses revealed that compared with control cells, SR-Res cells retained CDK12/13 expression and, upon CDDP exposure, exhibited reduced RAD51 expression and increased DNA damage accumulation, as indicated by increased γ-H2AX levels (Fig. 2g–i). Conversely, upon taxol treatment, the expression of acetylated tubulin strongly decreased in the SR-Res group (Supplementary Fig. 5g), which is widely used as a readout of effectively increased microtubule stabilization upon taxane treatment.^48,49^ This observation may indicate reduced microtubule stabilization after taxol treatment in SR-Res cells, possibly explaining their increased resistance to this drug.

Overall, our data suggested that CDK12i treatment sensitizes or resensitizes EOC cells to PT, preventing the emergence of PT-resistant clones.

### SR4835 cooperates with PT to block EOC tumor growth

**Fig. 3:**
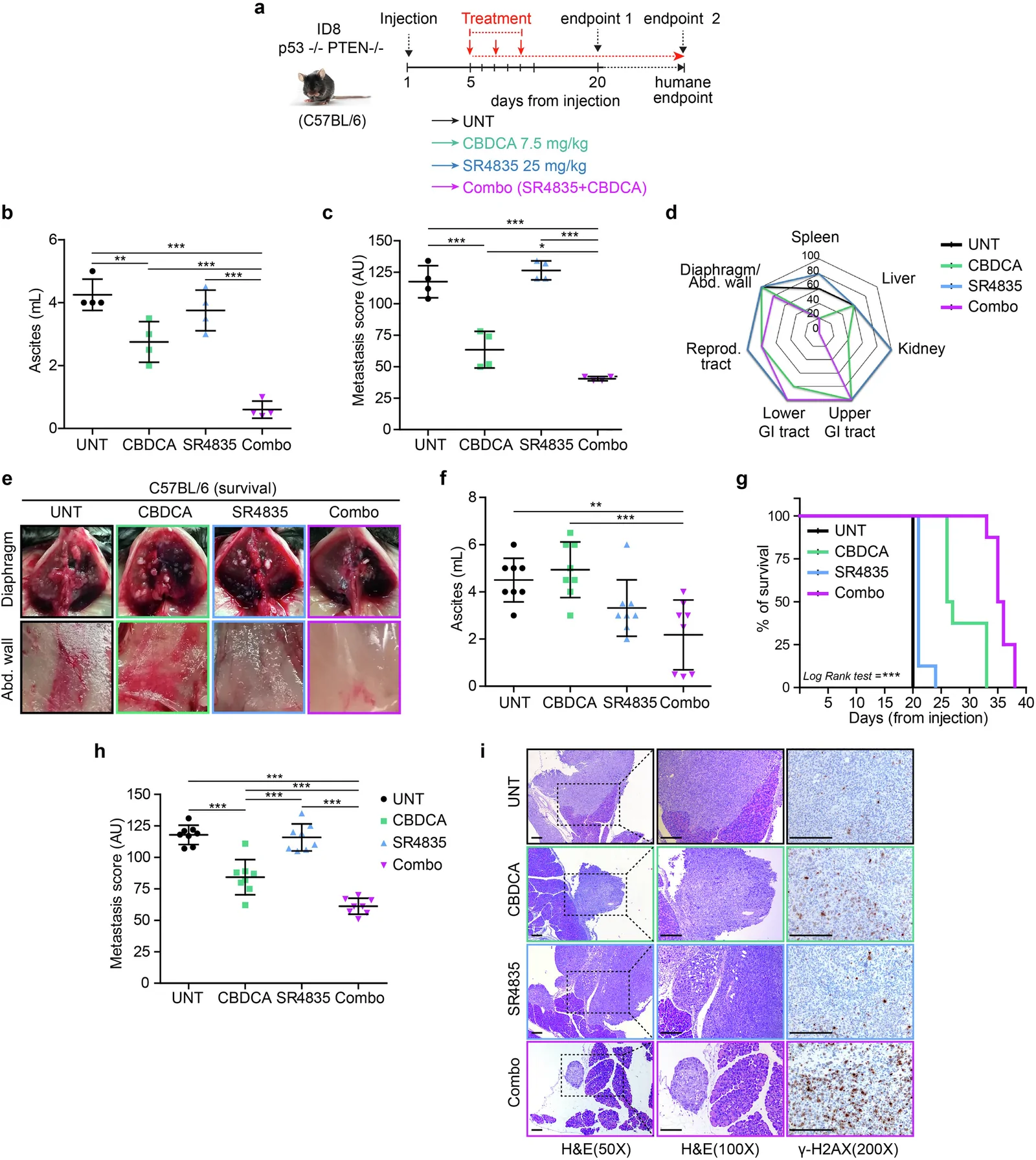
SR4835 cooperates with CBDCA to limit in vivo EOC tumor growth. **a**–**d** Graph showing the ascites volume (**b**), quantification (**c**) and distribution (radar plot) (**d**) of abdominal metastasis in C57BL/6 mice injected with ID8 p53^−/−^ PTEN^−/−^ syngeneic cells treated as in the scheme reported in (**a**) (endpoint 1) (*n* = 4 mice per condition). **e**–**h** Representative macroscopic images of the diaphragm and abdominal wall (**e**), ascites volume (**f**), Kaplan‒Meier survival curve (**g**) and quantification of abdominal metastasis (**h**) in C57BL/6 mice injected with ID8 p53^−/−^ PTEN^−/−^ cells and treated as in the scheme reported in (**a**), endpoint 2 (*n* = 8 mice per condition). **i** Hematoxylin and eosin (H&E) staining (at 50× and 100× magnification) for evaluating tumor masses and immunohistochemistry (IHC) for evaluating the expression of γ-H2AX (at 200× magnification) in mice treated as described in (**a**, endpoint 1). Scale bar is 400 μm. Significance was assessed with the log-rank (Mantel‒Cox) test (**g**) or one-way ANOVA (**b**, **c**, **f** and **h**). * *p* < 0.05, ** *p* < 0.01 and *** *p* < 0.001

To date, no data are available regarding CDK12i administration in EOC in vivo models; however, studies by Quereda et al. indicate that SR4835 is well tolerated, with no marked adverse events in mouse models of triple-negative breast cancer.^24^ We first evaluated the efficacy of SR4835 treatment in combination with PT by intraperitoneal (i.p.) injection of ID8 p53^−/−^/PTEN^−/−^ cells into syngeneic female C57BL/6 J mice. These cells constitute a highly aggressive model of EOC, with the highest resistance to CDDP^39,43^ and to SR4835, compared with all other ID8 genetically modified models (Supplementary Figs. 2a and 3a). The mice were randomized into four treatment groups and administered vehicle, SR4835, carboplatin (CBDCA), or a combination of SR4835 and CBDCA (Combo). The animals were sacrificed on day 20 postinjection (endpoint 1) for the assessment of intraperitoneal metastatic burden or were followed longitudinally for survival analysis (endpoint 2) (Fig. 3a). At 20 days, vehicle-treated mice produced more than 4 ml of hemorrhagic ascites and already formed multiple tumor deposits on the peritoneal wall and diaphragm surface, whereas the SR4835 + CBDCA group exhibited markedly reduced ascites volume, cellularity and tumor deposit formation compared with all the other treatment groups (Fig. 3b and Supplementary Fig. 6a–b). A thorough histopathological evaluation of metastatic dissemination further confirmed that, compared with the vehicle treatment alone, the combined treatment strongly decreased the tumor burden of p53^−/−^/PTEN^−/−^ ID8 cells in all organs in the abdominal cavity (Fig. 3c–d).

We then repeated the in vivo experiment in the survival setting, using a humane survival endpoint (Fig. 3a). Compared with mice in all the other groups, mice in the combination treatment group exhibited significantly prolonged survival. This survival benefit was associated with the lowest ascites volume and cellularity, as well as a marked reduction in metastatic dissemination, which was particularly evident in specific anatomical districts such as the reproductive tract and the abdominal wall (Fig. 3e–h and Supplementary Fig. 6c–f). Consistent with the in vitro results, ascites-derived lysates from mice treated with the combination treatment markedly increased γ-H2AX levels and reduced RAD51 expression, indicating impaired DDR (Supplementary Fig. 6g). Immunohistochemistry (IHC) and hematoxylin and eosin (H&E) staining of tumor explants and abdominal and pelvic organs confirmed elevated γ-H2AX expression and reduced tumor infiltration in SR4835 + CBDCA-treated mice, suggesting that this regimen could also impact tumor cell adhesion and/or motility (Fig. 3i). The same in vivo approach was carried out in immunodeficient female NU/NU CD1 athymic mice injected with ID8 p53^−/−^/PTEN^−/−^ cells. Overall, the observed outcomes were very consistent with those obtained in the C57BL/6 J immunocompetent model, although immunodeficient mice showed slightly reduced survival under both CBDCA- and combination-treated conditions (Supplementary Fig. 6h–m).

Overall, these in vivo findings demonstrate that, compared with either drug alone, SR4835 in combination with CBDCA is significantly more effective at limiting and delaying in vivo tumor growth and dissemination in a highly aggressive PT-Res model, recapitulating the potent synergistic effects observed in vitro.

### CDK12i and PT reduce the expression of long coding genes and modulate DNA repair pathways

**Fig. 4:**
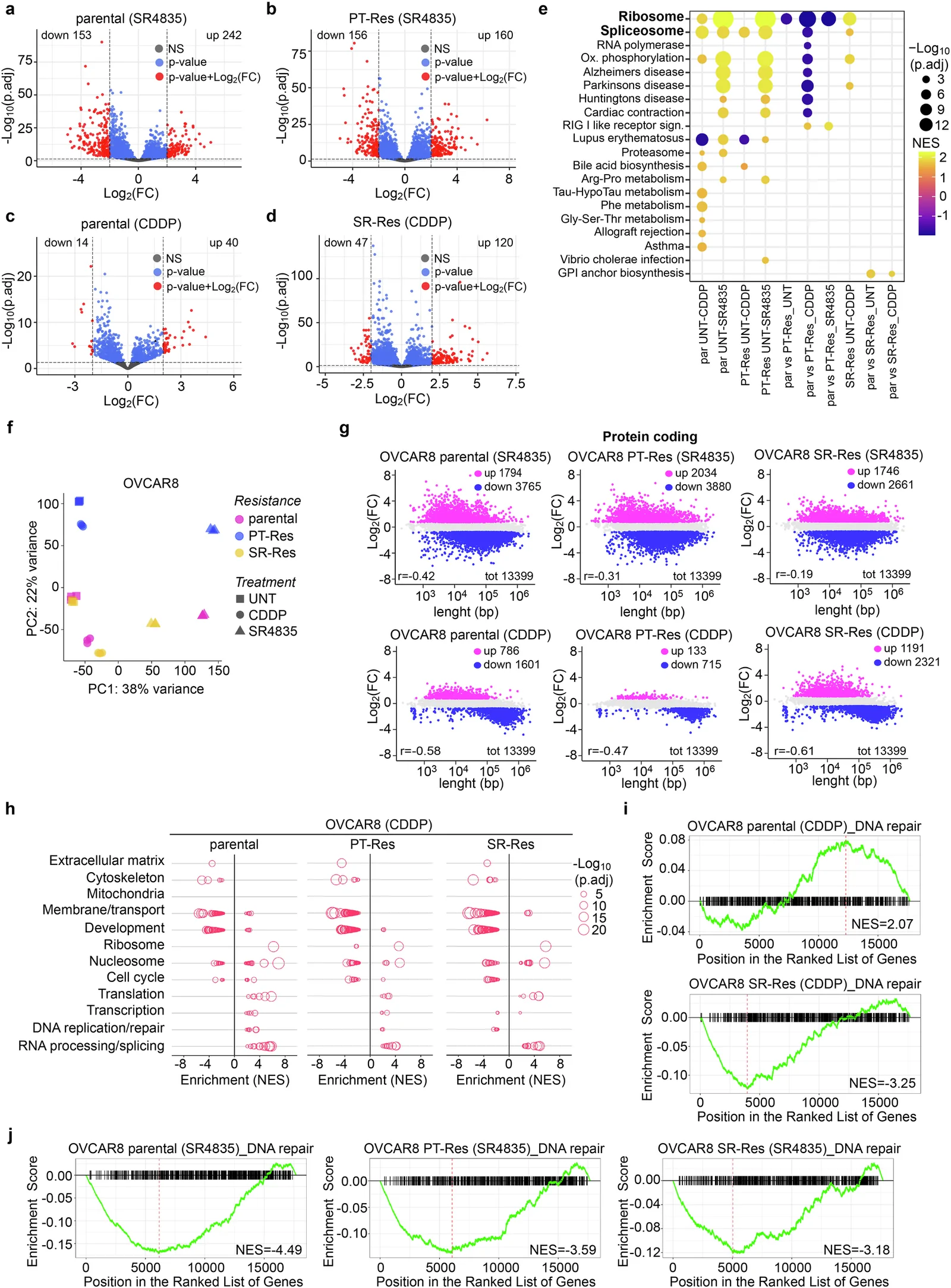
CDK12i and CDDP reduce long gene expression and modulate the splicing and ribosome pathways. **a**–**d** Volcano plots reporting the differentially expressed genes (DEGs) in SR4835-treated parental (**a**) and PT-Res (**b**) cells and in CDDP-treated parental (**c**) and SR-Res (**d**) cell lines, obtained from (*n* = 3) biological replicates per cell line and treatment condition. The data represent merged expression profiles from the indicated cell lines and were pooled according to their biological condition and treatment, as indicated in Supplementary Fig. 7a. Significant DEGs are reported in red (adjusted *p* value < 0.05 and Log_2_ of the fold change expression (FC) < -2 or >+2), blue (adjusted *p* value < 0.05), and gray (not significant). **e** KEGG pathway enrichment analysis, evaluating merged parental, PT-Res and SR-Res cells in the reported comparisons (see Supplementary Fig. 7a for a detailed list of the cell lines included). The graph shows the normalized enrichment score (NES) for the indicated gene sets that are significantly enriched (adjusted *p* value < 0.05). **f** Principal component analysis (PCA) of gene expression in untreated, SR4835-, or CDDP-treated OVCAR8 parental, PT-Res, and SR-Res cells. Each point represents one biological replicate (*n* = 3 per condition). **g** Scatterplots showing the Spearman’s rank correlation between the log fold change - log2(FC) - and gene length for genes whose expression differed between the OVCAR8 parental, PT-Res, and SR-Res cells and the untreated controls (*n* = 3 per condition). **h** GO biological process enrichment analysis of CDDP-treated OVCAR8 parental, PT-Res, and SR-Res cells relative to those of untreated controls (*n* = 3 per condition). GO terms are grouped into functional categories on the y-axis and plotted by normalized enrichment score (NES); each circle denotes a GO term, with size reflecting statistical significance. **i**–**j** Gene set enrichment analysis (GSEA) of DNA repair pathways showing the running enrichment score across the ranked gene list for OVCAR8 models treated with CDDP (panel **i**) or SR4835 (panel **j**), as indicated, each compared to untreated controls

Our findings indicated that CDK12i potentiates the activity of CDDP, suggesting that these two drugs impact interdependent molecular pathways. Both CDDP and CDK12i regulate gene expression by altering splicing and RNA processing, leading to transcriptional elongation defects.^18,50–52^ Whether this effect is shared across different models and whether it also occurs in PT-Res EOC remain largely unknown. On the basis of these findings and to identify the mechanism by which CDK12i enhances CDDP activity, we performed a genome-wide, high-coverage transcriptomic analysis across a broad panel of cellular models, including parental and PT-Res isogenic clones and primary EOC cell cultures, from different histotypes and genetic backgrounds, either untreated or under PT or SR4835 (Supplementary Fig. 7a). First, we assessed the correlation between gene length and gene expression following SR4835 or CDDP treatment. Compared with no treatment, both CDDP treatment and, especially, SR4835 treatment downregulated the expression of longer genes and upregulated the expression of shorter genes (Supplementary Fig. 7b). To identify transcriptional programs consistently associated with the platinum or SR4835 response while minimizing cell line-specific effects, we evaluated differential gene expression in sensitive/resistant models treated with PT or SR4835, which were grouped as indicated in Supplementary Fig. 7a. Compared with CDDP, SR4835 elicited more extensive transcriptional alterations in both the parental and PT-Res cell lines (Fig. 4a–c). CDDP affected the expression of very few genes in PT-Res cells, as expected, while it significantly altered the gene expression of SR-Res cells (Fig. 4d and Supplementary Fig. 7c–d), paralleling the reduced (PT-Res) and increased (SR-Res) sensitivity to CDDP. KEGG pathway and network analysis revealed that the ribosome and spliceosome pathways were significantly upregulated by SR4835 in the PT-sensitive and PT-Res models and by CDDP in the SR-Res cells, although the difference was less significant (Fig. 4e). The activity of the same pathways was downregulated in PT-Res cells compared with their PT-sensitive counterparts under all conditions, suggesting that these genes might play pivotal roles in the response to CDDP and CDK12i (Fig. 4e).

We next examined gene expression changes within individual isogenic cell line pairs, focusing on the OVCAR8 cell line, for which a comprehensive panel of sensitive and resistant models (parental, PT-Res, and SR-Res) was constructed. Principal component analysis (PCA) revealed clear segregation of PT-Res cells from both parental and SR-Res cells, which instead clustered closely together (Fig. 4f).

Pathway enrichment analysis, which involved the use of the DAVID platform (https://davidbioinformatics.nih.gov/home.jsp)^53,54^ and genes with the highest absolute PC2 loading values, revealed several molecular programs previously implicated in platinum resistance (Supplementary Fig. 7e–h).^40,55–57^ SR4835 treatment maximized transcriptional variance across all the models, with respect to what was observed with CDDP. However, compared with CDDP alone, CDDP more effectively separated the SR-Res cells from the parental cells (Fig. 4f). Analysis of gene expression as a function of gene length confirmed the trend observed in the global analyses across all the EOC cell lines. SR4835 treatment preferentially downregulated long genes and upregulated short genes in both parental and PT-Res cells, with a milder effect in the SR-Res models (Fig. 4g). Interestingly, CDDP also influenced gene expression according to gene length, particularly in the SR-Res and, to a lesser extent, in the parental cells, with a marginal effect in the PT-Res (Fig. 4g). Notably, this length-dependent transcription defect was observed for protein-coding genes, with a considerably lower extent for noncoding RNAs (Supplementary Fig. 7i-j). Gene set enrichment analysis (GSEA) of biological processes (GO terms) in the OVCAR8 models confirmed the KEGG pathway results obtained from the global analysis and revealed additional insights. CDDP treatment resulted in a positive normalized enrichment score (NES) for genes involved in DNA replication/repair in parental cells, suggesting an adaptive response to treatment-induced damage (Fig. 4h–i). PT-Res cells displayed a similar, albeit weaker, trend, supporting the notion that they have already adapted their PT response through alternative DNA repair mechanisms (Fig. 4h). Interestingly, CDDP treatment led to a negative NES of DNA repair-related genes in SR-Res cells (Fig. 4h–i), possibly explaining their hypersensitivity to CDDP. Conversely, SR4835 treatment negatively affected the DNA repair pathway (Fig. 4j) across all the evaluated models, with a consistent negative NES proportional to their relative resistance to SR4835.

Collectively, our findings are consistent with those of previous reports supporting a model in which CDK12 inhibition induces transcriptional elongation defects,^19,58–61^ which primarily affect long coding genes, with shorter genes displaying normal or increased expression and markedly associated with a negative enrichment of DNA repair pathways.

### CDK12i affects the distribution of splicing events in both sensitive and resistant EOC models

**Fig. 5:**
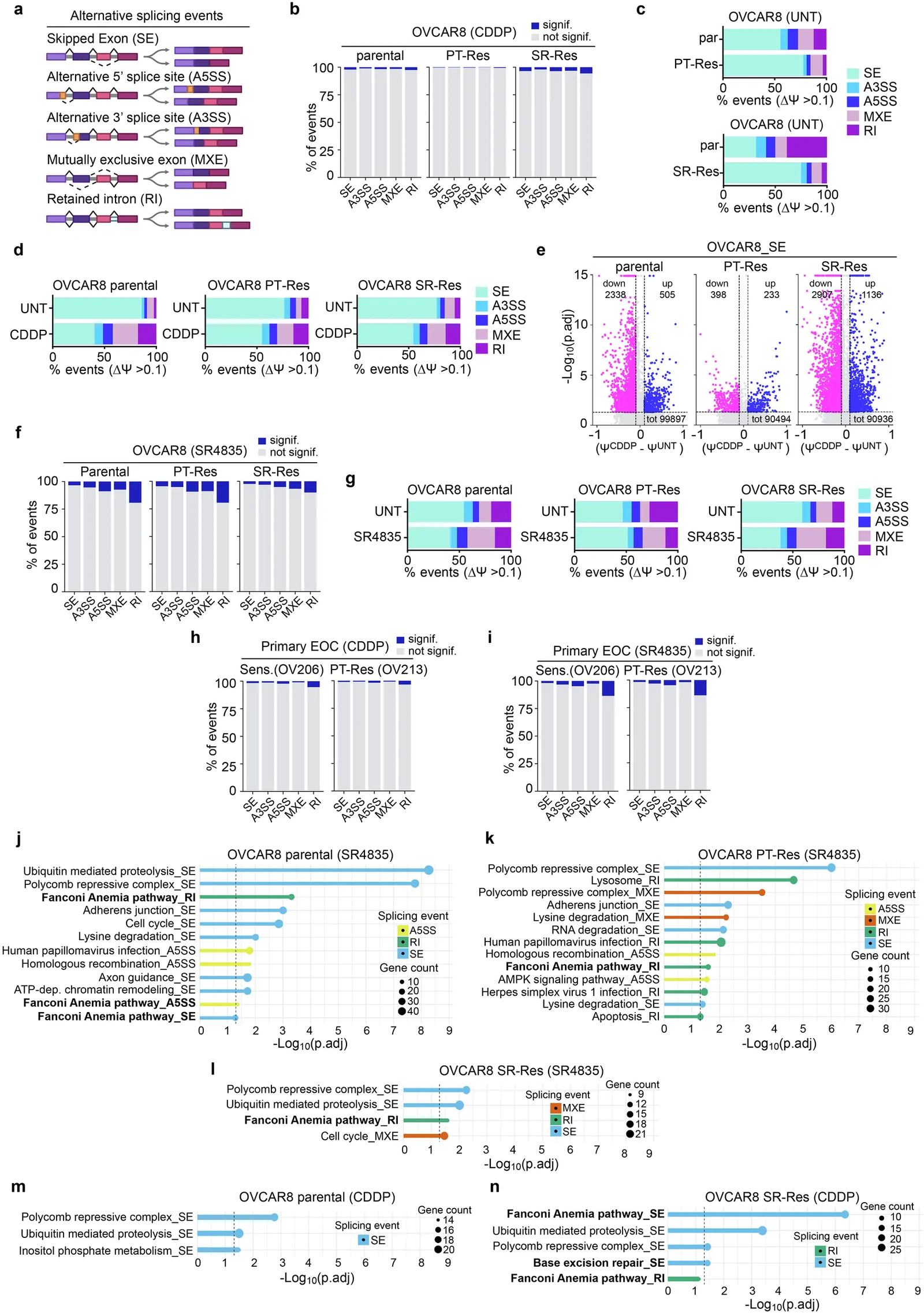
Effects of CDK12i and CCDP on alternative splicing events, modulating DDR pathways. **a** Schematic representation of alternative splicing events. (created with BioRender.com and adapted from “mRNA Splicing Types”, by BioRender.com (2025). Retrieved from https://app.biorender.com/biorender-templates. **b** Graphs representing the percentages of significantly altered splicing events (blue) with respect to all other events (gray) in CDDP-treated parental, PT-Res and SR-Res OVCAR8 cells for all annotated splicing events. Percentages are calculated for all mapped splicing events. The data represent *n* = 3 biological replicates. **c**, **d** Graphs showing the percentage of significantly altered alternative splicing events with increased spliced-in ratios (ΔΨ>0.1) across splicing event categories relative to the total number of events, in each condition, comparing OVCAR8 parental cells with either PT-Res or SR-Res counterparts (**c**) or with their untreated counterparts (**d**). **e** Volcano plots of skipped-exon (SE) alternative splicing events in CDDP-treated OVCAR8 parental, PT-Res, and SR-Res cells compared with those in untreated controls. The x-axis shows the spliced-in ratio difference between conditions (Ψ^CDDP^ − Ψᵁᴺᵀ), with significant events colored blue or magenta for positive or negative differences, respectively (*n* = 3 biological replicates). **f** Graphs representing the percentages of significantly altered splicing events (blue) with respect to all other events (gray) in untreated and SR4835-treated parental, PT-Res and SR-Res OVCAR8 cells. Percentages are calculated for all mapped splicing events. The data represent *n* = 3 biological replicates. **g** Graphs showing the percentage of significantly altered alternative splicing events with increased spliced-in ratios (ΔΨ>0.1) across splicing event categories, relative to the total number of events, in each condition, comparing SR4835-treated parental, PT-Res, and SR-Res OVCAR8 cells to their untreated counterparts (*n* = 3 biological replicates). **h**, **i** Graphs representing the percentages of significantly altered splicing events (blue) with respect to all other events (gray) in CDDP-treated (**h**) and SR4835-treated (**i**) OV206 and OV213 primary cells for all annotated splicing events. Percentages are calculated for all mapped splicing events. The data represent *n* = 3 biological replicates. **j**–**n** DAVID pathway enrichment analyses using gene lists of significantly altered splicing events in each category, showing enrichment for gene sets in untreated and SR4835-treated OVCAR8 parental (**j**), PT-Res (**k**), and SR-Res cells (**l**) and CDDP-treated OVCAR8 parental (**m**), and SR-Res cells (**n**). Significance was defined as a Benjamini–Hochberg FDR < 0.05 (*n* = 3 biological replicates)

Splicing regulation emerged as one of the most significantly modulated pathways in our transcriptomic analysis. High-coverage (>60 million reads as a mean across samples) RNA-seq allowed us to evaluate not only differences in gene expression profiles but also to characterize which types of alternative splicing (AS) (Fig. 5a), if any, were modified in the different EOC models and whether they could account for the differences in treatment sensitivity. We thus comprehensively assessed the distribution of AS events in parental, PT-Res, and SR-Res models, focusing on their possible modulation by either CDDP or SR4835.

For each comparison, the fraction of the significant splicing events (ΔΨ > 0.1) was quantified among all mapped splicing events, with the ΔΨ parameter denoting the difference in percent spliced in (Ψ) transcripts in a particular spliced region between conditions under comparison (see Methods for details). Overall, CDDP treatment resulted in a few significantly altered splicing events in the parental PT-sensitive models (Fig. 5b and Supplementary Fig. 8a–b). This was consistent for all the different types of AS events considered, with retained introns (RIs) showing the greatest fraction of significantly altered events upon CDDP treatment (Fig. 5b and Supplementary Fig. 8a). Among the different cell types, TOV112D cells (derived from an endometrioid EOC) presented the lowest number of significant events for each splicing category, whereas OVSAHO cells (likely from high-grade serous ovarian cancer, HGSOC) presented the greatest number of events (Supplementary Fig. 8a). The fraction of significant splicing events markedly decreased in PT-Res isogenic clones across all categories of AS events analyzed under PT treatment (Fig. 5b and Supplementary Fig. 8a). Conversely, compared with the parental PT-sensitive or PT-Res counterparts, the percentage of altered AS events was markedly greater in the SR-Res cells, with RI events being particularly affected (Fig. 5b and Supplementary Fig. 8b). These findings suggest that the extent of significant CDDP-induced splicing alterations is correlated with the intrinsic CDDP sensitivity of each cell line.

We then quantified the relative distribution of the significant ΔΨ events for each AS category, normalized by the total number of significant events across AS categories identified in each condition, allowing direct comparison across different cellular models and treatment conditions. In OVCAR8 cells, compared with parental cells, PT-Res cells displayed a basal prevalence of SE and a reduced fraction of all other splicing categories (Fig. 5c). The same altered distribution fraction was even more pronounced in the untreated SR-Res cells (Fig. 5c). Similar results were observed in the COV362 SR-Res model (Supplementary Fig. 8c). CDDP treatment mainly reduced the percentage of SE events and increased those of all the other events across all the OVCAR8 models. Interestingly, it had the most significant effect on parental and SR-Res cells, as also shown in the corresponding volcano plots (Fig. 5d–e). Although the PT-Res models exhibited fewer significant CDDP-induced events, which is consistent with their resistance phenotype (Fig. 5b and Supplementary Fig. 8a–b), CDDP induced similar patterns in the overall percentage distribution of splicing events across all parental PT-sensitive and PT-Res cell lines (Supplementary Fig. 8d–e).

Compared with CDDP, SR4835 had a greater effect on the fraction of significantly altered splicing events in all the tested models, overall showing a more pronounced effect on splicing regulation (Fig. 5f and Supplementary Fig. 8f–g). The SR-Res models had less variation in terms of splicing events, which is consistent with their resistance to SR4835 (Fig. 5f and Supplementary Fig. 8g). Looking specifically at the percentage distribution of the events normalized to the total number of significantly altered events, SR4835 increased mainly the MXE and decreased the RI across all PT-sensitive and PT-Res models, with a weaker impact on the other alternative splicing categories (Fig. 5g and Supplementary Fig. 8h). In contrast, compared with control cells, SR-Res cells under SR4835 treatment presented an increase in the number of RI events (Fig. 5g and Supplementary Fig. 8i), suggesting that resistance to SR4835 is not only associated with fewer significant splicing events but also impacts the distribution of individual splicing categories differently. These patterns were independently validated in patient-derived primary cultures obtained from an HGSOC patient at different stages of the disease, where the PT-sensitive OV206 cells exhibited a greater proportion of AS events upon CDDP treatment, compared with PT-Res OV213 cells, whereas SR4835-treated cells exhibited a comparable pattern (Fig. 5h–i).

We next examined whether splicing alterations could influence specific pathways and whether these pathways were commonly modulated by CDDP and CDK12i. Using the DAVID bioinformatics platform,^53,54^ we observed that SR4835-altered splicing events significantly affected specific DDR signaling pathways. Among them, the Fanconi anemia pathway was enriched across multiple splicing event types. Similarly, the alteration of splicing in genes belonging to the Fanconi anemia pathway paralleled the cell sensitivity to PT, with PT-Res cells being the least affected (Fig. 5j–l). Notably, specific signaling pathways were modulated only in the SR-Res models. Among these, SE events were significantly enriched in genes associated with Fanconi anemia and base excision repair (BER) in both OVCAR8 and COV362 SR-Res cells treated with CDDP (Fig. 5m-n and Supplementary Fig. 8j-k). In contrast, CDDP treatment did not significantly modulate BER in parental or PT-Res cells, suggesting that the sensitivity of SR-Res cells to CDDP is increased in response to alterations in this pathway (Fig. 5n and Supplementary Fig. 8k). Detailed information on the genes contributing to these enrichment scores, including their association with homologous recombination and gene length, is reported in Supplementary Table 5.

Overall, these data demonstrate that both CDDP and CDK12i regulate distinct subsets of alternative splicing events involving genes involved in DNA repair pathways. Notably, the magnitude of splicing alterations on factors belonging to the Fanconi anemia pathway, possibly converging on homologous recombination downstream dysregulation, correlated with differential sensitivity to SR4835 or CDDP. These findings further support the functional involvement of AS mechanisms in shaping the dynamic cellular response to both CDDP and CDK12i.

### CDK12 interacts with the SFPQ/p54^nrb^ splicing complex to regulate the response to PT

**Fig. 6:**
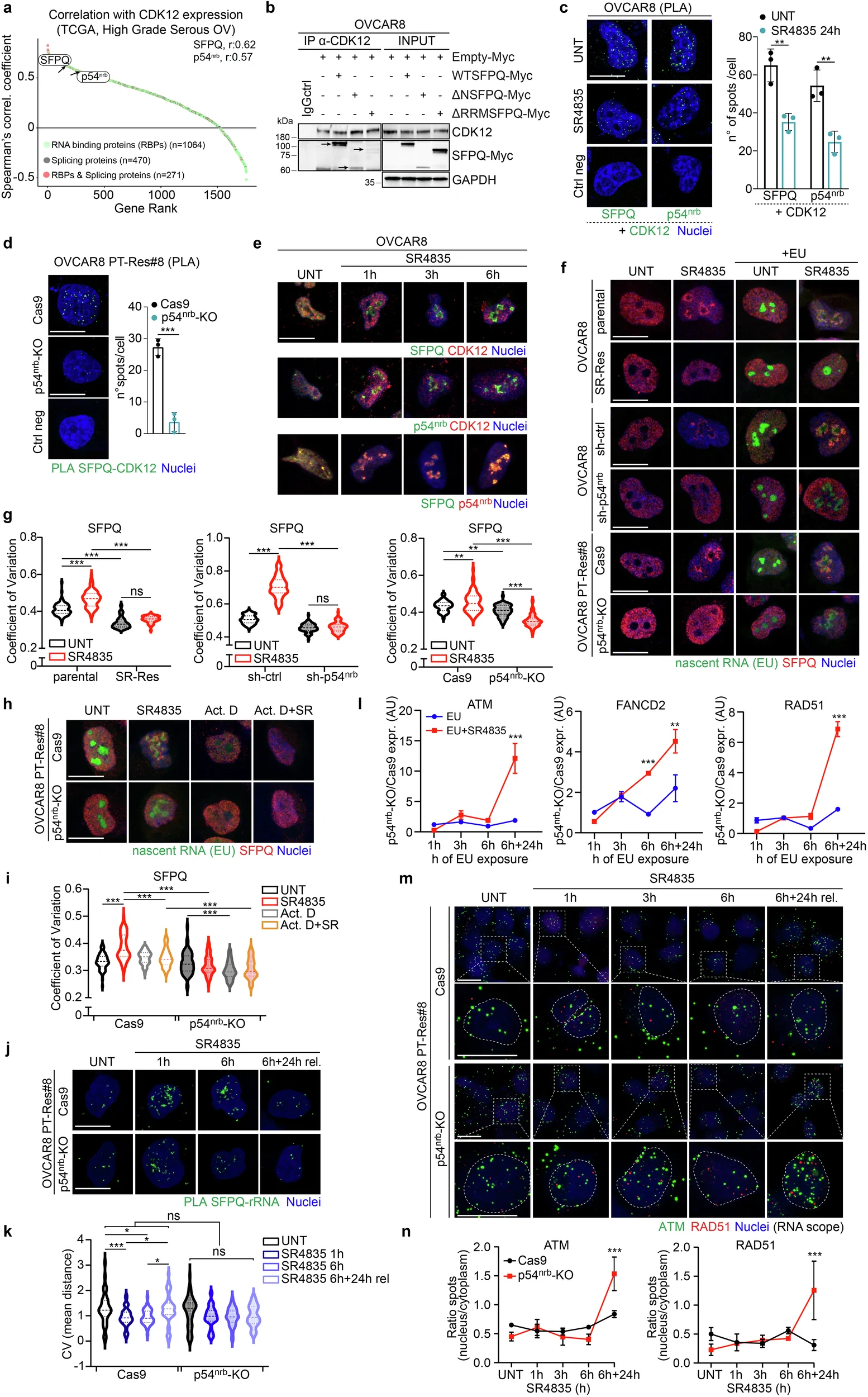
CDK12 interacts with the SFPQ/p54^nrb^ splicing complex. **a** Waterfall plot of Spearman’s correlation coefficients in the TCGA-HGSOC cohort between the expression of CDK12 and the expression of a set of splicing and/or RNA-binding proteins (RBPs), ranked by correlation *p* value. **b** Coimmunoprecipitation (co-IP) analysis of the interaction between CDK12 and SFPQ in OVCAR8 cells overexpressing SFPQ WT or Myc-tagged mutants. GAPDH was used as a loading control. The arrows indicate the expected molecular weight positions of the corresponding SFPQ constructs. **c**, **d** Representative images and quantification of the results of proximity ligation assays (PLAs) for SFPQ-CDK12 and p54^nrb^-CDK12 in OVCAR8 cells treated with SR4835 (300 nM) for 24 h (**c**). (**d**) PLA between SFPQ-CDK12 in Cas9- or p54^nrb^-KO#26 (hereafter p54^nrb^-KO) OVCAR8 PT-Res#8 (hereafter OVCAR8 PT-Res) cells. In (**c**) and (**d**), the graphs report the number of PLA dots/cell for each condition, expressed as the mean ± SD from *n* = 3 biological replicates. Two-tailed, unpaired Student’s t tests were used. Scale bars, 20 μm. **e** IF of SFPQ, CDK12 and p54^nrb^ localization in OVCAR8 parental cells treated with SR4835 (300 nM) for 1, 3 or 6 h. TO-PRO-3 iodide was used to counterstain the nuclei. Scale bar of 20 μm. **f** IF of SFPQ localization with or without EU-labeled newly transcribed RNAs costained with parental, SR-Res, and p54^nrb^-silenced (sh-p54^nrb^) or -KO (p54^nrb^-KO) OVCAR8 cells treated with SR4835 (300 nM) for 6 h ± 5-ethynyl uridine (EU, 1 mM, 1 h). Scale bars, 20 μm. **g** Coefficient of variation (CV) quantification of the SFPQ signal under SR4835 (300 nM, 6 h) treatment compared with the untreated condition in the images reported in (**f**). The graphs represent the mean CV values ± SDs of *n* = 3 biological replicates. An increase in the CV represents signal clustering, whereas a lower CV indicates a dispersed signal. **h**, **i** IF of SFPQ colocalization costained with EU-labeled newly transcribed RNAs in p54^nrb^-KO or control (Cas9) OVCAR8 PT-Res cells treated with either actinomycin D (50 ng/mL, 1 h), SR4835 (300 nM, 6 h) or their combination. Scale bars, 20 μm. Relative CV quantification (**i**) of SFPQ and newly transcribed RNA signals, showing that actinomycin D impairs SFPQ clustering under SR4835. **j**, **k** PLA between SFPQ and ribosomal RNA (rRNA) in Cas9 and p54^nrb^-KO OVCAR8 PT-Res cells treated with SR4835 (300 nM) for 1 or 6 h and released in SR4835-free medium for 24 h (6 h + 24 h rel.). Scale bars, 20 μm. The relative CV of the SFPQ-rRNA PLA signal showed impaired spot clustering under SR4835 in p54^nrb^-KO cells compared with that in Cas9 control cells (**k**). **l** Ratio of p54^nrb^-KO/Cas9 OVCAR8 PT-Res relative abundance of EU-labeled and isolated ATM, FANCD2 and RAD51 transcripts following 1, 3 or 6 h of EU (1 mM) exposure±SR4835 (300 nM, 6 h), followed by release in EU- and SR4835-free medium for 24 h (6 h + 24 h). Data represent the mean ± SD of *n* = 3 independent biological replicates as arbitrary units (AU, 2^(-ΔCt)^). **m**, **n** RNAscope in situ hybridization images (**l**) of ATM and RAD51 transcripts in Cas9 and p54^nrb^-KO OVCAR8 PT-Res cells treated with SR4835 (300 nM) for 1, 3 or 6 h and released in SR4835-free medium for 24 h (6 h + 24 h rel.). The nuclear/cytoplasmic spot ratio (**m**) indicated greater nuclear accumulation of ATM and RAD51 transcripts in p54^nrb^-KO cells than in Cas9 control cells after treatment release. Data represent the mean ± SD of *n* = 3 independent biological replicates. Scale bars, 20 μm. Statistical significance was calculated using ordinary one-way ANOVA (**g**, **i**, **k**, **l** and **n**) and is expressed as follows: *p < 0.05, **p < 0.01, and ***p < 0.001

The ribosome and spliceosome pathways were upregulated by CDK12i in both the PT-sensitive and PT-Res models (Fig. 4e). Emerging evidence highlights that CDK12 and CDK13 may interact with and phosphorylate RNA processing factors, affecting their functions and recruitment to RNA polymerase II,^18^ although the molecular and functional implications of these interactions remain unclear. To gain some insights, we first investigated the ovarian cancer transcriptomic database of the TCGA and evaluated the correlation between CDK12 expression and a comprehensive repertoire of RNA-binding proteins (RBPs), as annotated by Hentze et al.^62^ and available splicing-related gene sets, on the basis of the hypothesis that coexpressed genes could also be functionally linked. This analysis revealed a strong correlation between CDK12 and different RBPs, among which the SFPQ/p54^nrb^ complex was among the most significantly correlated (Fig. 6a). The SFPQ/p54^nrb^ complex is a core component of the RNA processing machinery and is involved in the regulation of gene expression at multiple levels, including the transcription of long genes,^33,63^ genes involved in RNA splicing and the DDR,^30^ all of which are also regulated by CDK12 activity.^11^ SFPQ was previously identified as a key regulator of PT response and sensitivity in EOC.^29^ Interestingly, using STRING-based network enrichment analyses of genes altered in our RNA-seq data, we identified several densely connected gene clusters that interconnect the RNA-processing KEGG ribosome and spliceosome pathways. SFPQ, along with other splicing factors, emerged among the CDK12i-modulated genes in this network (Supplementary Fig. 9a). Collectively, these experimental findings and data from the literature support the possibility that CDK12 and SFPQ are not only coexpressed but also functionally interact in EOC. We thus investigated whether the SFPQ/p54^nrb^ complex cooperates functionally with CDK12 to regulate transcription and/or splicing in PT-sensitive and PT-Res EOC cells. In silico modeling with AlphaFold3^64^ predicted that CDK12 could bind the SFPQ/p54^nrb^ complex through multiple hydrogen bonds between CDK12 and SFPQ residues (Supplementary Fig. 9b). Consistently, coimmunoprecipitation confirmed an interaction between CDK12 and SFPQ/p54^nrb^, both at the endogenous level and upon overexpression of SFPQ and/or CDK12 (Fig. 6b and Supplementary Fig. 9c–e). Analysis of OVCAR8 and 293 T/17 cells overexpressing SFPQ mutants lacking key RNA, DNA, or protein interaction domains (WT, ΔN, and ΔRRM) revealed that CDK12 association is reduced in the presence of both mutants compared with that in the WT counterpart, indicating that the full SFPQ structure is required for efficient interaction (Fig. 6b and Supplementary Fig. 9c–d). Conversely, overexpression of CDK12 truncation mutants^65^ confirmed that SFPQ binding to all the constructs, indicating that the N-terminal RS domain is sufficient for the interaction, whereas neither the kinase domain (KD) nor the proline-rich motif (PRM) is required (Supplementary Fig. 9e). These interactions were further supported by proximity ligation assays (PLAs) in several EOC cell lines (Fig. 6c and Supplementary Fig. 9f–g). This signal predominantly occurred in the nucleus and markedly decreased after 24 h of CDK12i treatment, which is consistent with the CDK12 proteasomal degradation induced by SR4835. Conversely, PT treatment increased the CDK12-SFPQ interaction in COV318 cell nuclear extracts (Supplementary Fig. 9h).

By in silico prediction of intrinsically disordered regions (IDRs), we observed that both CDK12 and SFPQ are highly disordered proteins, whereas p54^nrb^ has only a few predicted IDRs (Supplementary Fig. 9i). Many transcriptional and splicing regulators contain IDRs, which are fundamental for orchestrating the dynamics of nuclear membrane-less organelles involved in transcription and RNA processing via multivalent charge interactions.^66^ Looking for possible molecular recognition features or elements (MoRFs)^67^ that can mediate protein–protein interactions in CDK12-SFPQ/p54^nrb^ IDRs, we identified several MoRFs of 30 residues or fewer in CDK12 and in two distinct regions of SFPQ and p54^nrb^ (Supplementary Fig. 9j). These regions, included in the AlphaFold3 CDK12-SFPQ/p54^nrb^ binding prediction, could be responsible for the low confidence metrics of the model, which is consistent with the limitations of AlphaFold in predicting interactions within the IDRs of full-length proteins.^68,69^ The relatively low degree of disorder in p54^nrb^ (Supplementary Fig. 9i-j) is consistent with its role as a molecular scaffold mediating multiple protein‒protein interactions, which are essential for gene expression regulation.^70^ Given that stable SFPQ knockdown is not compatible with EOC cell survival,^29^ we assessed the impact of p54^nrb^ genetic abrogation on the functional CDK12-SFPQ interaction. PLA in a PT-Res p54^nrb^-KO clone revealed a markedly reduced CDK12-SFPQ interaction compared with that in control cells expressing p54^nrb^ (Fig. 6d). These observations reinforce the hypothesis that the CDK12-SFPQ/p54^nrb^ complex, possibly formed through dynamic, three-dimensional interactions within ordered and IDR regions, is stabilized by the presence of p54^nrb^ (Supplementary Fig. 9i-j). Additionally, compared with control cells, p54^nrb^-silenced cells exhibited reduced sensitivity to CDK12i and increased sensitivity to CDDP (Supplementary Fig. 10a–b). Interestingly, this pattern recapitulated the phenotype of SR-Res cells, which are resistant to CDK12i and more sensitive to PT (Fig. 2d–e).

Intrinsically disordered regions and their charge-mediated interactions play critical roles in the liquid‒liquid phase separation of transcription factors within condensates, which in turn regulate transcription under both physiological and stress conditions.^66^ We thus evaluated whether CDK12 could cocompartmentalize with SFPQ/p54^nrb^ in similar condensates and whether CDK12i altered their localization. Using the experimental approach reported by Yasuhara et al.,^27^ we observed that the CDK12-SFPQ/p54^nrb^ interaction occurs in specific nuclear regions at the basal level and that CDK12i-induced SFPQ and p54^nrb^ shift to nuclear condensates, from which CDK12 appeared to be excluded (Fig. 6e and Supplementary Fig. 10c). Next, to detect SFPQ/p54^nrb^ relocalization upon CDK12i treatment, we used a milder permeabilization protocol that preserved cytoplasmic integrity (see Materials and Methods for details). In addition, to monitor global transcriptional activity, cells were labeled with 5-ethynyl uridine (EU), which is incorporated into newly transcribed RNA^71^ and accumulates mainly in nucleolar ribosomal RNA (rRNA)^72^ (Fig. 6f). Treatment with SR4835 caused a pronounced reduction in EU incorporation, which was consistent with decreased RNA polymerase II transcriptional activity under CDK12i, in all OVCAR8 models but not in the SR-Res counterpart (Fig. 6f), as supported by the relative quantification of newly transcribed RNA (Supplementary Fig. 10d). Interestingly, compared with their control counterparts, p54^nrb^-silenced or p54^nrb^-KO cells presented lower basal EU incorporation, indicating reduced transcriptional activity in the absence of p54^nrb^, which was further decreased by SR4835 treatment (Fig. 6f and Supplementary Fig. 10d). The residual EU incorporation detected in the nucleolar RNAs was spatially redistributed upon CDK12i and associated with SFPQ/p54^nrb^ condensates in parental and p54^nrb^-WT cells but not in SR-Res cells, as also supported by the relative quantification of their signal redistribution (Fig. 6f–g and Supplementary Fig. 10d–f) and by the coefficient of variation, whose lower values reflected a diffuse distribution of the signal, while higher values indicated signal clustering^73^ (Fig. 6g and Supplementary Fig. 10g–h). In line with previous observations, p54^nrb^-silenced and p54^nrb^-KO cells displayed a relatively high basal SFPQ signal intensity that did not concentrate in proximity to nucleoli with redistributed nucleolar RNAs upon CDK12i treatment (Fig. 6f–g). These results show that p54^nrb^ is required for the CDK12-SFPQ interaction in EOC models, independent of PT sensitivity, thereby rendering CDK12i largely ineffective for cell killing and SFPQ relocation if p54^nrb^ expression is abrogated.

Given the observed nucleolar RNA relocalization upon CDK12i in all the tested models, we next investigated whether the SFPQ condensates resulted solely from RNA polymerase II impairment or also required active rRNA biogenesis. When a low dose of actinomycin D, a DNA intercalating agent that impairs RNA polymerase I-dependent rRNA transcription,^74,75^ alone or in combination with SR4835, the SFPQ signal remained diffuse and was accompanied by reduced EU incorporation (Fig. 6h–i and Supplementary Fig. 10i) in the nucleolus, indicating that SFPQ relocalization is a specific response to CDK12i and is contingent on active rRNA biogenesis by RNA polymerase I. Consistently, p54^nrb^-KO completely abrogated SFPQ relocation under SR4835±actinomycin D (Fig. 6h–i). These results were further confirmed using CX-5461, a potent and specific inhibitor of rRNA synthesis^76^ (Supplementary Fig. 10j). PLA confirmed the spatial proximity of SFPQ-rRNA, which increased in clustering upon SR4835 treatment (Fig. 6j–k). This finding was also consistent with the reduced spatial aggregation of SFPQ-rRNA PLA spots under CDK12i in p54^nrb^-KO cells, concomitant with a fully reverted distribution after drug washout (Fig. 6j–k).

Finally, we overexpressed SFPQ mutants lacking key RNA, DNA, or protein interaction domains in OVCAR8 cells. The SFPQ-ΔRRM mutant, lacking both RNA-recognition motifs, did not cluster into condensates after CDK12i treatment, whereas SFPQ-WT and SFPQ-ΔN, which lack the N-terminal region and part of the DNA-binding domain, were still able to cluster correctly (Supplementary Fig. 10k). These findings suggest a potential role for the RNA-binding domain of SFPQ, although further validation is needed. Collectively, these results support a model in which SFPQ physically associates with CDK12, and its binding to CDK12i is dependent on its interaction with nucleolar ribosomal RNA.

### Loss of p54^nrb^ recapitulates the effect of CDK12i on DDR gene expression

Given the roles of p54^nrb^ in transcription termination, mRNA nuclear export and the DNA damage response^30^ and the observed increased sensitivity of p54^nrb^-silenced cells to CDDP (Supplementary Fig. 10a), we investigated the functional effects of p54^nrb^ loss on established CDK12 target genes involved in the DNA damage response.^18^ Interestingly, Western blot analysis of p54^nrb^-deficient EOC cells revealed reduced protein expression of specific DDR genes, such as ATM and FANCD2, which was similar to what was observed following CDK12 silencing or inhibition (Supplementary Fig. 10l). To understand whether and at which transcriptional level p54^nrb^ regulates these genes, we isolated newly transcribed RNAs from p54^nrb^-KO and control cells treated with SR4835 to determine the transcription dynamics of EU-labeled ATM, FANCD2, and RAD51 transcripts. The amount of EU-labeled RNAs was comparable between p54^nrb^-WT and p54^nrb^-KO cells concomitantly with pulse-chase EU exposure. Interestingly, this trend differed in the SR4835-chase condition (24 h of release), in which p54^nrb^-KO cells retained significantly higher levels of EU-marked transcripts than their WT counterparts did (Fig. 6l).

Combining these results with the reduced DDR protein expression observed in p54^nrb^-deficient cells, we suggest that the excess of EU-labeled RNA in the absence of p54^nrb^ reflected increased amounts of nuclear-retained, untranslated RNAs, which are then released following CDK12i washout (Fig. 6l). To address this possibility, we evaluated the spatial localization of ATM and RAD51 transcripts at the basal level, upon CDK12i treatment and under release conditions (24 h chase), by an RNA scope assay. CDK12i reduced the levels of labeled ATM and RAD51 transcripts similarly in both p54^nrb^-KO and control cells; however, following SR4835 washout, p54^nrb^-KO cells presented markedly increased nuclear/cytoplasmic ratios of both RAD51 and ATM transcripts (Fig. 6m–n). Together, these data support that the SFPQ/p54^nrb^ splicing complex cooperates with CDK12 to modulate the functional expression and subcellular localization of long DDR genes.

### In vitro and in vivo characterization of CT7439, a first in human CDK12i

**Fig. 7:**
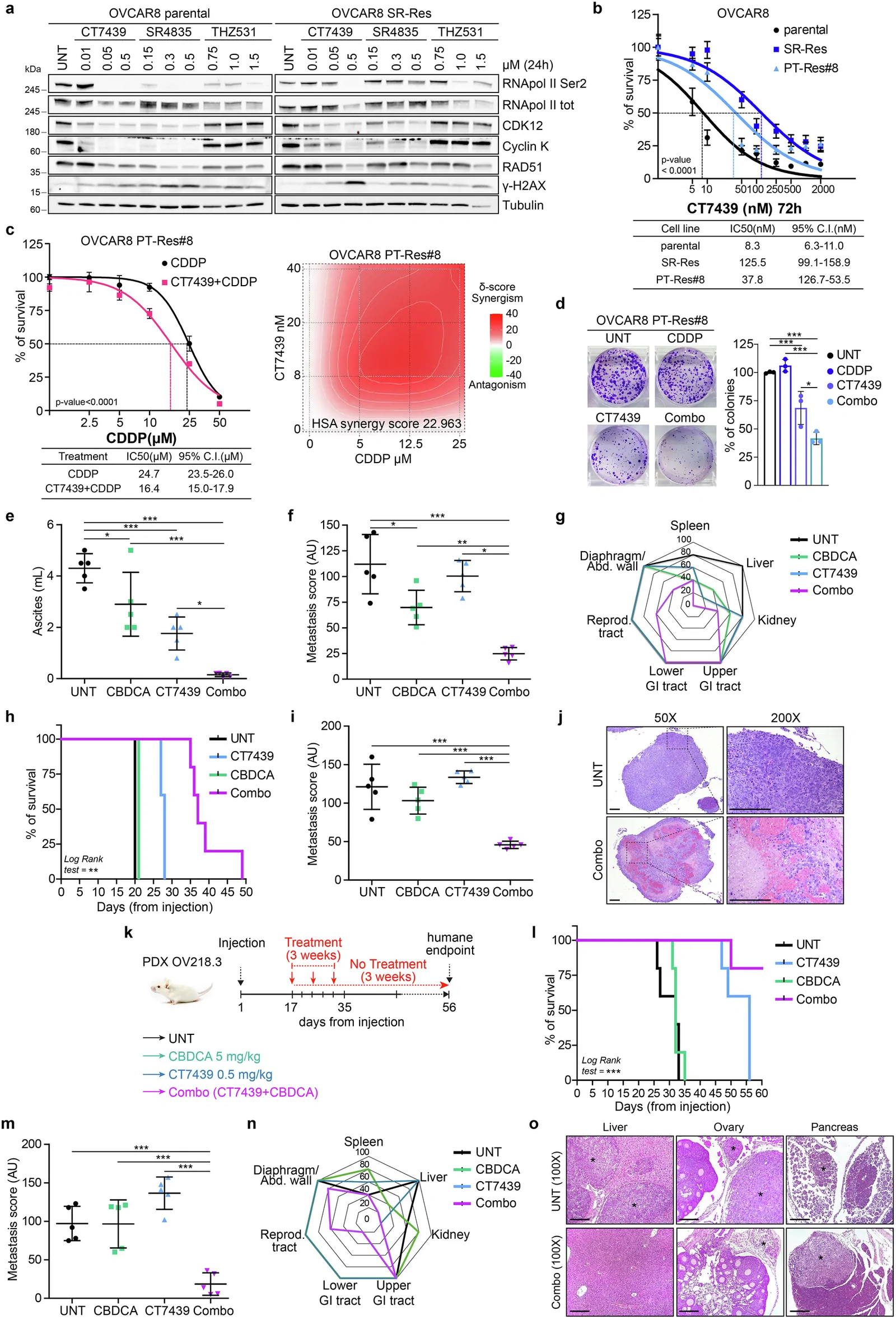
In vitro and in vivo efficacy of CT7439 in impairing EOC cell viability and in combination with PT. **a** WB was used to evaluate the expression of the indicated proteins in OVCAR8 parental and SR‑Res cells treated with increasing doses of CT7439, SR4835 and THZ531 for 24 h. Tubulin was used as a loading control. **b** Nonlinear regression analysis of cell viability in parental, PT-Res, and SR-Res OVCAR8 cells treated with increasing doses of CT7439 for 72 h. The data are expressed as the percentage of viable cells relative to that of the untreated cells and represent the mean ± SD (*n* = 3 biological replicates); the IC50 and confidence interval (CI) are reported in the table. **c** Nonlinear regression evaluation of cell viability in OVCAR8 PT-Res cells pretreated with ±CT7439 (7.5 nM) for 16 h and then treated with increasing doses of CDDP for 48 h. The data are expressed as the percentage of viable cells relative to that of the untreated cells and represent the mean ± SD (*n* = 3 biological replicates); the IC50 and confidence interval (CI) are reported in the table. On the right, two-dimensional synergy maps showing synergistic and antagonistic regions in red and green, respectively. Darker red indicates maximal synergy. HSA values > 10 indicate synergy, whereas values <−10 indicate antagonism. **d** Representative images and quantification of the results of the clonogenic assay in OVCAR8 PT-Res cells pretreated with ±CT7439 (5 nM) for 16 h, followed by treatment with CDDP (15 μM, 3 h). The data represent the mean ± SD; *n* = 3 independent experiments. **e**–**g** Graph showing the ascites volume (**e**), quantification (**f**) and radar plot (**g**) of abdominal lesions in C57BL/6 mice injected with ID8 p53^−/−^ PTEN^−/−^ syngeneic cells and treated with the regimen illustrated in in Supplementary Fig. 12a (*n* = 5 mice per condition). **h**–**i** Kaplan‒Meier survival curves (**h**) and distribution of abdominal metastasis (**i**) in C57BL/6 mice injected with ID8 p53^−/−^ PTEN^−/−^ murine ovarian cells and treated with the regimen illustrated in Supplementary Fig. 12f (*n* = 5 per condition). **j** H&E staining of tumor masses within the peritoneal cavity of mice treated as described in Supplementary Fig. 12f, showing wider necrotic (pink) hemorrhagic (red) areas in the combined treatment group than in the untreated group at 50× and 200× magnification. Scale bar is 400 μm. **k**‒**n** Kaplan‒Meier survival curves (**l**), graph of the quantification (**m**) and radar plot (**n**) of abdominal metastasis in NSG mice injected with PDX#OV218.3 and treated according to the scheme depicted in (**k**) (*n* = 5 per condition). **o** H&E staining of tumor masses within the liver, ovary and pancreas of NSG mice injected with PDX#OV218.3 and treated according to the scheme depicted in (**k**) at 100× magnification. Scale bar is 400 μm. Compared with vehicle-treated mice, Combo-treated mice showed reduced infiltration and preserved organ architecture. Statistical significance was assessed using Fisher’s exact test (**b** and **c**), one-way ANOVA (**d**, **e**, **f**, **i** and **m**) or the log-rank (Mantel‒Cox) test (**h** and **l**), and the results are expressed as follows: *p < 0.05, **p < 0.01, and ***p < 0.001

To support a possible clinical translation of our results, we used CT7439 (Carrick Therapeutics), the first CDK12i to enter clinical evaluation, which is currently in phase 1 (NCT06600789). CT7439 is a small-molecule kinase inhibitor and acts as a monovalent “glue degrader” of Cyclin-K, the obligate partner of CDK12/13. This dual mechanism of action significantly increases potency and is reported to effectively inhibit DNA repair at the transcriptional level.^26^ In a panel of 25 different cell lines and primary cultures, CT7439 was more potent than both THZ531 and SR4835 (Supplementary Fig. 11a). The biological efficacy of CT7439 was confirmed at the molecular level by a strong reduction in the expression of Cyclin-K and DDR genes, especially RAD51, thereby activating DNA damage at nanomolar concentrations, as well as in PT-Res models (Supplementary Fig. 11b–e). Notably, compared with the other CDK12is, CT7439 demonstrated greater efficacy in downregulating RNA Pol II Ser2 phosphorylation and Cyclin K and RAD51 expression at low nanomolar doses in both parental and SR-Res OVCAR8 cells (Fig. 7a). Furthermore, the nanomolar concentration of CT7439 resensitized PT-Res cells to CDDP and, to a lesser extent, to the PARPi (niraparib) when it was used in a sequential schedule (Fig. 7c–d and Supplementary Fig. 11f). These results confirm and further highlight the synergistic effect observed with SR4835 in combination with CDDP (Fig. 7c and Fig. 1j). Notably, CT7439 treatment clearly induced synergy with niraparib in both parental and PT-Res OVCAR8 cells, supporting its superior potency compared with that of SR4835 (Supplementary Fig. 4e and Supplementary Fig. 11g). The combination of CT7439 plus CDDP reduced the CDDP IC50 concentration to a level that is compatible with the plasma exposure typically reported in patients. Similar in vitro results were obtained with murine ID8 cells, in which CT7439 induced DNA damage and cell death as a single agent and improved the efficacy of CDDP but not that of a PARPi (Supplementary Fig. 11a and Supplementary Fig. 11h–i). Consistent with the results obtained with SR4835 (Supplementary Fig. 10a), p54^nrb^-KO cells were partially resistant to CT7439 and more sensitive to CDDP treatment (Supplementary Fig. 11j–k) than their parental counterparts were. At the molecular level, upon CT7439 treatment, SFPQ relocalized in perinucleolar condensates with newly transcribed RNA (Supplementary Fig. 11l), as previously observed with SR4835 (Fig. 6f). Overall, these data support the possibility that the two different CDK12is sensitize cells to CDDP through alterations of the same molecular pathway.

We next tested the efficacy of CT7439 alone or in combination with carboplatin (CDBCA) in vivo using ID8 p53^−/−^PTEN^−/−^ cells injected i.p. into syngeneic C57BL/6 J mice. Mice were randomized into four groups and administered vehicle, CT7439, CBDCA or a combination of CT7439 and CBDCA (Combo) and sacrificed after 20 days to evaluate metastatic dissemination (Supplementary Fig. 12a). As previously observed, single agents had modest effects on ascites formation and metastatic dissemination, whereas the combination treatment strongly reduced all the parameters considered, including the volume and cellularity of the ascites fluid, the number of macroscopic metastases and the tumor burden score (Fig. 7e–g and Supplementary Fig. 12b–c). The combination regimen completely abrogated metastatic dissemination to specific anatomical sites, such as the reproductive tract, diaphragm and peritoneal wall (Supplementary Fig. 12d–e). We then evaluated the ability of CT7439 alone or in combination with CDBCA to prolong the survival of tumor-bearing mice using humane endpoints (Supplementary Fig. 12f–g). In line with the results of the short-term experiments, compared with all the other treatment groups, the CT7439 + CBDCA-treated group presented reduced ascites cellularity and decreased total and district-specific metastatic spread (Fig. 7h–i and Supplementary Fig. 12h-k). Additionally, survival increased by approximately 30 days in the combination-treated group compared with that in the vehicle-treated group, which was double the survival period of the mice bearing tumors (Fig. 7h). Notably, compared with no treatment and PT treatment, 0.5 mg/kg CT7439 alone resulted in a prolonged survival benefit (Fig. 7h), with greater efficacy than SR4835 at 25 mg/kg (Fig. 3g). At necropsy, we observed extensive necrotic areas within the tumor deposits of combo-treated animals, indicating a therapeutic effect associated with long-term treatment outcomes (Fig. 7j and Supplementary Fig. 12l). Notably, since the tumor burden was defined by the presence of metastatic lesions regardless of necrosis, the measurements reported for the combination group in Fig. 7i likely overestimated the true extent of metastatic dissemination.

We next exploited a patient-derived xenograft (PDX) EOC model that we established from a PT-resistant HGSOC tumor.^39^ PDX#OV218.3 NSG mice were treated for three weeks and followed up for another 3 weeks (Fig. 7k). CT7439, both as a single agent and in combination with CBDCA, led to a significant reduction in the amount of ascites and in the number of tumor cells and spheroids isolated from the ascitic fluid (Supplementary Fig. 12m–p). Notably, the mice in the combination-treated group did not reach the survival endpoint and were sacrificed at the experimental endpoint for histological analyses. Kaplan-Meier survival curves revealed that compared with no treatment, CT7439 ± CBDCA increased the median survival rate (>56 days) (32 days) (Fig. 7l). Tumor dissemination to organs in the peritoneal cavity and the number of metastases were strongly reduced only in combination-treated mice (Fig. 7m–o and Supplementary Fig. 12q).

Taken together, these data provide strong support for the clinical evaluation of CDK12i and its combination with PT as a strategy to prevent or substantially delay the emergence of PT-resistant recurrences in EOC patients.

## Discussion

By combining in vitro and in vivo models with advanced molecular and systems biology approaches, we explored the possible significance and impact of CDK12/13 inhibition in hard-to-treat EOC.

Our work clearly demonstrated that CDK12is are particularly active in combination with PT (either CDDP or CDBCA) in vitro and in vivo but have minor effects when combined with PARPis or other chemotherapeutic agents (i.e., doxorubicin and taxol). This finding is in line with recent evidence showing that CDK12 inactivation does not improve the efficacy of the PARPi olaparib.^10^ The strong synergistic effect between PT and CDK12i was also reinforced by sequential pulse treatment with SR4835 and CDDP in different EOC models, which revealed diverse genetic alterations (see Supplementary Table 3), resulting in the inability of the cells to recover after medium-term treatment. These findings suggest that in PT-sensitive disease, combined treatment could be an effective regimen that might prevent the development of PT-resistant recurrence. Our results demonstrate that acquired resistance to PT and to CDK12/13 inhibitors is mutually exclusive, revealing a previously unrecognized therapeutic relationship that supports a paradigm shift in EOC treatment. We propose that combined treatment with CDK12i and CDDP may induce de novo cotranscriptional synthetic lethality through impairment of key DDR gene expression and RNA processing, thereby creating a vulnerability that is not restricted to a specific HR status. This possibility, which could merit experimental testing in dedicated clinical trials, is supported by in vivo data obtained using CT7439 and CBDCA alone or in combination in survival experiments. Both CT7439 alone and in combination with PT prolonged survival, but only the combination strongly reduced tumor burden, with the induction of large areas of necrosis in the few residual lesions. This evidence reinforces the possibility that the CDK12i+PT combination can prevent the regrowth of PT-resistant disease in vivo.

The lack of specificity, possible effects on the local microenvironment and toxic pharmacological profile are among the factors that have thus far limited the clinical development of CDK12is.^35^ These issues are overruled by CT7439, a more active and less toxic inhibitor currently tested as a single agent in a modular phase 1/2 clinical trial in patients with solid tumors, including EOC. The safety profile of CT7439 has been independently evaluated in GLP-compliant preclinical toxicology studies, which have been reviewed and approved as part of the FDA IND assessment. In these studies, the no-observed adverse effect level (NOAEL) was 1.0 mg/kg/day (both sexes). Although we did not perform a formal toxicity analysis of mice treated with CT7439 in combination with PT, we were able to treat them for up to 50 days without significant signs of toxicity or weight loss, suggesting that combined treatment could also have manageable side effects, a possibility we aim to confirm in an upcoming clinical trial dedicated to the EOC pathology we are currently designing.

In this context, it is worth mentioning that while the main curative effects of CDK12i were due to the induction of cancer cell death, the more pronounced and prolonged activity we observed in immunocompetent mice suggested that it might also have an impact on the host immune response. Accordingly, recent evidence suggests that CDK12/13 inhibition activates the STING pathway, promoting CD8 + T-cell infiltration and activation within tumors in prostate cancers.^77^ EOC usually presents as a cold immune-excluded or immune-desert tumor^78^; thus, determining whether the same pathway could be activated in this context and convert tumors into immune-infiltrated tumors, possibly allowing the use of immunotherapy, is particularly important. This is an aspect that we plan to explore in more detail in future work. We used TP53 + PTEN double-KO ID8 cells as a PT-Res murine model.^43^ Although the study of the specific role of TP53 or PTEN in the response to CDK12i was out of the scope of this work, recent reports suggest that CDK12 inhibition prevents prostate cancer development in PTEN-null but not in TP53-KO animals.^79^ It is therefore possible that alterations in the expression of PTEN play a role in determining the response to CDK12i, as also suggested by the association of PTEN loss with widespread alterations in transcriptional and RNA-processing programs in breast cancer.^80^ Overall, despite some limitations, our results strongly support the possibility that CDK12i could be a useful clinical strategy for the treatment of platinum-resistant ovarian cancer patients, possibly in combination treatments with PT.

To our knowledge, this is the first ever-generated model of CDK12i-resistant cells, providing a possible explanation for the synergistic effect of CDK12is with PT. CDK12i-resistant EOC cells become highly and selectively sensitive to platinum, suggesting that EOC cells may not be concomitantly resistant to both drugs. We did not observe major genomic aberrations in CDK12i-resistant models (see Supplementary Table 4), suggesting that the emergence of resistant clones is epigenetically regulated.

A possible molecular explanation for these biological phenotypes lies in the transcriptional reprogramming induced by both CDK12i and PT. We observed that both drugs increased the expression of short- and medium-length genes but decreased that of long-coding genes. Even if platinum can generate replication stress and transcriptional interference^,^^81^ thus participating in the regulation of long gene expression, these mechanisms cannot fully account for the observed length-dependent transcriptional suppression. Notably, we observed a similar effect upon CDK12 inhibition (SR4835), suggesting that this phenotype is not solely driven by genotoxic stress. Mechanistically, CDK12 is a well-established regulator of RNA polymerase II transcriptional elongation through phosphorylation of the CTD Ser2 residue, promoting productive elongation.^82^ Consistently, CDK12 loss preferentially affects long DNA damage response genes through premature transcription termination and reduced full-length transcript production,^83^ highlighting its central role in maintaining transcriptional processivity in long genes. Importantly, long genes are intrinsically vulnerable to transcription–replication conflicts because of the increased probability of collision between replication forks and the transcriptional machinery.^84^ In line with these findings, recent evidence has shown that CDK12/13 inhibition disrupts transcriptional elongation and impairs replication fork progression, linking transcriptional stress with replication-associated vulnerability.^61^ These mechanisms are not mutually exclusive and may converge on a shared transcription–replication vulnerability. Therefore, on the basis of our data and the literature, we propose a model in which impaired CDK12-dependent RNA polymerase II elongation and the intrinsic susceptibility of long-coding genes to transcription–replication conflicts represent key determinants of the observed length-dependent transcriptional suppression, potentially acting in concert with platinum-induced replication stress.

In contrast, neither CDK12i nor CDDP altered the expression of long noncoding RNAs. The reason for this difference in regulation is not completely clear, but it could rely on the lower stability of long noncoding RNAs with respect to long protein-coding RNAs. Alternatively, it is possible that the nascent or steady-state pre-long noncoding RNAs are less efficiently spliced than pre-mRNAs of protein-coding genes.^85,86^ Our results are in line with the notion that CDK12 is known to regulate transcriptional elongation, particularly for long and complex protein-coding genes involved in the DNA damage response.^18,19,58,60,61^ In contrast, noncoding RNAs display highly heterogeneous biogenesis and localization patterns^61,87^ and are not uniformly associated with nucleolar transcription. These findings represent possible molecular explanations for the observed differences in the regulation of long-coding versus noncoding genes. However, because noncoding RNAs are predominantly processed within the nucleus and bulk RNA preparation was used for RNA-seq experiments, we cannot exclude the possibility that technical bias affects expression and splicing analyses.

In PT-sensitive models, PT and especially CDK12i treatments specifically induce alterations in exon skipping and intron retention splicing in genes belonging to the Fanconi anemia (FA) pathway, which explains their acquired sensitivity to these drugs at the molecular level. More interestingly, this pathway was almost unaffected by splicing regulation in PT-resistant cells, whereas genes associated with both the FA and the base excision repair (BER) DNA repair pathways were significantly enriched in genes associated with skipping exons and intron-retention splicing alterations in CDK12i-resistant models. At a biological level, it is thus conceivable that the combined alteration in the splicing of FA and BER genes in CDK12i-resistant cells leads to synthetic lethality upon PT-induced DNA damage, ultimately explaining their hypersensitivity to the drug.

From a mechanistic point of view, we observed that CDK12 interacts with the splicing master regulator SFPQ/p54^nrb^ to ensure the proper elongation and splicing of long coding genes. Both SFPQ and CDK12 have previously been implicated in the regulation of long-coding genes,^33,34^ but the exact mechanism through which these genes function remains unclear. Our data suggest that the binding of CDK12 to SFPQ, through interaction with p54^nrb^, is necessary for the proper elongation and splicing of long-coding genes, indicating that these genes together regulate the cotranscriptional splicing cascade. In the absence of p54^nrb^, the impaired CDK12-SFPQ interaction results in nuclear retention of likely defective DDR transcripts upon release from CDK12i. We propose that p54^nrb^-structured domains may promote disorder-to-order transitions in physiological binding partners through a “folding-upon-binding” mechanism, potentially priming these proteins for subsequent functional interactions with additional partners, as suggested by others.^88,89^ This newly described pathway leads to global and coordinated regulation of alternative splicing and the expression of multiple long genes, making rescue experiments involving the use of selected exogenous constructs difficult. However, we acknowledge that since we have not used intronless constructions of DDR genes here, to prove the primary role of splicing in the observed phenotype, our hypothesis is not fully demonstrated, and this represents a possible limitation of our study that should be better addressed in the future.

Finally, it is important to highlight here that emerging evidence supports the possibility that the formation of membraneless condensates is a way for cells to enable rapid and finely tuned cellular responses to different stress signals.^90^ Our data suggest that the SFPQ/p54^nrb^ complex is a key determinant of nuclear condensate formation in response to CDK12i, which is likely to ensure the re-expression of long genes once the stress is resolved. This finding reinforces the physiological importance of condensate formation and RNA subcellular localization in response to cellular stresses.

Overall, our data offer further clarification of how transcriptional CDKs regulate RNA metabolism. Among these, CDK12/13 are necessary for the proper function of RNA polymerase and the splicing machinery, which, in turn, are necessary for the correct subcellular localization and splicing of nascent RNAs. The targeting of this interaction by the inhibition of CDK12/13 leads to cell death and could be used as a therapeutic approach in cancer, especially for tumors that are still difficult to treat. In this context, it is worth highlighting that CDK13 is an essential paralog of CDK12 and that the targeting of CDK13 is particularly effective in CDK12-inactive disease.^20^ Since CDK13 also has CDK12-independent functions in tumor progression,^11^ clarifying the specific roles of these two paralogs as therapeutic targets could provide relevant information for the design of specific and possibly less toxic anticancer drugs. Finally, toward a possible clinical translation of our findings in the context of precision oncology, it would be worth identifying possible biomarkers of CDK12/13i activity beyond CDK12 amplification. These types of translational studies will likely expedite the transfer of the promising platinum+CDK12i therapeutic combination to the clinic.

## Materials and methods

### Cell lines

The cell lines used in this study were sourced from multiple established biorepositories. OVCAR-8 and OVCAR-4 cells were obtained from the National Cancer Institute Developmental Therapeutics Program Tumor Repository, while the KURAMOCHI (JCRB0098) and OVSAHO (JCRB1046) cell lines were acquired from the Japanese Collection of Research Bioresources Cell Bank. The COV-362 (07071910), COV-318 (07071903), and OAW42 (85073102) cell lines were purchased from the European Collection of Authenticated Cell Cultures. Finally, OV-90 (CRL-11732), SKOV-3 (HTB-77), MDAH-2774 (CRL-10303), TOV-112D (CRL-11731), TOV-21G (CRL-11730) and NIH: OVCAR-3 (HTB-161) were from the American Type Culture Collection (ATCC). The breast cancer cell lines BT-474 (HTB-20), MCF-7 (HTB-22), MDA-MB-231 (HTB-26) and SK-BR-3 (HTB-30) were obtained from ATCC and maintained in Dulbecco’s modified Eagle’s medium (DMEM; Gibco, Cat# 61965) supplemented with 10% heat-inactivated fetal bovine serum (FBS; Gibco, Cat# A5256701) and 1% penicillin/streptomycin. Human embryonic kidney (HEK) 293FT cells (R70007; Invitrogen), which were used for lentivirus production, and 293 T/17 cells (CLR-11268; ATCC) were cultured in complete high-glucose DMEM. 12370 (RRID:CVCL_JY27) and 13699 (RRID:CVCL_JY29) cell lines were kindly provided by Dan Cacsire Castillo-Tong (Medical University of Vienna, Austria)^91^ and maintained in DMEM/Ham’s F12 (1:1) high-glucose complete medium. Cisplatin-resistant (PT-Res) EOC cells were generated as previously described by Sonego et al.^41^ In brief, parental EOC cells were exposed to a 10-fold higher dose of cisplatin (CDDP) than the calculated half-maximal inhibitory concentration (IC50) for 2 h (TOV-112D, OVSAHO) or by an incremental method (TOV-21G, OVCAR-8), followed by recovery in drug-free complete medium. In total, PT-res cells received 20 pulse treatments. The cells were then kept in CDDP-free medium. SR4835-resistant (SR-Res) isogenic EOC cells were established using the same approach. Parental cells were treated for 72 h with SR4835 (Selleckchem, Cat# S8894), allowed to recover in drug-free complete medium until they reached 70–80% confluence and were reexposed to the drug. This pulse–recovery cycle was repeated 20 times, and the drug dose was increased every fifth treatment to select for stable resistance to SR4835. Resistant cells were subsequently maintained in drug-free medium, and resistance was periodically validated. All the established cell lines were maintained in RPMI-1640 medium (Gibco, Cat# 61870) supplemented with 10% heat-inactivated fetal bovine serum (FBS, Gibco, Cat# A5256701) and 1% penicillin/streptomycin. COV-318 cells were cultured in complete DMEM. ID8 P53^−/−^, ID8 P53^−/−^ BRCA1^−/−^, ID8 P53^−/−^ BRCA2^−/−^, and ID8 P53^−/−^ PTEN^−/−^ murine cells were kindly provided by Iain McNeish (Hammersmith Hospital, London)^42,43^ and grown in complete high-glucose medium supplemented with ITS [insulin (5 μg/ml), transferrin (5 μg/ml), and sodium selenite (5 ng/ml)].

All the cell lines were grown under standard conditions at 37 °C and 5% CO_2_ and were routinely authenticated using the CLA IdentiFiler Plus PCR Amplification Kit (Thermo Fisher, Cat# A65672) to identify short tandem repeat profiles. All the cell lines were regularly screened for Mycoplasma contamination using a MycoAlert kit (Lonza Bioscience, Cat# LT07-318).

### Mouse models

All animal procedures were reviewed and approved by the CRO Institutional Organism for Animal Wellbeing and the Italian Ministry of Health (aut. no. 753/2021-PR to G.B.; aut. no. 333/2023-PR to G.B.). Female C57BL/6 J (strain code: 632), CD-1 Nude (strain code: 086) or NOD scid gamma (NSG, strain code: 614) mice (3–4 weeks old) were obtained from Charles River Laboratories and housed in the CRO-Aviano animal facility under standard and controlled environmental conditions (up to 5 mice per cage, 22 °C with 40–60% humidity) and pathogen-free conditions. Animals were health-monitored daily in accordance with EU Directive 2010/63 and randomly assigned to experimental groups. The mice were sacrificed in accordance with the ethical endpoint.

### Ovarian cancer patient-derived samples and models

Biospecimens were obtained from patients who gave their informed consent under Internal Review Board approval CRO-IRB #05-2014 and protocols approved on 7 October 2019 by the Ethics Committee (OutCoME protocol, CRO-2019-53 approval CEUR 2019-Sper-084). Patient-derived primary cells were obtained from the ascites of patients with EOC collected at Centro di Riferimento Oncologico of Aviano. Samples were collected from EOC patients representing different ages, histotypes, disease stages and responses to therapies, as reported in Supplementary Table 2. Primary tumor cells were isolated by centrifuging ascitic fluids at 1200 rpm for 10 min, after which red blood cells were eliminated by treating the cell pellet with BD Bioscience Lysing Buffer (Cat# 555899) for 5 min at 37 °C and centrifuging them again at 1200 rpm for 5 min. The primary cells were then maintained in OCMI medium (M199 medium + F12-HAM medium supplemented with insulin (20 μg/ml), hydrocortisone (500 ng/ml), epidermal growth factor (EGF) (10 ng/μl), 2% fetal bovine serum (FBS), 1% penicillin/streptomycin, cholera toxin (25 ng/ml), and 2% ascitic liquid) at 37 °C in 5% CO_2_. To maintain consistent cellular properties, cells were used at no more than five passages for all experiments.

### Compounds and drug treatments

EOC cells were seeded in 96-well plates and, after 24 h, exposed to increasing concentrations of the indicated compounds. CDK12/13 inhibition was achieved using SR4835 (Selleckchem, Cat# S8894), THZ531 (Selleckchem, Cat# S6595) or CT7439, provided by Carrick Therapeutics, Dublin. Cells were also treated with chemotherapeutic agents, such as cisplatin (CDDP), doxorubicin and taxol, obtained from the Internal CRO Unit of Antiblastic Drugs, or niraparib (Selleckchem, Cat# S2741). When CDK12i was combined with other drugs, the cells were pretreated with or without CDK12i for 16 h, followed by 48 h with CDDP, niraparib, or doxorubicin. Cell viability was quantified using the CellTiter 96 AQueous MTS assay (Promega, Cat# G358C) according to the manufacturer’s instructions. The absorbance was measured using a microplate reader (Infinite® M1000 Pro, Tecan). The cell viability is expressed as a percentage (mean ± SD) relative to that of the untreated controls, and the IC50 values were obtained by a nonlinear regression curve of the log(concentration) versus the response of three (*n* = 3) independent experiments using GraphPad Prism (version 10.6.1).

### Synergy measurements

Drug interactions between CDK12 inhibitors and cisplatin or niraparib were evaluated using the highest single agent (HSA) model implemented in the SynergyFinder 3.0 platform^92^ (https://synergyfinder.aittokallio.group/). To estimate the outlier measurements, the cNMF algorithm^93^ implemented in SynergyFinder was used. An HSA score >10 indicates synergy, a score between −10 and +10 indicates additivity, and a score < -10 indicates antagonism. Cells were seeded in 96-well plates and treated for 72 h with SR4835 or CT7439 in combination with cisplatin or niraparib at equipotent ratios (IC10:10, IC25:25, and IC50:50) or nonequipotent ratios (IC10:25, IC10:50, and IC25:50). Cell viability was then assessed by the MTS assay as described above.

### Vectors, transfections, and viruses

Lentiviral particles were produced in HEK293FT cells by standard calcium phosphate cotransfection of the lentiviral-based shRNA construct (pLKO) with the packaging vectors pGAG/POL and pVSV-G (Invitrogen). Seventy-two hours post-transfection, the viral-containing medium was collected and used to transduce the EOC cell lines. shRNA vectors targeting p54^nrb^ (TRCN0000286628), CDK12 (TRCN0000001795), and CDK13 (TRCN0000000704) and a nontargeting control (SHC002) were purchased from Sigma‒Aldrich. Stable knockdown cells were selected by supplementation of the medium with puromycin (0.5 μg/ml; Cat# P9620; Sigma‒Aldrich). Human pcDNA3 Myc-Tagged SFPQ-WT, Myc-SFPQ-ΔN, and Myc-SFPQ-ΔRRM1 + 2 were obtained from Dr. Benjamin Blencowe through the Addgene Inc. consortium (Cambridge, Massachusetts) and described by Rosonina et al.^94^ (Cat# 35183, #35184 and #35267, respectively; Addgene). The pCI-Neo-His-Pitaire-Myc plasmid was obtained from Dr. Jonathon Pines through the Addgene consortium and described by Ko et al.^95^ (Cat# 26066, Addgene). The human V5-Tagged CDK12-WT, V5-CDK12-KDΔ, V5-CDK12-RSΔ and V5-CDK12-PRMΔ plasmids were obtained from Dr. Alan Ashworth and described by Chou et al.^65^ The plasmids were transfected into HEK293T/17 or OVCAR-8 cells using FuGENE HD Transfection Reagent (Cat# E2311; Promega) according to the manufacturer’s instructions.

### Colony formation assay

Cells were seeded at a density of 500–1000 cells per well in 6-well plates and allowed to adhere overnight. Cells were treated for 72 h with THZ531 or SR4835, followed by drug washout. In combination experiments, cells were pretreated with SR4835 or CT7439 for 16 h, followed by 3 h of treatment with cisplatin (CDDP). After treatment, the cells were released in drug-free complete medium for 10–14 days, and the medium was changed every 3–4 days. Colonies were fixed and stained with 0.5% crystal violet in 20% methanol for 20 min, and excess stain was removed by repeated washes with distilled water. Colonies were quantified using Fiji-ImageJ software (v1.54p, NIH), and colony numbers were normalized to those of the untreated control and expressed as the mean percentage of three independent biological replicates.

### Comparison of resistance onset ± CDK12i

In accordance with the PT-Res generation protocol previously described by Sonego et al.,^41^ parental EOC cells at 70–80% confluence were pretreated or not for 16 h with SR4835 (OVSAHO and OVCAR8: 100 nM; COV362: 200 nM), followed by exposure to CDDP following the same schedule previously reported by Sonego et al.^41^ After each treatment, the cells were allowed to recover in drug-free medium until they reached 70–80% confluence. COV362 and OVCAR8 cells underwent dose doubling every 5 pulses (incremental approach), whereas the OVSAHO dose was doubled after pulse 10 and again after pulse 15 (pulse approach), for a total of 20 treatments.

### Generation of p54^nrb^-KO clones

CRISPR/Cas9 technology^96^ was used to generate p54^nrb^-knockout OVCAR8 PT-Res#8 cells. Cells were first transduced with Cas9-expressing lentiviral particles (Cat#LVCAS9BST**;** Sigma) and selected with blasticidin (3 µg/ml; Cat# 15205; Sigma‒Aldrich). The OVCAR8 PT-Res#8 Cas9 pool cells were then transduced with a lentiviral guide RNA targeting p54^nrb^ (Sigma; HSPD0000028466; gRNA: 5′-TGGACAATATGCCACTCCG-3′; PAM: TGG; RefSeq NM_001145408, exon 6), followed by antibiotic selection (blasticidin, 3 µg/ml, and puromycin, 0.5 μg/ml; Cat# P9620; Sigma‒Aldrich). Single-cell-derived clones were isolated and expanded, and p54^nrb^ protein loss was confirmed by immunoblotting. Genomic DNA was extracted with the Maxwell 16 Tissue DNA Purification Kit (Promega, Cat# AS1030) according to the manufacturer’s protocol and quantified using a Quantus Fluorometer (Promega, Cat# E6150) and the QuantiFluor® ONE dsDNA System (Promega, Cat# E4870). Isolated genomic DNA was then subjected to targeted sequencing on a MiSeq platform (Illumina) to verify editing events and determine allelic composition. Only clones exhibiting complete (100%) allelic disruption were used for downstream experiments.

### CDK targeted next generation sequencing

Frozen ovarian cancer tissues were maintained at -80 °C until genomic DNA extraction. DNA was isolated using the Maxwell 16 Tissue DNA Purification Kit (Promega, Cat# AS1030) according to the manufacturer’s protocol and quantified with a Quantus Fluorometer (Promega, Cat# E6150) using the QuantiFluor® ONE dsDNA System (Promega, Cat# E4870). For targeted sequencing of CDKs, an AmpliSeq Custom Panel (Illumina, Cat# 20019102) was designed to target the coding sequence of all 20 CDKs. Twenty nanograms of genomic DNA per sample was used, according to the manufacturer’s instructions. Library quality and concentration were assessed with a Quantus fluorometer (Promega, Cat# E6150) before sequencing. Libraries were sequenced on an Illumina MiSeq (Illumina, Cat# SY-410-1003) platform with paired-end 250 bp reads, enabling high coverage (>1000) and accurate variant calling. Local Run Manager, Variant Studio (Illumina) and Integrative Genome Viewer^97,98^ (IGV, v2.6.1; UC San Diego and the Broad Institute of MIT and Harvard, https://igv.org) were used to analyze the NGS output files after the quality control (QC) of the reads. Variants with potential therapeutic relevance are reported only when the VAF is ≥ 5%. The Varsome^99^ (https://varsome.com) and Franklin by Genoox (https://franklin.genoox.com) databases were used to explore the clinical relevance of the called variants. The functional impact of variants of uncertain significance (VUS) was assessed using AlphaMissense,^100^ and the pathogenicity scores and classifications were determined. Lollipop plots were generated in cBioPortal (https://www.cbioportal.org) by integrating mutation and variant positions with cBioPortal annotation.

### Droplet digital PCR (ddPCR) of CDK12/13

For copy number alterations (CNAs) of CDK12 and CDK13, 10 ng of genomic DNA (see ‘CDKs NGS targeted sequencing’ paragraph) per sample was analyzed using droplet digital PCR (ddPCR) on the QX200 Droplet Digital PCR System (Bio-Rad, Cat# 1864001). We included probes for CDK12 (https://www.bio-rad.com/digital-assays/assay-detail/dHsaCNS941515482) and CDK13 (https://www.bio-rad.com/digital-assays/assay-detail/dHsaCNS546990613) and a PrimePCR ddPCR Copy Number Assay (Bio-Rad) using WSB1 (https://www.bio-rad.com/digital-assays/assay-detail/dHsaCP2506685) and VOPP1 (https://www.bio-rad.com/digital-assays/assay-detail/dHsaCP2506684) as references, respectively. Ten microliters (10 ng) of genomic DNA was added to 10 μl of ddPCR Supermix for Probes (no dUTP) (Bio-Rad, Cat# 1863024) and to 2 μl of the primer and probe mixture according to the manufacturer’s protocol. Droplets were generated using the QX™ Droplet Generator (Bio-Rad, Cat# 1864002), where the reaction mixture was added together with Droplet Generation Oil for Probes (Bio-Rad, Cat# 1863005). Droplets were then transferred to a 96-well plate and thermally cycled under the following conditions: 10 min at 95 °C, 40 cycles of 94 °C for 30 s, and 58 °C for 1 minute, followed by 98 °C for 10 min (the ramp rate was 2.5 °C/s). Droplets were analyzed using a QX200 Droplet Reader (Bio-Rad, Cat# 1864003) for fluorescence measurement of the FAM and HEX probes. The ddPCR data were analyzed with QX Manager Software (Bio-Rad, v2.0). Gating thresholds for FAM-positive droplets were defined using no-template and HEX-positive controls. CDK12 (17q12) was normalized to the intrachromosomal reference WSB1 (17q11.2), whereas the CDK13 (7p14.1) ratio was determined by normalization to VOPP1 (7p11.2). The final copy number (CN) values were obtained from target/reference ratios, with amplification and deletion defined as CN > 2.5 and CN < 1.5, respectively.

### Genomic profiling of EOC cell lines

Genomic profiling of parental and SR4835-resistant isogenic EOC cells was performed using the AVENIO Tumor Tissue Comprehensive Genomic Profiling (CGP) 2.0 panel (Roche, incorporating Foundation Medicine technology). This assay employs a hybrid capture/amplicon‑based next‑generation sequencing approach to detect single-nucleotide variants (SNVs), insertions and deletions (InDels), structural rearrangements, and copy number alterations (CNAs) across a targeted set of 335 cancer‑related genes. In addition to individual variant calls, the panel enables the identification of complex genomic signatures, including tumor mutational burden (TMB), microsatellite instability (MSI), loss of heterozygosity (LOH), and homologous recombination deficiency (HRD) status. Briefly, genomic DNA was extracted using the Maxwell 16 Tissue DNA Purification Kit (Promega, Cat# AS1030) according to the manufacturer’s protocol and quantified with a Quantus Fluorometer (Promega, Cat# E6150) using the QuantiFluor® ONE dsDNA System (Promega, Cat# E4870). For library preparation, 50 ng of DNA per sample was enzymatically fragmented, end-repaired, and ligated to Illumina-compatible adapters supplied in the AVENIO Tumor Tissue CGP V 2.0 Kit, as specified in the manufacturer’s protocol. Libraries were amplified, purified, and subjected to hybrid capture enrichment. After postcapture amplification and purification, the size distribution and concentration of the libraries were assessed using an Agilent 2200 TapeStation with D1000 ScreenTape (Agilent Technologies, Cat# 5067-5582). Sequencing-ready libraries were normalized, pooled, denatured and loaded onto an Illumina NextSeq 500/550 platform (Illumina, Cat# 20024908). Sequencing data were loaded and processed by the AVENIO analysis pipeline in AVENIO Connect Software (Roche, Basel). For the analyses, the following cutoffs were applied: MSI-high >0.0124, LOH-positive ≥0.16, TMB-high >10 mutations/Mb, and HRD-positive ≥0.7.

### Analysis of CDK12/13 prognostic value

Patient samples from our cohort, as reported in Supplementary Table 2, were collected between 2008 and 2021 (2008-01-21 to 2021-10-19). Survival analyses (overall survival [OS] and progression-free survival [PFS]) were restricted to patients with stage III-IV EOC. The time of origin for survival analyses was defined as the date of primary surgery or initiation of neoadjuvant chemotherapy, as the date of diagnosis was not consistently available. The loss-of-function (LoF) category included all copy number losses (defined as <1.5 copies; ratio <0.75) and pathogenic mutations (2/12 for CDK12 and 1/18 for CDK13). The gain-of-function (GoF) category included copy-number amplifications (defined as >2.5 copies; ratio >1.25). Kaplan‒Meier curves for PFS and OS were generated, and group differences were evaluated using the log-rank test in GraphPad Prism v10.0.

Transcriptomic expression data for CDK12 and CDK13, along with clinical information for ovarian serous cystadenocarcinoma (TCGA-OV), were retrieved from the Human Protein Atlas database (https://www.proteinatlas.org/). The cohorts included in the OS analyses consisted of CDK12 (*n* = 350) and CDK13 (*n* = 349). Proteomic data for the TCGA-OV cohort were obtained from the GDAC Firehose repository (Broad Institute; formerly TCGA Provisional) and accessed via the cBioPortal for Cancer Genomics (https://www.cbioportal.org). Mass spectrometry-based protein expression data for CDK12 and CDK13 were extracted, along with matching clinical annotations, OS and disease-free survival (DFS). The initial cohort included 174 patients. Individuals lacking complete protein expression or disease-free survival (DFS)/overall survival (OS) data were excluded, resulting in subsets with full information for survival analyses: CDK12 DFS (*n* = 147), CDK12 OS (*n* = 167), CDK13 DFS (*n* = 149), and CDK13 OS (*n* = 169).

No additional interset normalization was applied, and expression values were used as reported. Patients were divided into high- and low-expression groups on the basis of the optimal expression cutoff determined by maximally selected rank statistics (“best cutoff”) using the *maxstat* R package (v0.7-26). The cutoff values corresponded to the protein expression level that maximized the separation between survival curves. Kaplan‒Meier survival curves were generated for OS and DFS, and differences between groups were assessed using the log-rank test. Univariate hazard ratios (HRs) and 95% confidence intervals (CIs) were estimated using Cox proportional hazards regression. Analyses were performed in R (v4.4.2) with RStudio (2024.12.1.563) using the following packages: *survival* v3.8-3, *survminer* v0.5.1, *dplyr* v1.1.4, *tidyverse* v2.0.0, and *ggplot2* v3.5.2.

### ROC plot analysis

Receiver operating characteristic (ROC) analysis was performed using the ROC plotter platform^101^ (https://rocplot.com/ovarian/index) to evaluate the predictive value of CDK12 and CDK13 expression in ovarian cancer. Relapse-free survival (RFS) at 6 months was selected as the clinical endpoint (*n* = 1347). The dataset was filtered for patients treated with platinum-based chemotherapy, resulting in a cohort of 1209 patients, including 1095 responders and 114 nonresponders. Gene expression data were queried using the probe sets 219226_AT (CDK12) and 207318_s_at (CDK13). Expression levels were compared between responders and nonresponders, and boxplots were generated. In addition, ROC curves were generated to assess the area under the curve (AUC) of CDK12 and CDK13.

### CDK12 and RBP correlation analysis

Transcriptomic data for high-grade serous ovarian cancer (TCGA, GDC) were accessed via the cBioPortal for Cancer Genomics (https://www.cbioportal.org). mRNA expression (transcripts per million, TPM) for CDK12 was used as the query to compute Spearman’s correlation coefficients, *p* values, and FDR-adjusted *q* values. The analysis included 427 samples, and no additional normalization was applied beyond the reported values. Genes were filtered for RNA-binding functions using a comprehensive repertoire of 1555 RNA-binding proteins (RBPs) as annotated by Hentze et al.^62^ and splicing-related RNA gene sets obtained from MSigDB (*Homo sapiens*) using MSigdbr (v 25.1.1), including Gene Ontology, KEGG, Reactome, and other curated pathways, and then were ranked by correlation coefficient. Waterfall plots were generated in R (v4.4.2) with RStudio (2024.12.1.563) using tidyverse (v2.0.0) and ggplot2 (v3.5.2), and the results are displayed as Spearman correlation coefficients as a function of gene rank. Genes were color-coded according to functional annotation as RNA-binding proteins (*n* = 1064), splicing factors (*n* = 420), or both (*n* = 271).

### Immunofluorescence

Cells were seeded on glass coverslips and treated with CDK12/13 inhibitors (SR4835, THZ531, and CT7439) or CDDP, as indicated. For γ-H2AX and RAD51 staining, the cells were first fixed in 4% paraformaldehyde (PFA) for 20 min, permeabilized with 0.5% Triton X-100 for 10 min and blocked in 1% bovine serum albumin (BSA; Sigma‒Aldrich) for 1 h. For CDK12, SFPQ and p54^nrb^ staining, the cells were first permeabilized with CSK buffer (10 mM PIPES (pH 7.0), 100 mM NaCl, 300 mM sucrose, and 3 mM MgCl2 containing 0.5% Triton X-100) for 3 min on ice, fixed in 4% PFA for 20 min and blocked in 3% BSA for 1 hour. For tubulin and acetylated tubulin staining, the cells were first fixed in 4% PFA for 20 min, permeabilized with 0.2% Triton X-100 for 5 min and blocked in 1% BSA for 1 hour. Slides were incubated overnight at 4 °C in a humid chamber with the following primary antibodies in blocking buffer: anti-γ-H2AX (Millipore, 1:200; Cat# 05-636; RRID: AB_309864), anti-RAD51 (Abcam, 1:100; Cat# ab133534; RRID: AB_2722613), anti-SFPQ (Sigma, 1:150; Cat# AV40572; RRID: AB_1856775), anti-SFPQ (1:100; Sigma‒Aldrich; Cat# WH0006421M2; RRID: AB_1843565), anti-p54^nrb^ (Bethyl, 1:200; Cat# A300-582A; RRID: AB_495505), anti-CDK12 (Novus Biologicals, 1:100; Cat# H00051755-M04; RRID: AB_828766), anti-Tubulin FITC (Sigma, 1:200; Cat# F1268; RRID: AB_476967) or anti-acetylated tubulin (Sigma, 1:200; Cat# T6793; RRID: AB_4785). Alexa Fluor-conjugated secondary antibodies (1:250) and nuclei stained with TO-PRO-3 iodide (Invitrogen, 1:1000; Cat# T3605) were incubated for 1 h at room temperature, after which the coverslips were placed on a glass holder with Mowiol mounting medium supplemented with 2.5% DABCO antifade adjuvant. Confocal images were acquired through a TCS-SP8 confocal system (Leica Microsystems) interfaced with Leica confocal software (v3.5.5.19976) or Leica Application Suite X software (v6.1.1). The images were processed using Fiji-ImageJ software (v1.54p; NIH). γ-H2AX foci were counted using the “Analyze particle” built-in plugin on thresholded images and reported as the number of spots per nucleus, or γ-H2AX integrated density was quantified as the product of the nuclear area and its mean gray value (integrated density=area mean gray value). For immunofluorescence analyses, at least 50–100 nuclei per condition were quantified across 3 independent experiments. Quantification was performed on multiple randomly selected fields using consistent acquisition and analysis parameters.

### Nascent RNA immunofluorescence

Cells were treated with SR4835 or CT7439 for 6 h, as indicated, and incubated with 1 mM 5-ethynyl uridine (EU, Thermo Fisher, Cat# E10345) for 1 h at 37 °C to label nascent RNA. For RNA polymerase I inhibition controls, cells were treated with 50 ng/mL actinomycin D (Sigma, Cat# A9415) for 1 h or 500 nM CX-5461 (Selleck Chemicals, Cat# S2684) for 3 h. Following EU incorporation, cells were fixed in 4% PFA for 15 min at room temperature and permeabilized with 0.5% Triton X-100 in PBS for 5 min. Labeling of the EU was performed using the Click-iT RNA Alexa Fluor 488 Imaging Kit (Thermo Fisher, Cat# C10329) according to the manufacturer’s protocol. Briefly, the click reaction was achieved with Alexa Fluor 488 azide in reaction buffer for 30 min at room temperature in the dark. The cells were then blocked in 3% BSA in PBS for 1 h and incubated overnight at 4 °C with the following primary antibodies: anti-SFPQ (Sigma, 1:150; Cat# AV40572; RRID: AB_1856775), anti-p54^nrb^ (Bethyl, 1:200; Cat# A300-582A; RRID: AB_495505) or anti-CDK12 (Novus Biologicals, 1:100; Cat# H00051755-M04; RRID: AB_828766). Alexa Fluor secondary antibodies (1:250) and nuclear counterstaining with TO-PRO-3 iodide (Invitrogen, 1:1000; Cat# T3605) were performed by incubation for 1 h at room temperature, after which the coverslips were mounted in Mowiol with 2.5% DABCO. Confocal images were acquired as described in the Immunofluorescence section. Nascent RNA signals were assessed according to an adapted version of the STAR protocol from Martin et al.^73^ Briefly, the distribution of nascent RNA and SFPQ protein signals within the nucleus across different conditions was determined by calculating their coefficients of variation as follows. Single nuclei were identified with Fiji-ImageJ software (version 1.54p; NIH) using the macro provided with the aforementioned protocol, with minor adaptations. Afterward, images from approximately 100 cells per condition were loaded into MATLAB (R2024b Update 5, v24.2.0.2863752) to calculate the coefficient of variation using the provided script. Integrated density was quantified in Fiji (ImageJ) as the product of the nuclear area and its mean gray value (integrated density = area mean gray value).

### Proximity ligation assay (PLA)

PLA was performed using a Duolink in situ kit (Sigma‒Aldrich, Cat# DUO94102) following the manufacturer’s instructions. Briefly, cells grown on glass coverslips were treated as indicated in the figure legends, fixed in 4% PFA for 15 min at room temperature, permeabilized with 0.5% Triton X-100 and 0.1% sodium citrate for 5 min, and blocked with Duolink blocking solution (Sigma‒Aldrich, Cat# DUO82007). Primary antibodies were incubated overnight at 4 °C in the following antibody diluences: anti-SFPQ (Sigma, 1:150; Cat# AV40572; RRID: AB_1856775), anti-p54^nrb^ (Bethyl, 1:200; Cat# A300-582A; RRID: AB_495505), anti-rRNA (Santa Cruz Biotechnology, 1:100; Cat# sc-33678; RRID: AB_628226) or anti-CDK12 (Novus Biologicals, 1:100; Cat# H00051755-M04; RRID: AB_828766). For the negative control, only one PLA probe was used. PLUS (Sigma‒Aldrich, Cat# DUO92002) and MINUS (Sigma‒Aldrich, Cat# DUO92004) PLA probes were applied for 1 h at 37 °C, followed by ligation and rolling-circle amplification (Sigma‒Aldrich, Cat# DUO92014) at 37 °C for 30 min and 100 min, respectively. Washes were performed with Duolink wash buffers A and B (Sigma‒Aldrich, Cat# DUO82049). The nuclei were counterstained with TO-PRO-3 iodide (Invitrogen, Cat# T3605; 1:500 in 0.01% wash buffer B) for 30 min, and the coverslips were mounted with Duolink In Situ Mounting Medium (Sigma‒Aldrich, Cat# DUO82040). Confocal images were acquired as described in the Immunofluorescence section. For the PLA analyses, at least 50–100 nuclei per condition were quantified across 3 independent experiments. Quantification was performed on multiple randomly selected fields using consistent acquisition and analysis parameters. PLA punctuated signals were assessed in Fiji-ImageJ software (version 1.54p; NIH) using the built-in “Find Maxima” plugin and reported as the number of spots per nucleus. The PLA signal was quantified by counting fluorescent spots within nuclei using ImageJ and normalizing to the total number of nuclei per field. With respect to the SFPQ-rRNA PLA, the PLA spots were detected using the threshold method with Fiji, and the x and y coordinates were gathered using the built-in function. K-nearest neighbor analysis (k-nn, with *k* = 4) was performed in R (v4.4.2) with RStudio (2024.12.1.563) using the *tidyverse* v2.0.0 and *RANN* v2.6.2 packages, and the calculated coefficient of variation (CV) was plotted in GraphPad Prism v10.0.

### RNAscope® in situ hybridization

Cells were seeded onto glass coverslips coated with 0.01% poly-L-lysine (Sigma, Cat# P8920) following the manufacturer’s instructions and allowed to adhere overnight. The cells were subsequently treated with 300 nM SR4835 for 1, 3, or 6 h. For the chase experiments, the cells were released into drug-free medium for 24 h. After treatment, the cells were fixed with 4% paraformaldehyde for 30 min at room temperature, dehydrated in a graded series of ethanol (50%, 70%, and 100%, 1 min each), and stored at −20 °C. Before hybridization, the coverslips were rehydrated through a descending ethanol series (100%, 70% and 50%, 1 min each) and permeabilized with 0.1% Tween 20 in PBS for 10 min. Subsequent steps were performed according to the RNAscope™ Multiplex Fluorescent Reagent Kit v2 protocol (ACD Biotechne, Cat# 323100). Endogenous peroxidase activity was then blocked using RNAscope peroxidase reagent (ACD Biotechne, Cat# 322381) for 10 min at room temperature, followed by incubation with RNAscope Protease III (1:15 in PBS, ACD Biotechne, Cat# 322381) for 10 min at room temperature. Probes hybridization was achieved using RNAscope probe mix targeting ATM (ACD Biotechne, Cat# 419271) and RAD51 (diluted 1:50 in ATM probe, ACD Biotechne, Cat# 459031-C2) at 40 °C for 2 h in a HybEZ™ II Oven (ACD Biotechne, Cat# 241000ACD). Both positive (ACD Biotechne, Cat# 320861) and negative (ACD Biotechne, Cat# 320871) control probes were included in the assay. Signal amplification was achieved by sequentially applying RNAscope Multiplex FL v2 (ACD Biotechne, Cat# 323100) Amp 1 (30 min), Amp 2 (30 min), and Amp 3 (15 min) at 40 °C, with two washes in 1X RNAscope Wash Buffer (ACD Biotechne, Cat# 310091) between each step. Each channel was independently developed as follows: HRP incubation (ACD Biotechne, Cat# 323100) for 15 min at 40 °C, followed by channel-specific Opal dye (Akoya Biosciences) deposition for 30 min at 40 °C, and a subsequent HRP blocker (ACD Biotechne, Cat# 323100) incubation for 15 min at 40 °C. Opal 520 (1:1000, Akoya Biosciences, Cat# OP-001001 in 1X Plus Amplification Buffer, Akoya Biosciences, Cat# FP1498) was assigned to ATM-C1, and Opal 620 (1:1000, Akoya Biosciences, Cat# OP-001004 in 1X Plus Amplification Buffer, Akoya Biosciences, Cat# FP1498) was assigned to RAD51-C2. Nuclei were counterstained with DAPI (RNAscope 4–6-diamino-2-phenylindole, ACD Biotechne, Cat# 323100), and slides were mounted in Mowiol containing 2.5% DABCO. Images were acquired using a Leica TCS-SP8 confocal system or Leica Application Suite software (version 6.1.1). The ATM and RAD51 punctate signals were quantified using Fiji-ImageJ (v1.54p; NIH) with the built-in “Find Maxima” plugin. For quantification analyses, at least 50–100 nuclei per condition were quantified across 3 independent experiments. Quantification was performed on multiple randomly selected fields using consistent acquisition and analysis parameters. Spots in the nuclei and cytoplasm were counted and normalized to the total number of cells per field. The nuclear‒cytoplasmic ratio of the normalized spots was calculated and plotted using GraphPad Prism v10.0.

### In vivo experiments

C57BL/6 and NU/NU CD-1 nude mice were intraperitoneally injected with 2×10^6^ ID8 P53^−/−^ PTEN^−/−^ cells, and NSG mice were injected with 2×10^6^ patient-derived xenograft cells (PDX#OV218.3, this paper). Following engraftment, the mice were randomly assigned to four groups (*n* = 5 or 8 per group) and treated three times per week on alternate days with vehicle (0.9% NaCl), SR4835 (25 mg/kg, Selleckchem, Cat# S8894) or CT7439 (0.5 mg/kg, Carrick Therapeutics, Dublin), administered alone or in combination with carboplatin (CBDCA, 7.5 mg/kg in combination with SR4835 or 5 mg/kg with CT7439), according to the schedule indicated in the figures. In NSG mice, CT7439 ± CDDP treatments were administered for three weeks and suspended for another three weeks, during which time the combination-treated mice received supportive subcutaneous hydration (500 µL 0.9% NaCl + 500 µL 5% glucose) on alternate days. The mice were euthanized on the basis of ascites formation or at humane endpoints. Kaplan‒Meier survival curves were generated, and the significance was evaluated with the log-rank (Mantel‒Cox) test. Ascitic fluid and abdominal organs were collected for metastatic analysis. The tissues were fixed in formalin, dehydrated in ethanol, embedded in paraffin, and sectioned for histological evaluation. Sections were stained with hematoxylin and eosin (H&E) and examined using a light microscope in a blinded manner. Metastatic lesions were categorized by size as small (S, ≤20 cells), medium (M, >20 cells and <2 mm), or large (L, >2 mm). A metastatic score for each organ was calculated by summing weighted counts, assigning 1 to S, 2 to M, and 4 to L lesions. Differences between groups were evaluated using one-way ANOVA with Tukey’s post hoc test for multiple comparisons in GraphPad Prism v10.0. Formalin-fixed, paraffin-embedded (FFPE) sections from C57BL/6 mice treated with SR4835 ± CBDCA for 20 days were used for immunohistochemistry. After deparaffinization and epitope retrieval (40 min at 96 °C; Dewax and HIER Buffer H; Thermo Scientific; Cat# TA-999-DHBH), endogenous peroxidase activity was blocked with 3% H_2_O_2_ for 10 min. The sections were incubated with 10% goat serum in PBS for 30 min at room temperature, followed by incubation with a primary antibody for 90 min at room temperature against γ-H2AX (Cell Signaling; 1:2000; Cat# 7631; RRID:AB_10860771). Secondary biotinylated antibodies were applied using the VECTASTAIN Elite ABC Kit (Vector Laboratories, Cat# VC-PK-6100-KI01), and immunoreactivity was visualized with DAB substrate (Vector Laboratories, Cat# VC-SK-4100-KI01) and counterstained with hematoxylin. Slides were scanned using a NanoZoomer S60 Digital Slide Scanner (Hamamatsu) and visualized with NDP.view2 software.

### RNA-seq analysis

RNA sequencing was performed on OVCAR-8, COV-362, OVSAHO, and TOV-112D ovarian cancer cell lines, including parental lines, PT-Res and SR-Res isogenic derivatives, and primary OV206 and OV213 cells (Supplementary Fig. 7a). Cells were treated with SR4835 (0.3 µM, 6 h; Selleckchem, Cat# S8894) or cisplatin (CDDP, 8 h; 20–35 µM depending on the cell line). Total RNA was extracted using TRIzol reagent (Invitrogen, Cat# 15596026) according to the manufacturer’s protocol and quantified by reading in NanoDrop (Thermo Fisher) and assessed for integrity by agarose gel electrophoresis; samples displaying intact 28S/18S rRNA (~2:1) were used for library preparation. Stranded RNA-seq libraries were generated from 500 ng of RNA using the Illumina Stranded Total RNA Prep, Ligation with a Ribo-Zero Plus kit (Illumina, Cat# 20040529) and uniquely indexed with an Illumina RNA UD Indexes Set B (Cat# 20091657). Library fragment size (375–475 bp) and quality were evaluated on an Agilent 2200 TapeStation using D1000 ScreenTape (Agilent, Cat# 5067-5582) and reagents (Cat# 5067-5583), and the concentration was normalized to 2 nM. Pooled libraries were diluted to 0.6 nM and sequenced on an Illumina NovaSeq platform (Illumina).

The quality of the raw reads and adapter sequences was checked using FastQC (v.0.12.1) (https://www.bioinformatics.babraham.ac.uk/projects/fastqc/). Sequencing reads were mapped using STAR v.2.7.11b (RRID: SCR_004463) against the human genome build (UCSC hg38).

For differential gene expression (DGE), gene-level counts were quantified using HTSeq^102^ (v2.0.3). Only protein-coding and long noncoding RNA genes were included in the differential gene expression (DGE) analysis. Genes were filtered on the basis of sufficient expression coverage (average read count > 10 in at least half of all available cell conditions). Log2-fold changes between experimental conditions were estimated using DESeq2^103^ (v1.38.3), followed by shrinkage with the ashr package^104^ (v2.2.63). Differential expression analyses were performed at two complementary levels. Within-cell line (horizontal), analyses compared parental and resistant counterparts under different treatment conditions. Grouped (“vertical”) analyses combined samples sharing the same biological condition (such as PT-sensitive or PT-resistant) across independent models to identify conserved transcriptional programs while minimizing cell line-specific effects. Only the models represented in both comparison groups were included in the grouped analyses. Volcano plots were generated using the EnhancedVolcano R package (v1.22.0). Statistical significance was determined using Benjamini–Hochberg adjusted *p* values. Gene length was calculated from genomic coordinates derived from the reference GENCODE annotation file. Spearman’s rank correlation coefficients were computed to assess the association between log2-fold changes and gene length. A heatmap was generated using ComplexHeatmap^105^ (v2.10.0), which was subdivided into four categories (very short, medium short, medium long, and very long) on the basis of log-scale gene length quartiles. Network analysis was performed on significantly altered genes (log2FC ≥ 1, adjusted *p* value ≤ 0.01) identified from the RNA-seq dataset and belonging to the KEGG pathway enrichment results, highlighting interactions between the KEGG_Ribosome and KEGG_Spliceosome pathways. Protein‒protein interactions, including both known and predicted associations, were retrieved from the STRING database, and the network was constructed accordingly. Highly interconnected modules were identified using the MCODE algorithm and subsequently annotated on the basis of KEGG pathway enrichment.

Gene set enrichment analysis (GSEA) was performed using the clusterProfiler package^106^ (v4.6.2) for Gene Ontology (GO) biological process terms or KEGG pathways. Genes were ranked using a statistic defined as the sign of the log2-fold change multiplied by the logarithm of the adjusted *p* value. Enrichment scores were computed using an unweighted Kolmogorov–Smirnov–like statistic. Multiple testing correction was performed using the Benjamini–Hochberg procedure, and enriched GO terms were grouped into functional categories on the basis of Gene Ontology term descriptions. All figures related to DGE were generated using ggplot2 (v3.5.2).

Alternative splicing analysis was performed using rMATS^107,108^ (v4.3.0) executed within a Nextflow-based pipeline (https://github.com/TommasoTarchi/autoRNAseq) for reproducible processing. Spliced-in ratios (Ψ) were estimated from splice junction reads for five canonical event types: skipped exons (SE), retained introns (RI), mutually exclusive exons (MXE), alternative 3′ splice sites (A3SS), and alternative 5′ splice sites (A5SS). Differential splicing was considered significant for events with sufficient read coverage (average read count > 5), a false discovery rate (FDR) < 0.05, and an absolute change in the spliced-in ratio (|ΔΨ|) > 0.1. All figures related to alternative splicing analysis were generated using matplotlib (v3.9.2). The percentage distribution across splicing categories is then calculated relative to the total number of significant events within each specific comparison.

Differential gene expression and alternative splicing analyses were performed using GENCODE v44 comprehensive gene annotation (GRCh38.p14, CHR) (https://www.gencodegenes.org/human/release_44.html). The code supporting these analyses is available at the following link (https://github.com/RiccardoGM/EOC-CDK12i-RNASeqAnalysis/tree/main).

The DAVID database^54^ was used for pathway enrichment analysis of genes associated with differential gene expression and splicing events, with significance defined as a Benjamini–Hochberg FDR < 0.05. Functional annotation was performed using KEGG, and enrichment results were visualized in R (v4.4.2). Genes associated with the Fanconi anemia and base excision repair pathways were retrieved and annotated using the NCBI gene portal for functional role and gene length. Gene length categories were assigned on the basis of the classification reported by Brown et al.^109^ (<15 kb: short; 15–100 kb: middle length; >100 kb: long).

### Nascent RNA isolation and qRT‒PCR

Nascent RNA was labeled and isolated using the Click-iT Nascent RNA Capture Kit (Thermo Fisher, Cat# C10365) according to the manufacturer’s instructions. Cells were seeded to achieve 70–80% confluence and, after 24 h, treated with 300 nM SR4835 for 6 h in the presence or absence of 200 nM 5-ethynyluridine (EU; Thermo Fisher, Cat# E10345) for 1, 3, or 6 h. For pulse-chase experiments, cells treated for 6 h with EU ± SR4835 were released into EU-free medium for 24 h to assess RNA turnover. Total RNA was extracted using TRIzol reagent (Invitrogen, Cat# 15596026) and quantified using a NanoDrop (Thermo Fisher). EU-labeled RNA (3 μg) was biotinylated by click chemistry and precipitated following the manufacturer’s protocol. After the RNA concentration was confirmed using a NanoDrop (Thermo Fisher), 1 μg of biotinylated RNA was captured on Dynabeads Streptavidin Magnetic Beads (Invitrogen, Cat# 65601), which were subsequently washed and eluted for downstream applications. Isolated nascent and total RNA were reverse transcribed into cDNA using PrimeScript RT reagent (Takara, Cat# RR036A) following the manufacturer’s protocol. qRT‒PCR was performed using TB Green Premix Ex Taq (Takara, Cat# RR4202) on a CFX96™ Real-Time PCR Detection System (Bio-Rad, Cat# 1855196) with primers specific for ATM (forward primer: GCTGACAATCATCACCAAGT-3’; reverse primer: 5’-GGTTCTCAGCACTATGGGACA-3’), FANCD2 (forward primer: 5’-CATGGCTGTTCGAGACTTCA-3’; reverse primer: 5’-CAGAACATCTTCCCGGTGTT-3’) and RAD51 (forward primer: 5’-GCTGATGAGTTTGGTGTAGCAG-3’; reverse primer: 5’-GGAAGACAGGGAGAGTCGTAGA-3’). For each target gene, nascent RNA expression was normalized to that of GAPDH (forward primer: 5’-GAAGGTGAAGGTCGGAGTC-3’; reverse primer: 5’-GAAGATGGTGATGGGATTTC-3’). The data obtained were analyzed with Bio-Rad CFX Manager software, and the relative transcript abundance was calculated as 2^(-ΔCt)^. The data represent the mean ± SD of three independent biological replicates.

### In silico structural modeling, IDR and MoRFs

The in silico interaction between CDK12 and SFPQ, in the presence or absence of p54^nrb^, was predicted using AlphaFold3^64^ (https://alphafoldserver.com) with default parameters. Full-length FASTA protein sequences were retrieved from UniProt: CDK12 (Q9NYV4), SFPQ (P23246), and p54^nrb^ (Q15233). The predicted models were ranked on the basis of predicted local distance difference test (pLDDT) scores and interchain predicted alignment error (PAE), and the putative interaction interfaces were visualized using ChimeraX (version 1.1). The intrinsically disordered regions of CDK12, SFPQ, and p54^nrb^ were predicted using the online version of Metapredict v3.^110,111^ Full-length protein sequences in FASTA format were retrieved from UniProt and submitted with default parameters to generate disorder profiles for each protein. The identification of molecular recognition features (MoRFs) was performed using the MoRFchibi SYSTEM,^67^ with default parameters. MoRF_CHiBi_*Web*_ (MCW) propensity scores were obtained considering protein disorder (IDP), conservation and local physicochemical sequence properties.

### Immunoblotting and immunoprecipitation

Cells were lysed in cold RIPA buffer (150 mM NaCl, 50 mM Tris-HCl (pH 8), 0.1% SDS, 1% Igepal, and 0.5% NP-40) supplemented with protease inhibitor cocktail (Roche, Cat# 04693124001), 1 mM Na_3_VO_4_, 10 mM NaF and 1 mM DTT. Protein concentrations were assessed using a Bio-Rad protein assay (Bio-Rad, Cat# 5000006). Proteins were separated by 4 to 20% SDS‒polyacrylamide gel electrophoresis (SDS‒PAGE, Criterion Precast Gel, Bio-Rad, Cat# 5671094) and blotted onto a nitrocellulose membrane (Amersham, GE Healthcare, Cat# 10600003). For immunoprecipitation, cell lysates were prepared in HNTG buffer (20 mM HEPES, 150 mM NaCl, 10% glycerol, and 0.1% Triton X-100) and incubated overnight at 4 °C with the primary antibodies anti-CDK12 (Sigma‒Aldrich Cat# ABE1861; RRID: AB_3714856) or anti-SFPQ (Sigma, 1:150; Cat# AV40572; RRID: AB_1856775). The immunocomplexes were precipitated with protein A or G agarose-conjugated beads (GE Healthcare, Cat# 17528001) for an additional 1 h and 30 min at 4 °C. Beads were washed in HNTG buffer, resuspended in 3× Laemmli Sample Buffer (5× Laemmli buffer composition: 50 mM Tris-HCl (pH 6.8), 2% SDS, 10% glycerol, 0.05% bromophenol blue, and 125 mM β-mercaptoethanol), and subjected to SDS‒PAGE for Western blot analysis. The membranes were blocked with EveryBlot Blocking Buffer (Bio-Rad, Cat# 12010020) and incubated at 4 °C overnight with the following primary antibodies: anti-RNA polymerase II, subunit B1 (fosfo CTD Ser-2, 1:1000; Cat# 04-1571; RRID:AB_112123), anti-GAPDH (1:2000; Cat# CB1001; RRID:AB_2107426) and anti-phospho-Histone H2A.X (Ser139, 1:500; Cat# 05-636; RRID:AB_309864) from Millipore; anti-RNA polymerase II (1:1000; Cat# sc-56767; RRID:AB_785522), anti-Vinculin (1:1000; Cat# sc-7649; RRID:AB_2288413) and anti-FANCD2 (1:250; Cat# sc-20022; RRID:AB_2278211) from Santa Cruz Biotechnology; anti-CDC2L5 (1:500; Cat# A301-458A; RRID:AB_960967); anti-Cyclin K (1:1000; Cat# A301-939A; RRID:AB_1547934) from Bethyl; anti-ATM (1:500; Cat# 2873; RRID:AB_2062659); anti-cleaved PARP (Asp214 1:1000; Cat# 5625; RRID:AB_10699459); anti-Cleaved Caspase-3 (Asp175, 1:500; Cat# 9661; RRID:AB_2341188); and anti-CDK12 (1:500, Cat# 11973; RRID:AB_2715688), anti-Myc-Tag (1:1000, Cat# 2276; RRID:AB_331783) from Cell Signaling Technology; anti-RAD51 (1:5000, GeneTex, Cat# GTX70230; RRID:AB_372856), anti-p54^nrb^ (1:1000, BD Biosciences, Cat# 611279; RRID:AB_398807), anti-SFPQ (1:1000, Sigma-Aldrich, Cat# WH0006421M2; RRID:AB_1843565). Detection was performed using horseradish peroxidase (HRP)-conjugated secondary antibodies (Bethyl Laboratories, 1:3000), which were subsequently imaged through Clarity Western ECL Substrate (Bio-Rad, Cat# 1705061) in a ChemiDoc MP Imaging System (Bio-Rad, Cat# 12003154).

### Statistical analyses

Statistical analyses and graphing were performed using GraphPad Prism v10.6.1. Data are expressed as the mean ± SD from at least three independent biological replicates, unless stated otherwise. The sample sizes are reported in the figure legends, and no data were excluded. Comparisons between independent groups assumed a normal distribution and equal variances. Differences among more than two groups were assessed using one-way ANOVA followed by appropriate post hoc tests, whereas pairwise comparisons were evaluated using two-tailed Student’s *t* tests. Fisher’s exact test was applied for nonlinear regression analyses. Significance thresholds were set as * *p* ≤ 0.05, ** *p* ≤ 0.01, and *** *p* ≤ 0.001. For in vivo experiments, animals were randomly assigned to treatment groups, and histopathological analyses were performed in a blinded manner. Kaplan‒Meier survival curves were generated, and the significance was evaluated with the log-rank (Mantel‒Cox) test.

## Supplementary information

## Data Availability

All original code has been deposited and is publicly available at https://github.com/RiccardoGM/EOC-CDK12i-RNASeqAnalysis/tree/main. The raw amplicon-based custom sequencing and RNA-sequencing datasets generated and analyzed during the current study are available in the SRA repository (Bioproject category) on NCBI under accession number PRJNA1423002 and PRJNA1401822, respectively. Additional data and coding scripts for bioinformatic analyses performed are available upon reasonable request to the corresponding author Gustavo Baldassarre (gbaldassarre@cro.it).

## Materials availability

Requests for additional information and resources (cell lines generated in this study) should be directed to and will be fulfilled by the corresponding author Gustavo Baldassarre (gbaldassarre@cro.it) upon reasonable request and completion of a Materials Transfer Agreement.
