# Supplementary material for: CDK12/13 inhibition overcomes platinum resistance by impairing RNA metabolism
Source: Signal Transduct Target Ther. 2026 Sep 3;11:361. doi: 10.1038/s41392-026-02932-w (PMC13538385; doi:10.1038/s41392-026-02932-w)

Supplementary Data S1_Uncropped WB images

Uncropped membranes for Figure 1e

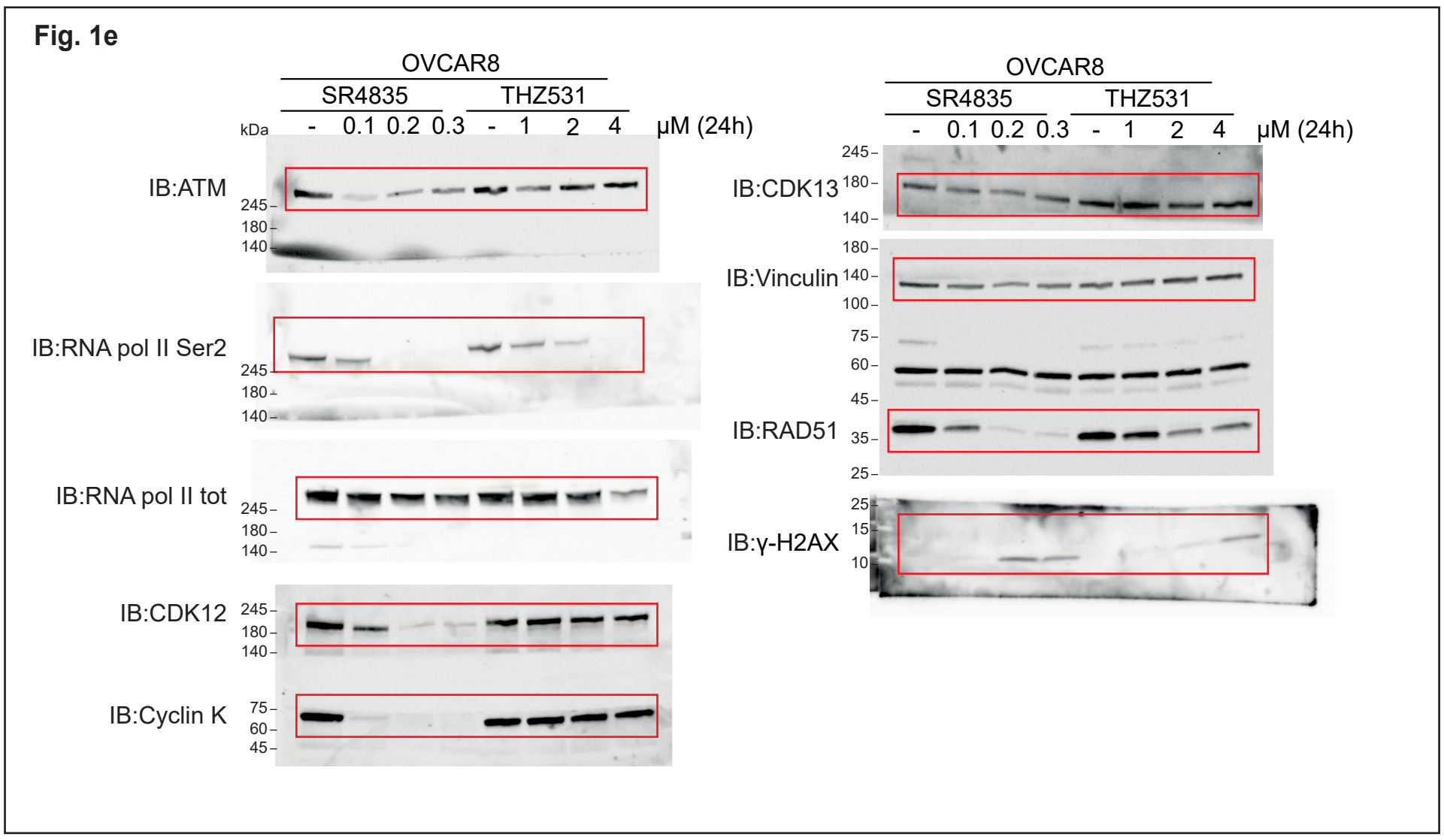

Uncropped membranes for Figure 1g

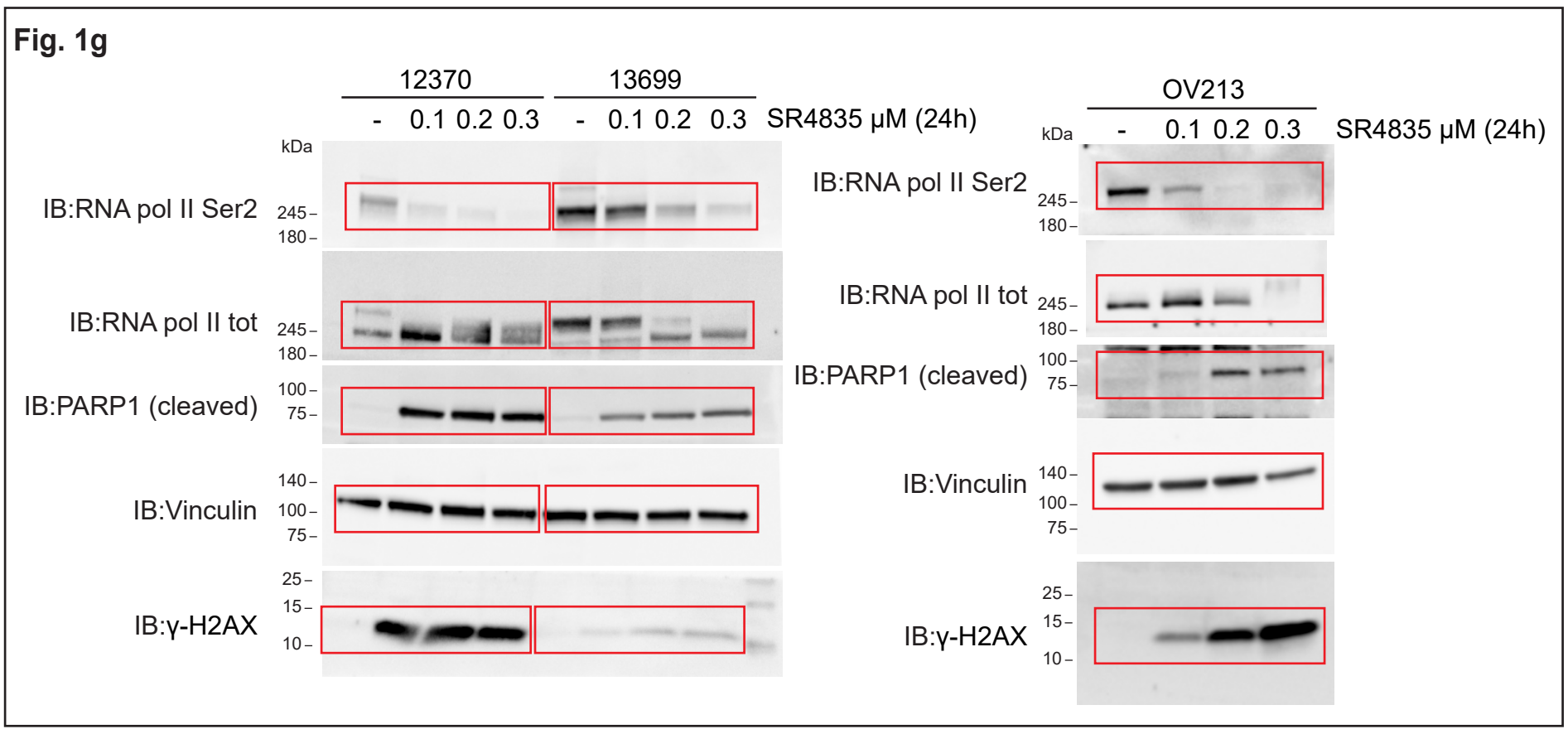

Uncropped membranes for Figure 1h

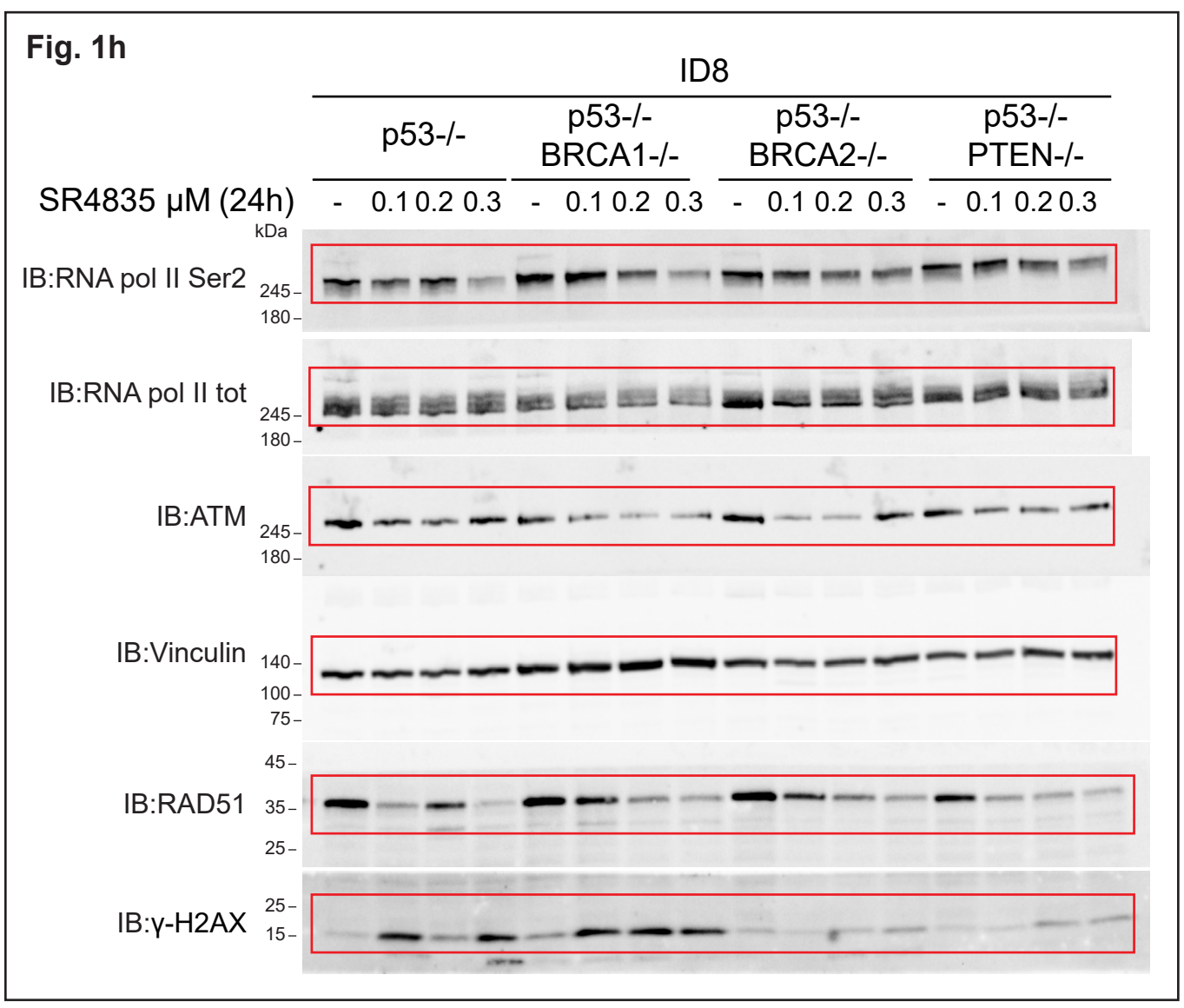

Uncropped membranes for Figure 1k

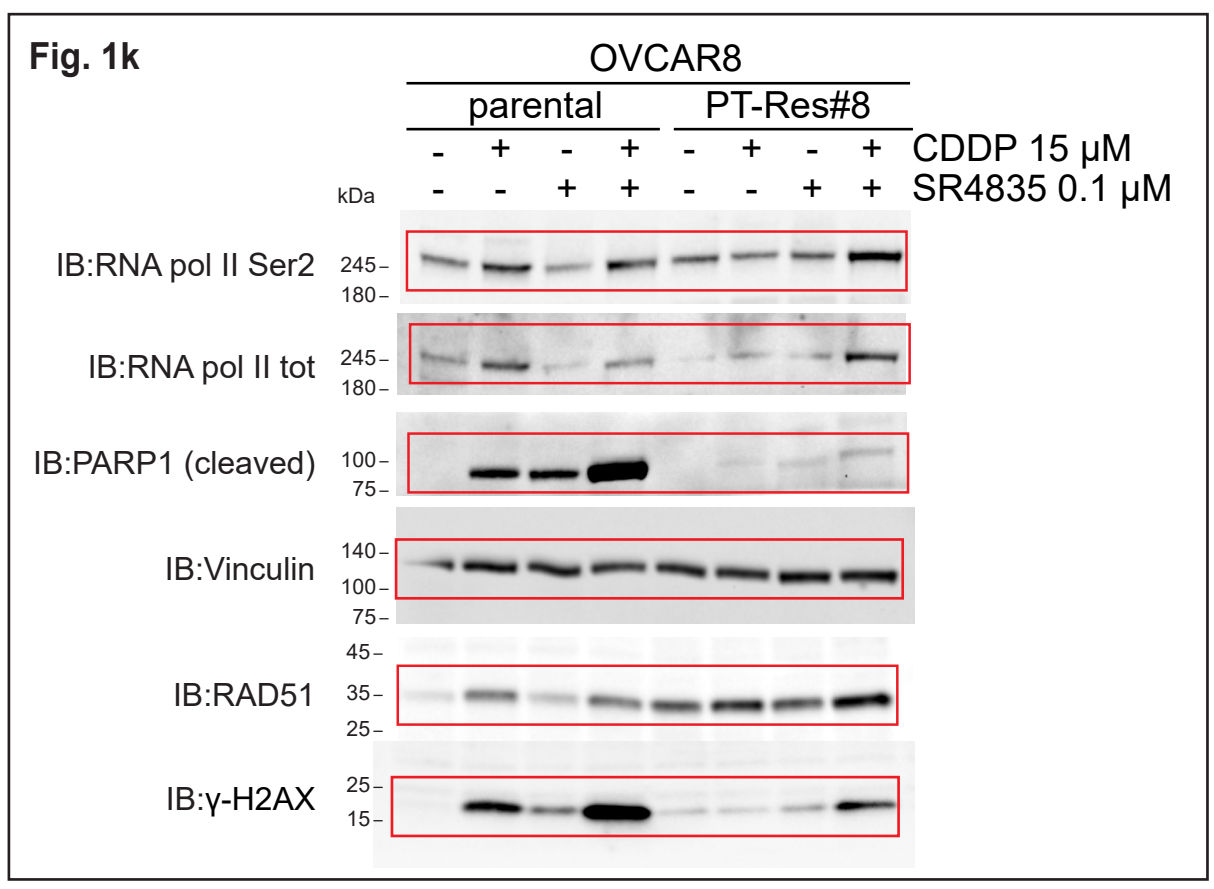

Uncropped membranes for Figure 1m

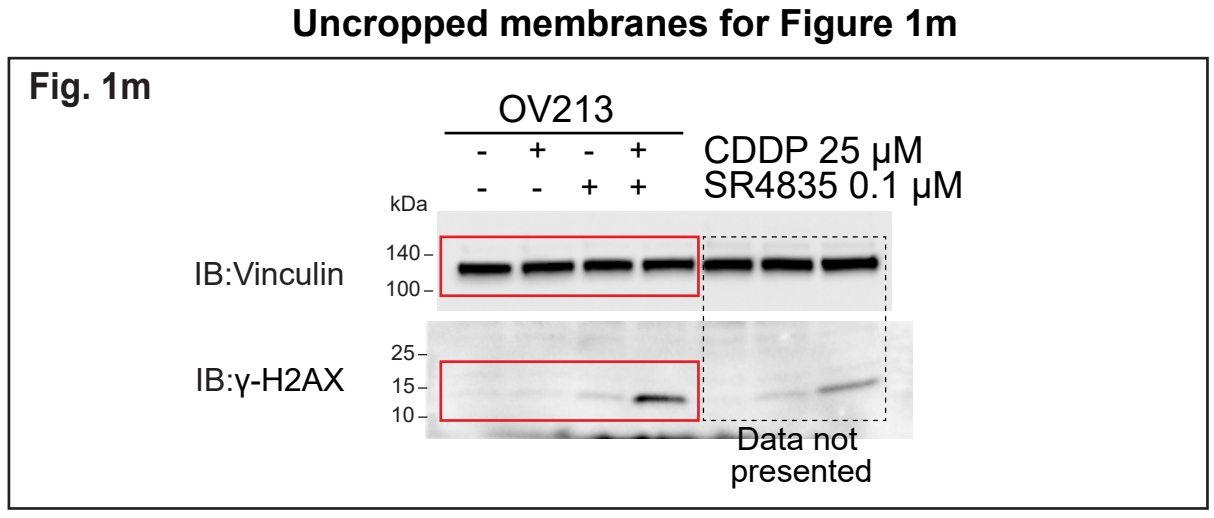

Uncropped membranes for Figure 2g

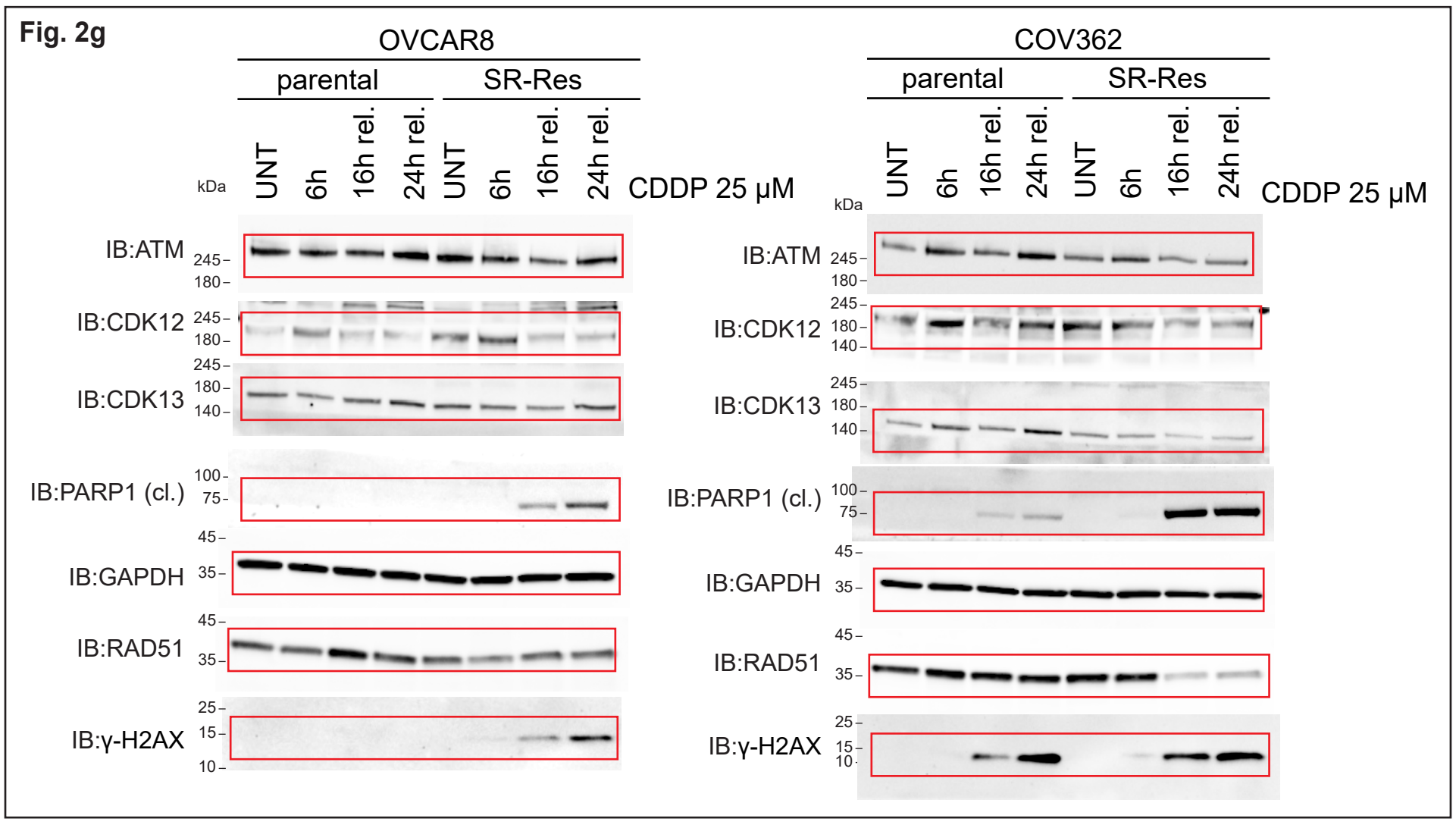

Uncropped membranes for Figure 6b

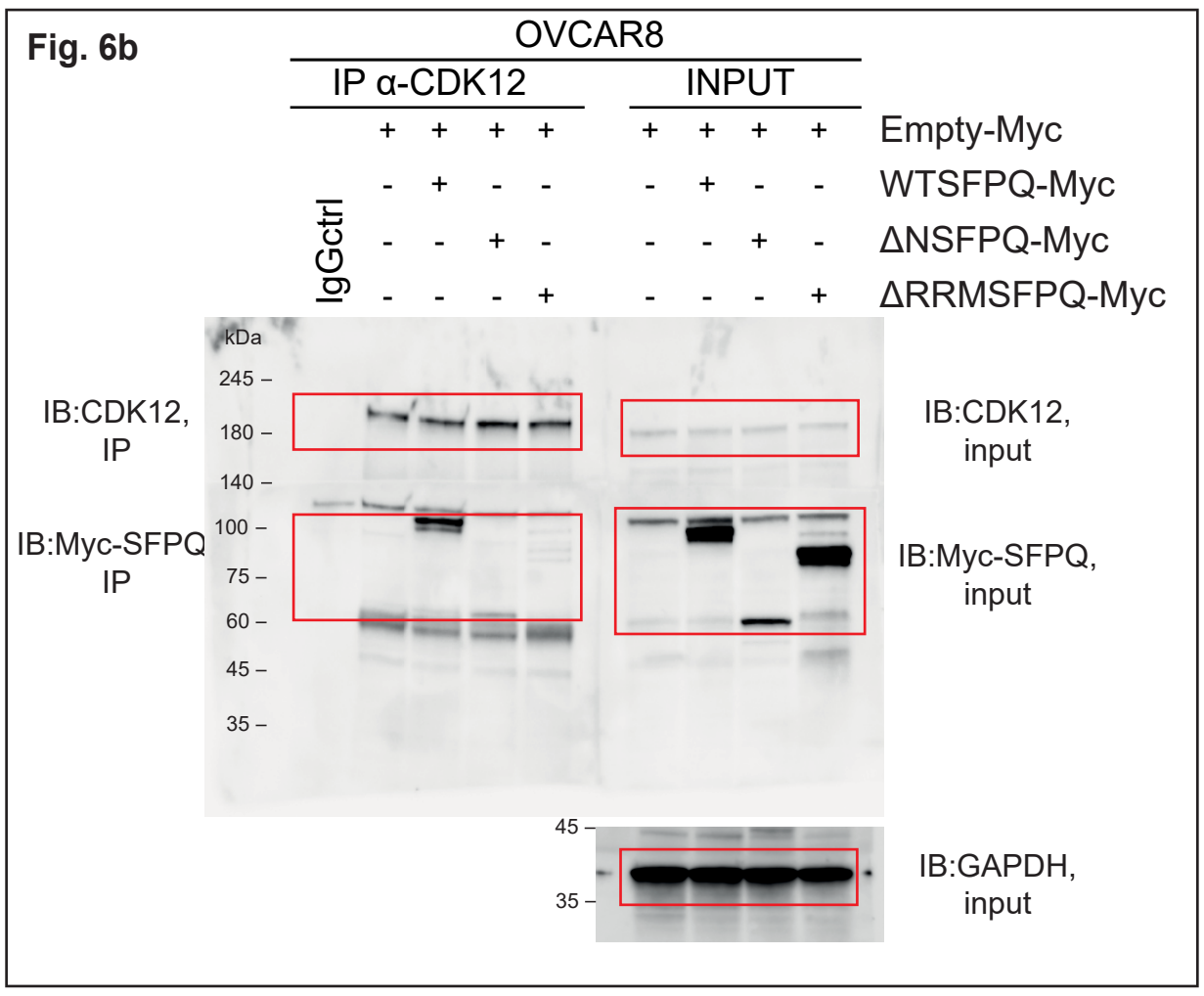

Uncropped membranes for Figure 7a

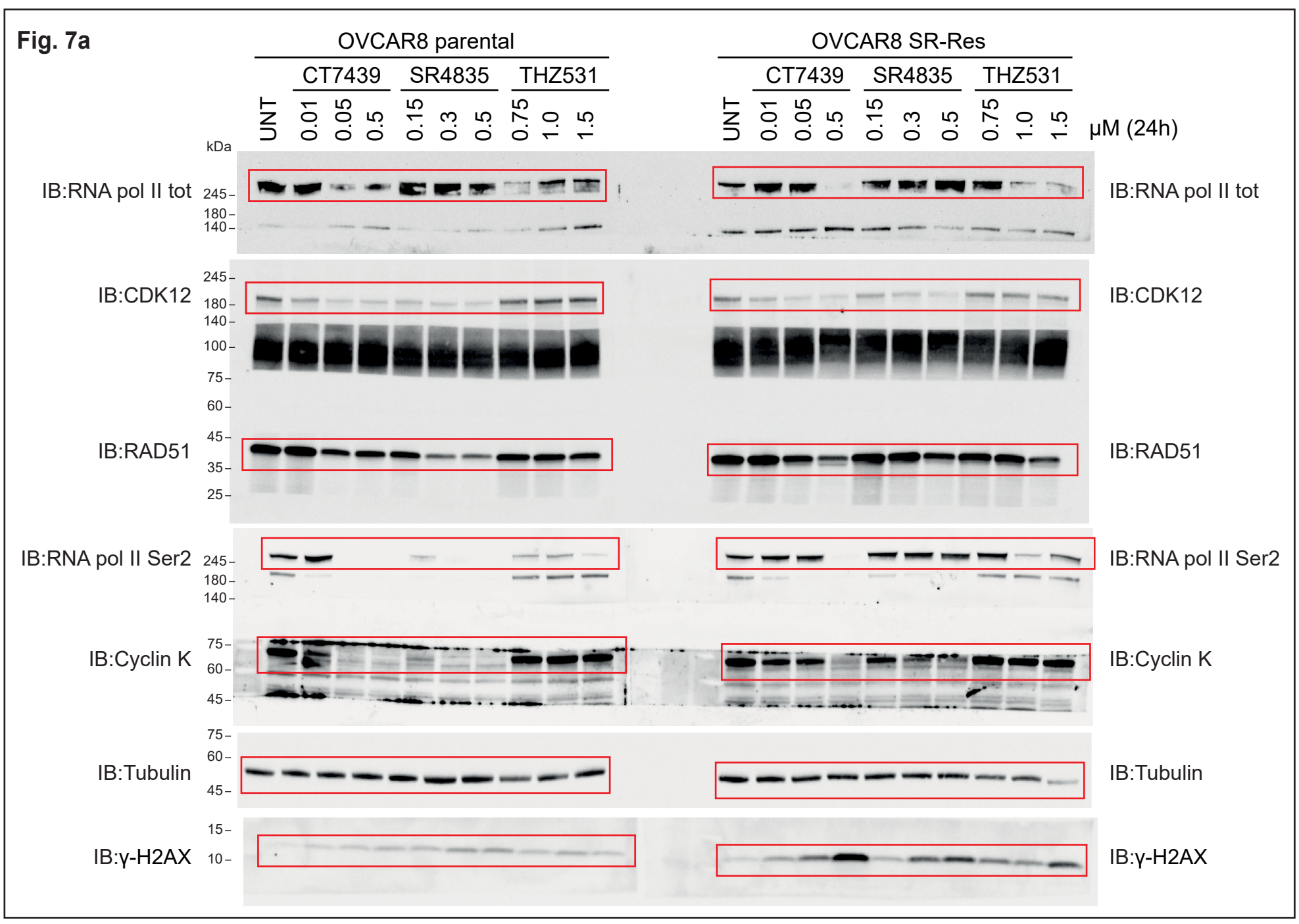

Uncropped membranes for Supplementary Figure 2e

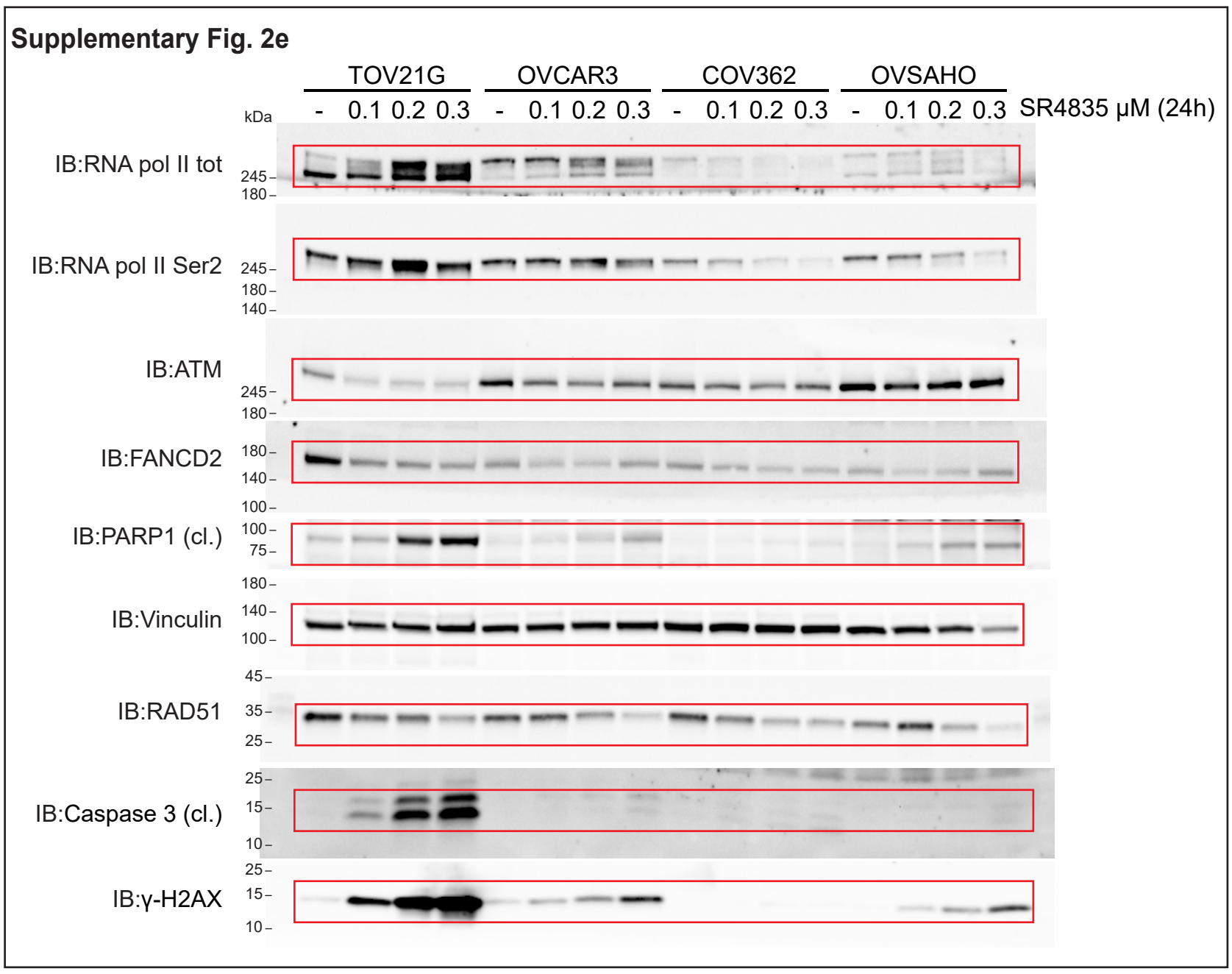

Uncropped membranes for Supplementary Figure 2f

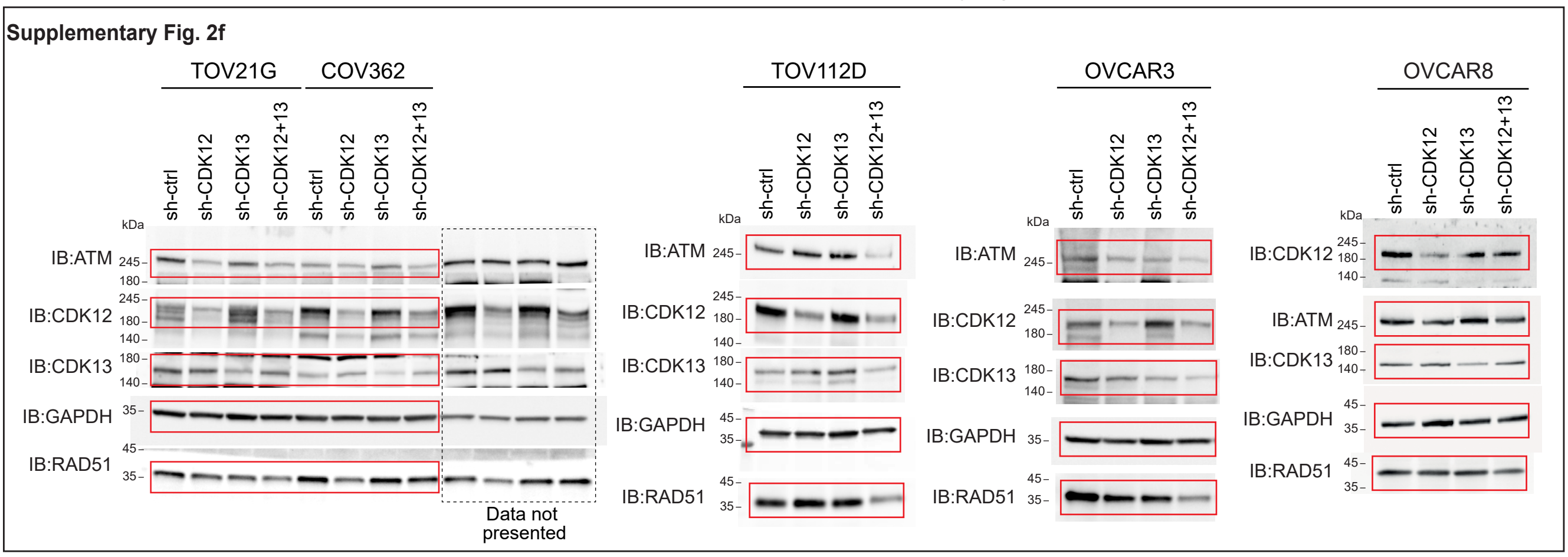

Uncropped membranes for Supplementary Figure 2j

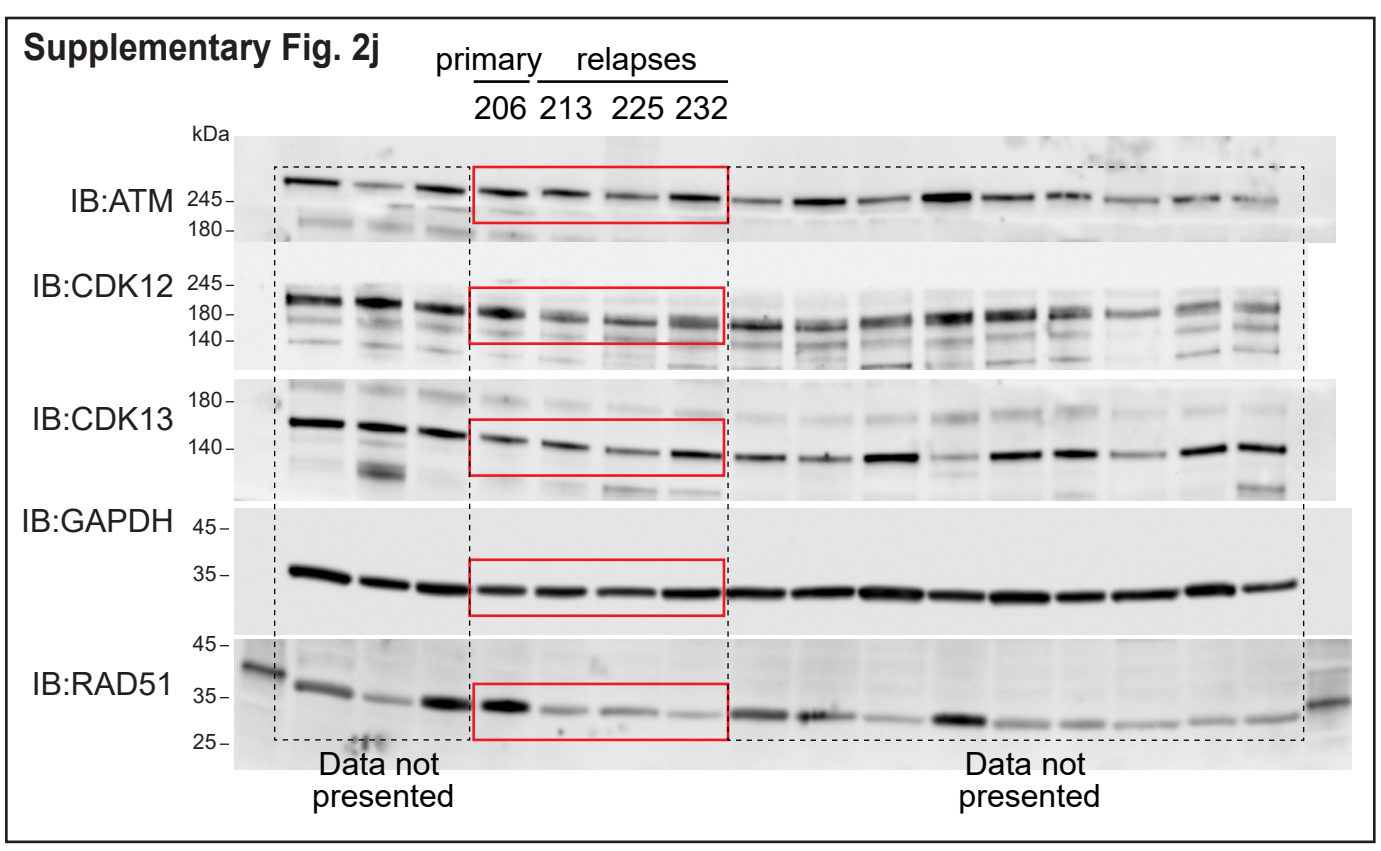

Uncropped membranes for Supplementary Figure 6g

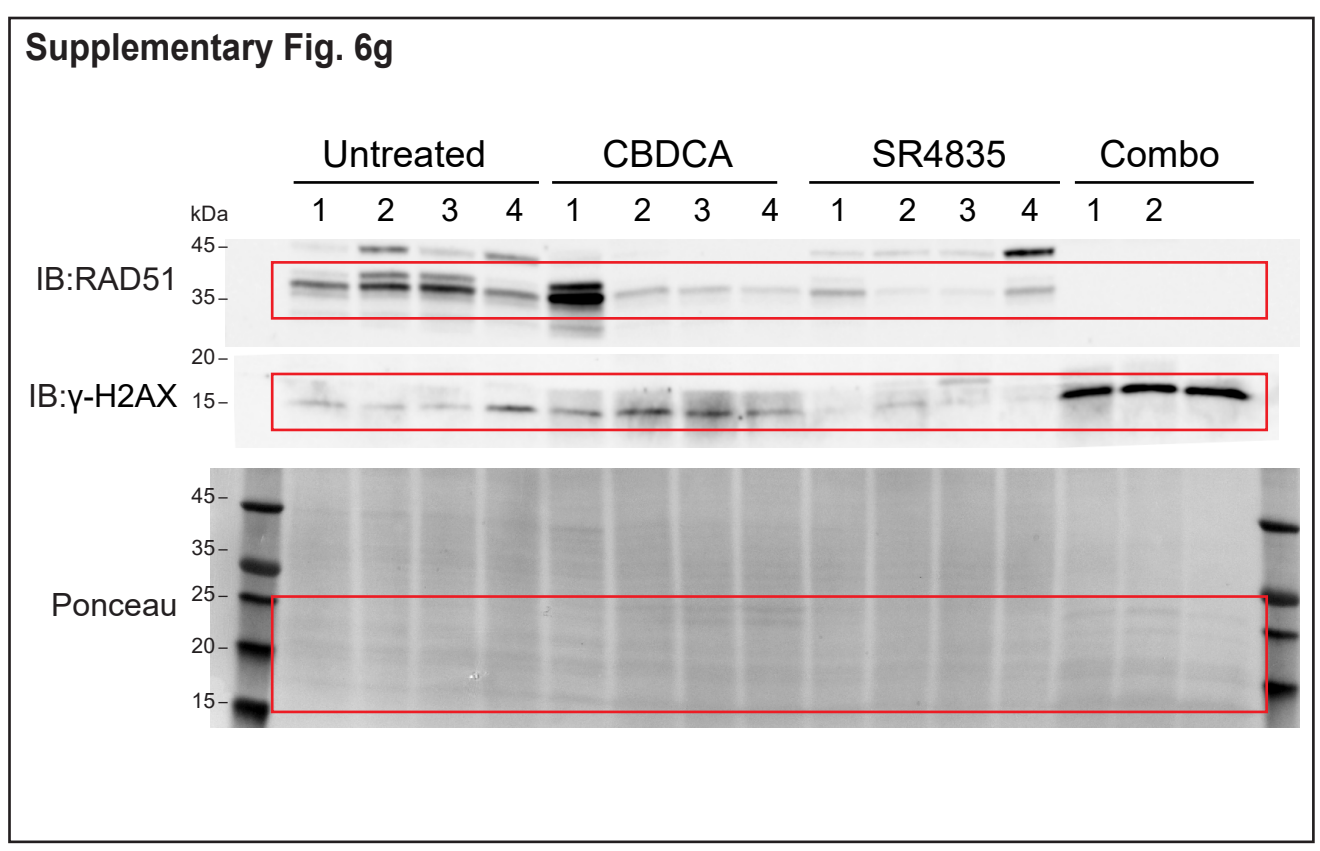

Uncropped membranes for Supplementary Figure 9d

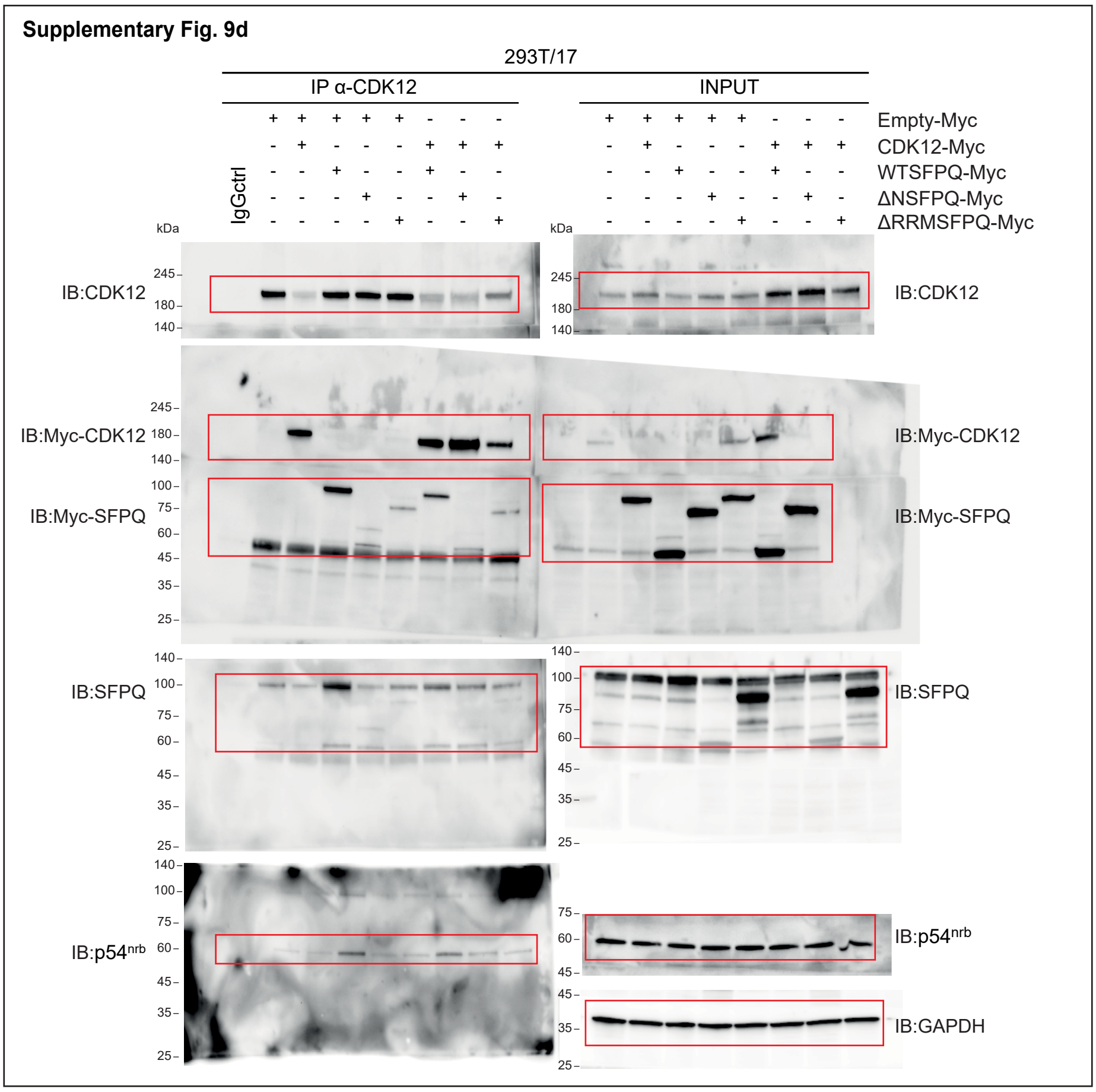

Uncropped membranes for Supplementary Figure 9e

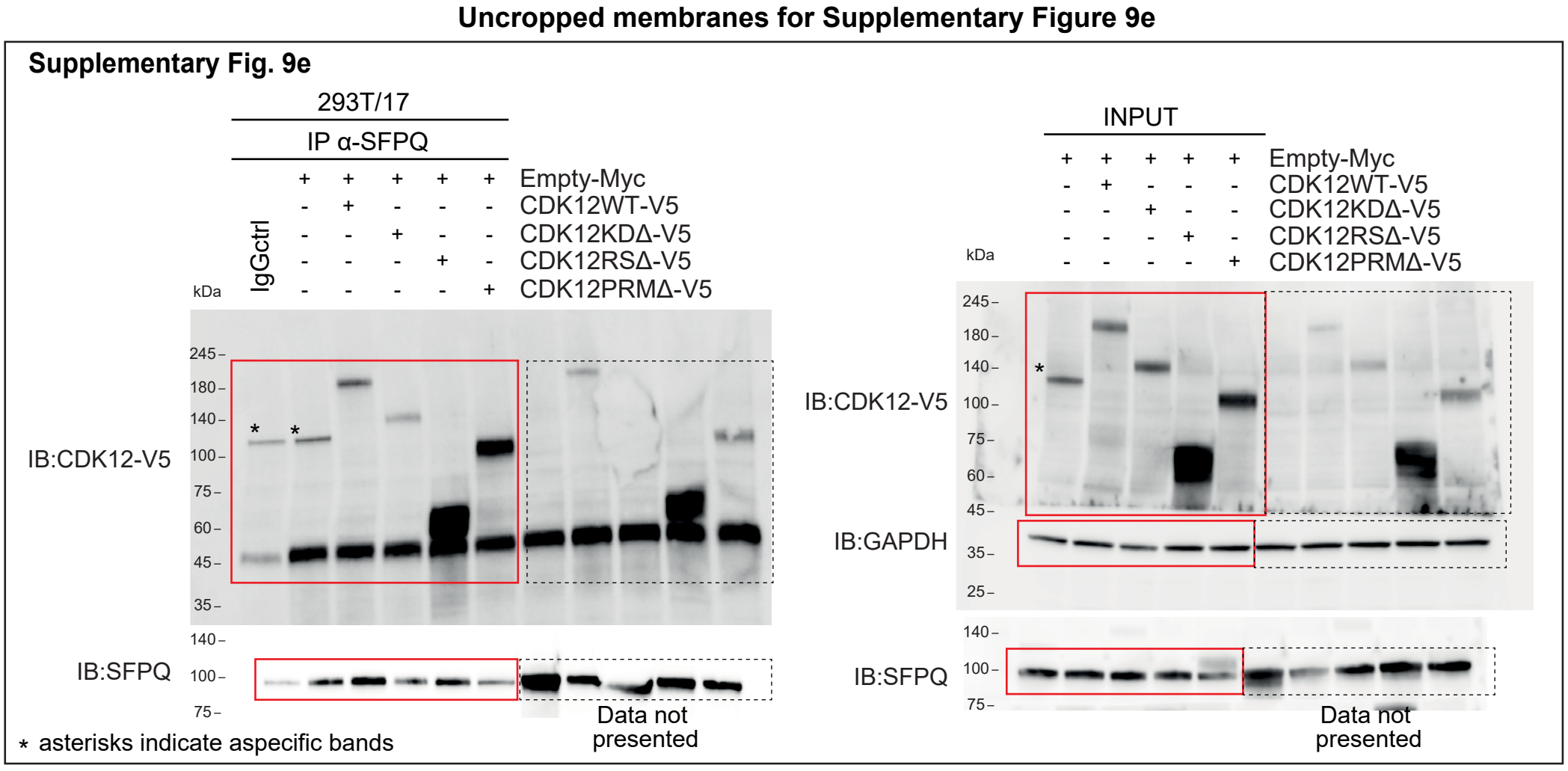

Uncropped membranes for Supplementary Figure 9h

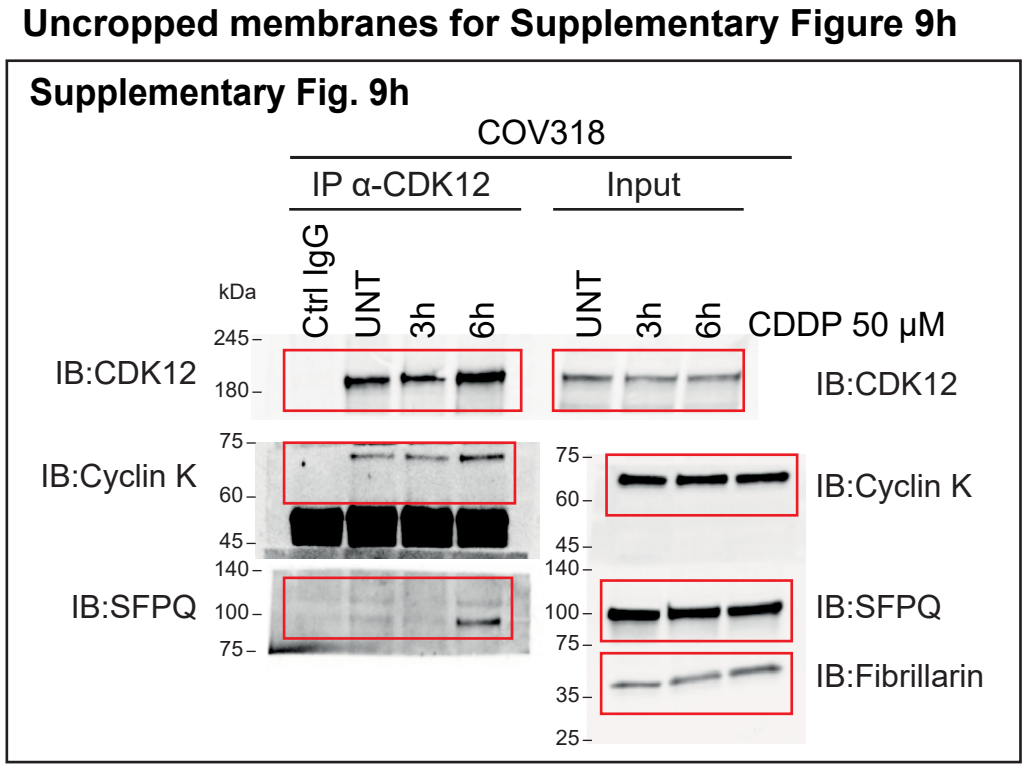

Uncropped membranes for Supplementary Figure 10b

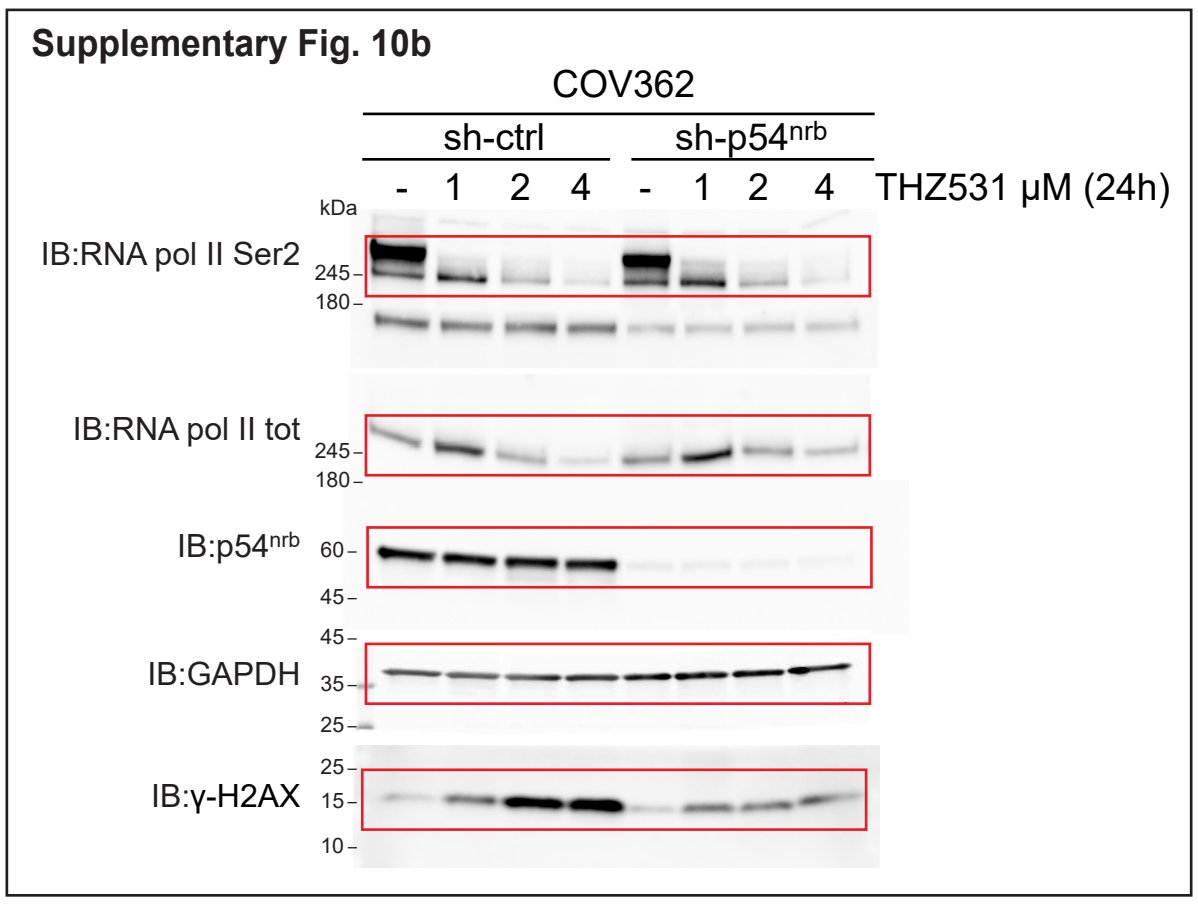

Uncropped membranes for Supplementary Figure 10l

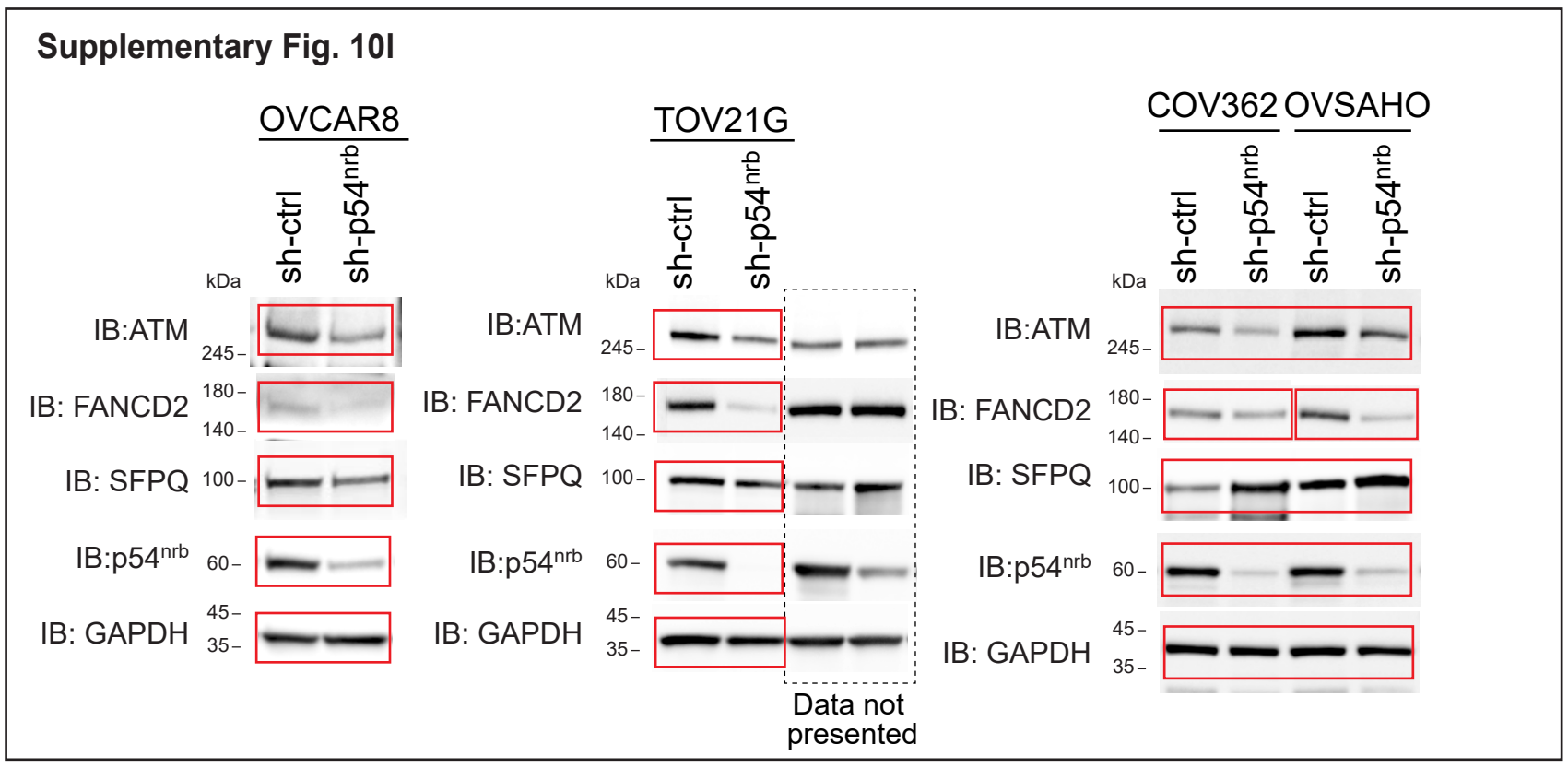

Uncropped membranes for Supplementary Figure 11b

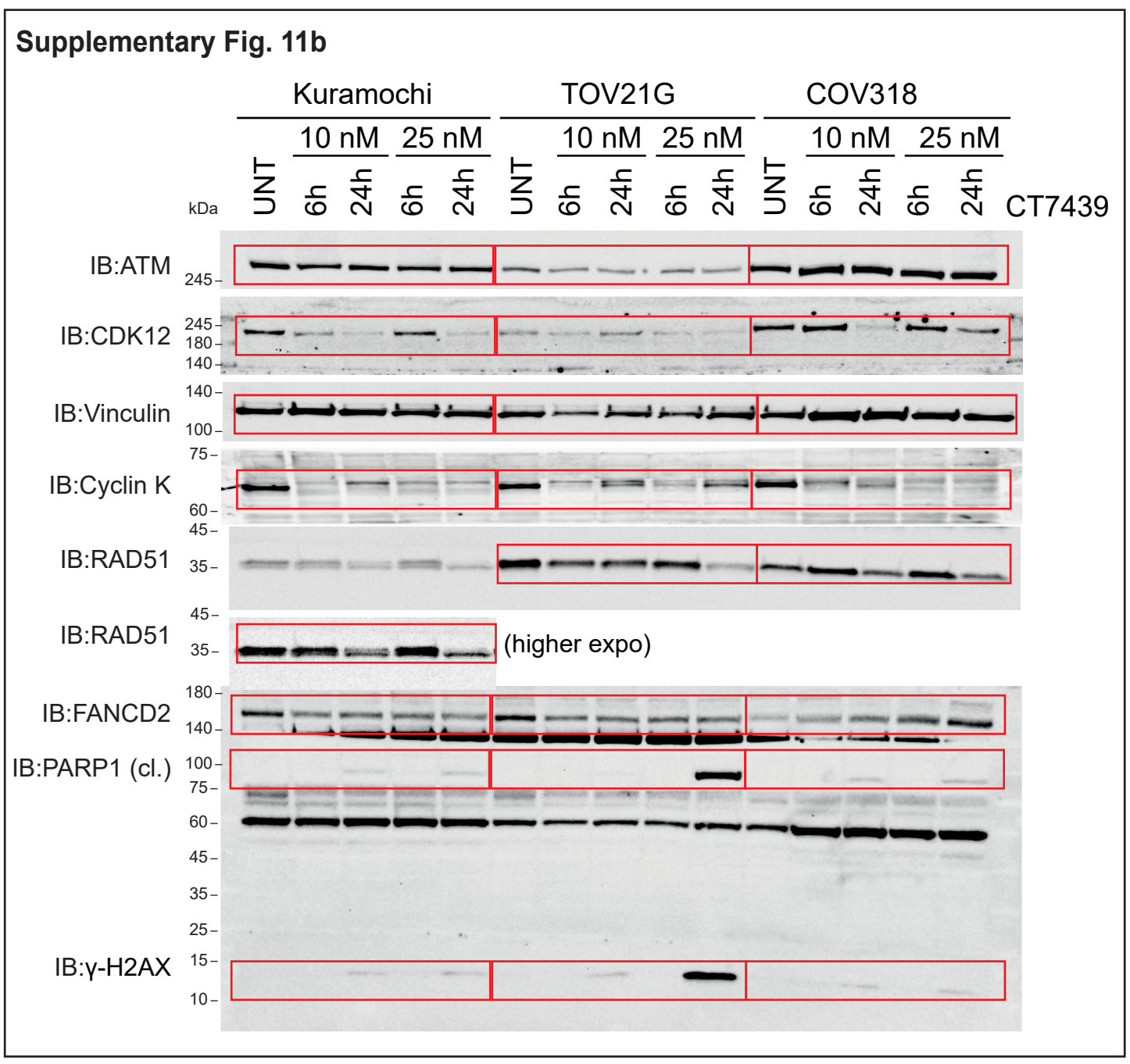

Uncropped membranes for Supplementary Figure 11c

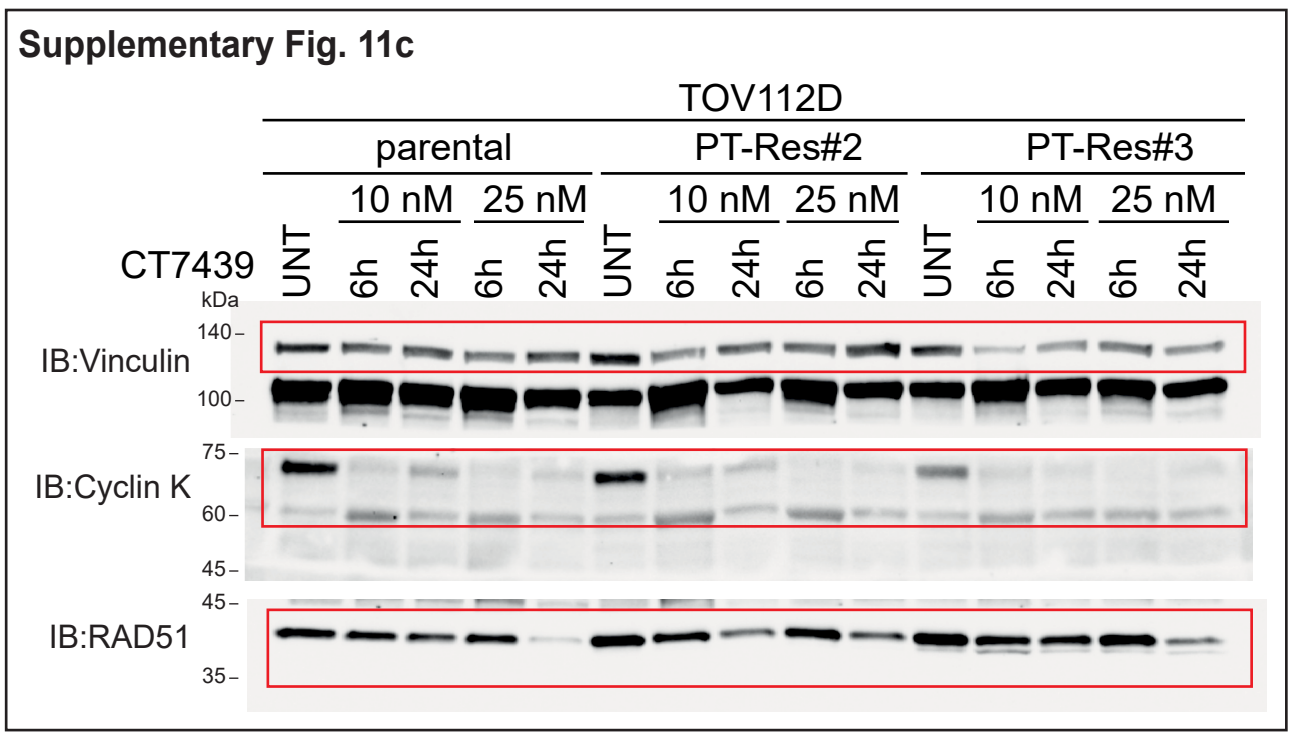

Uncropped membranes for Supplementary Figure 11d

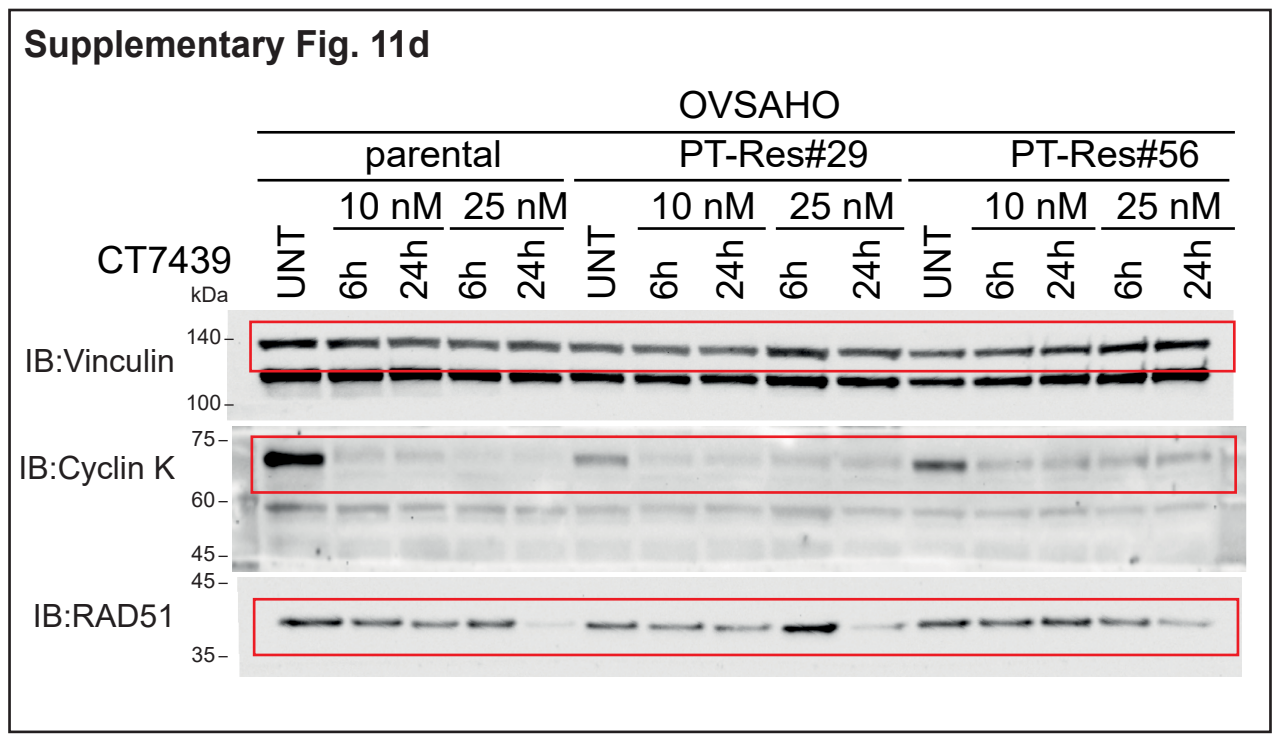

Uncropped membranes for Supplementary Figure 11e

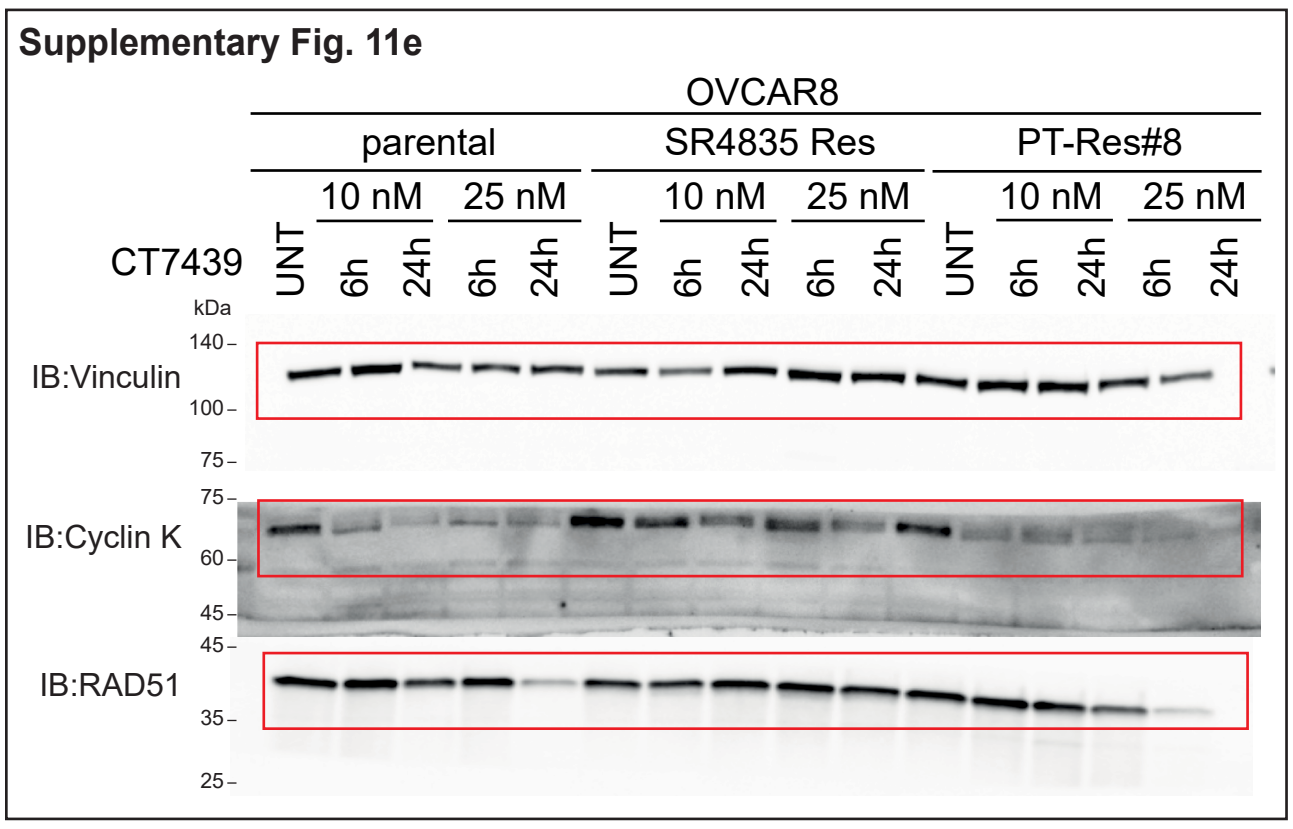

Uncropped membranes for Supplementary Figure 11h

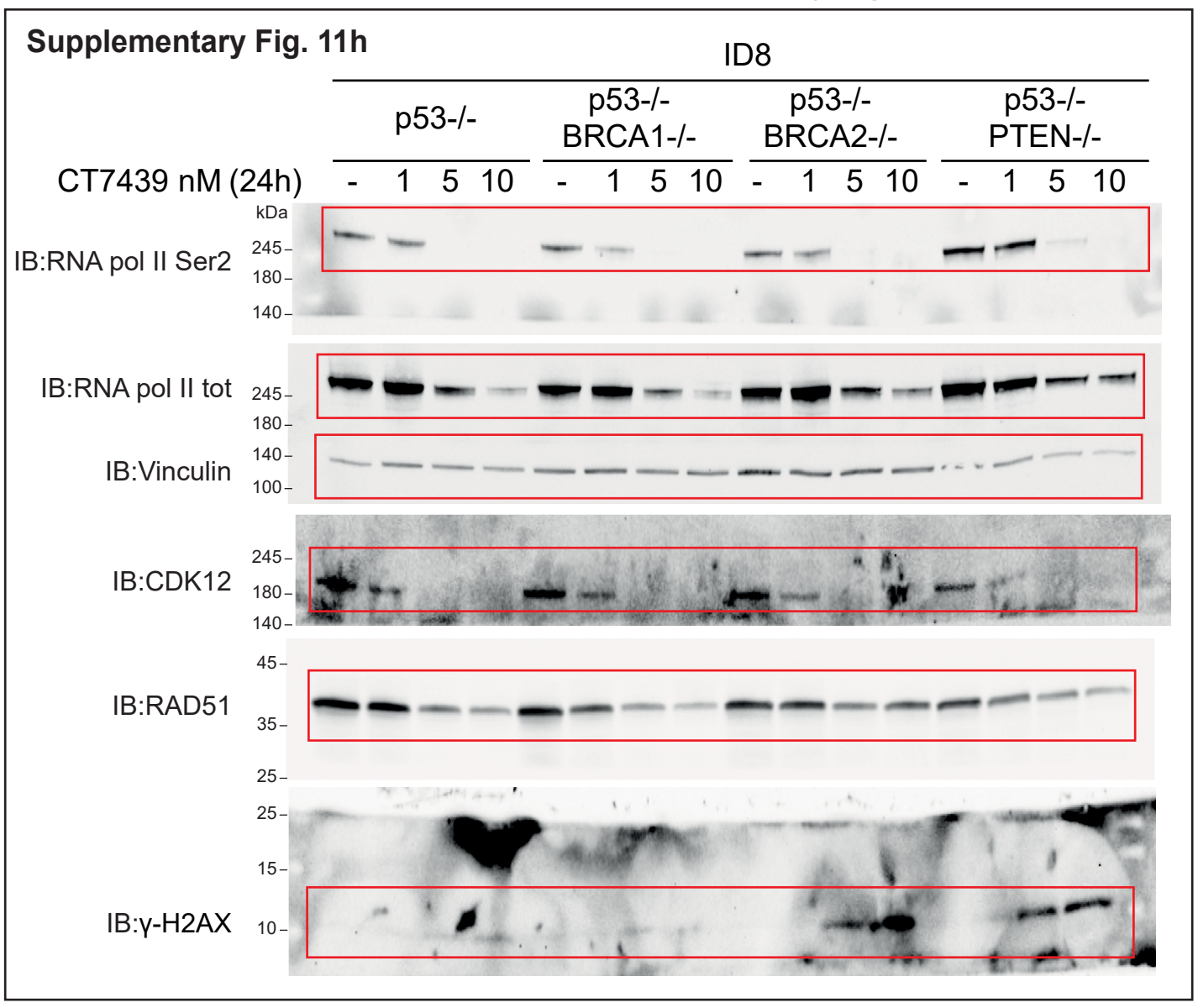

Uncropped membranes for Supplementary Figure 11k

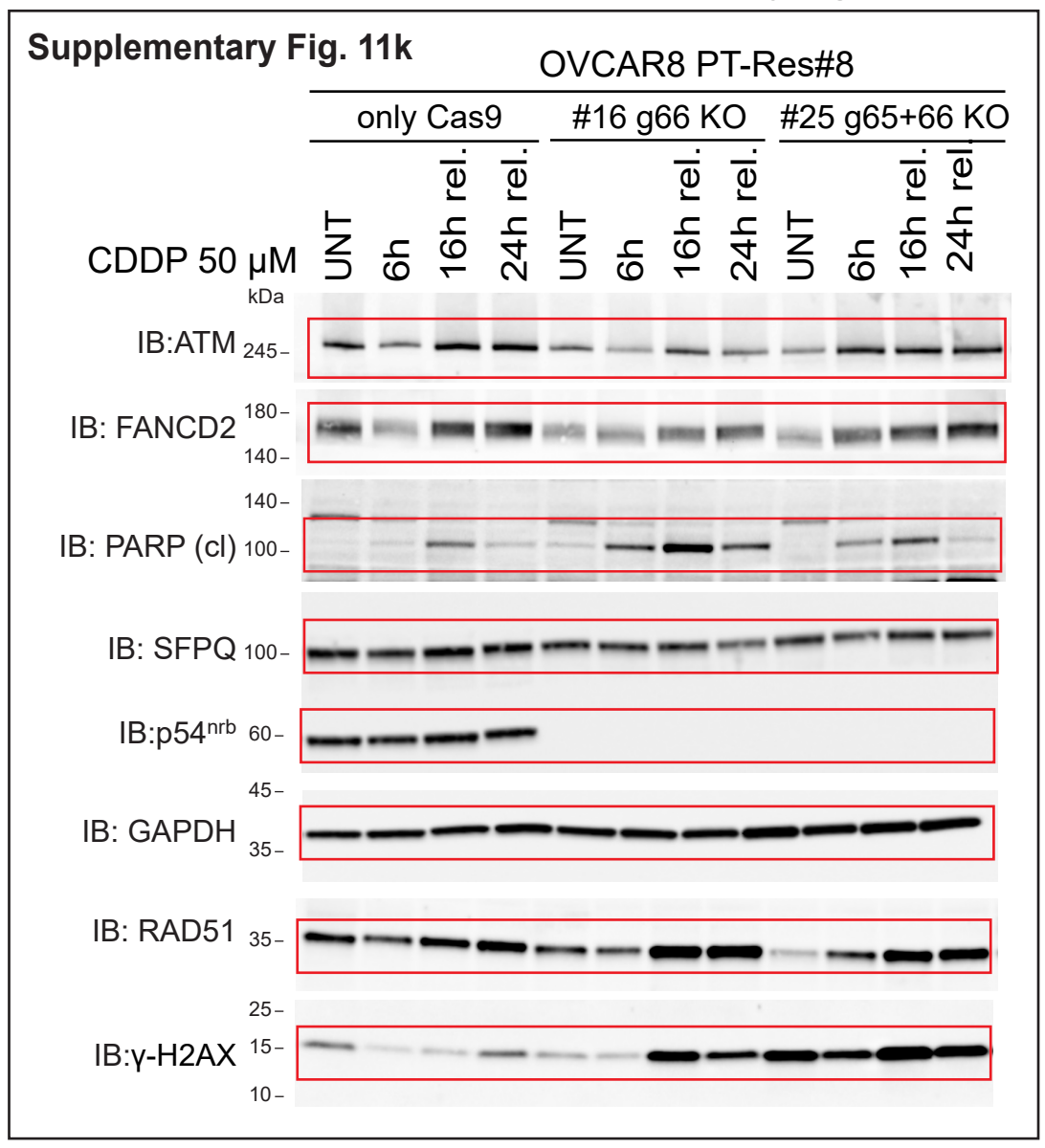

Supplement: Supplementary file 1 [file 41392_2026_2932_MOESM2_ESM.pdf]
