# Supplementary material for: CDK12/13 inhibition overcomes platinum resistance by impairing RNA metabolism
Source: Signal Transduct Target Ther. 2026 Sep 3;11:361. doi: 10.1038/s41392-026-02932-w (PMC13538385; doi:10.1038/s41392-026-02932-w)
Supplement: Supplementary file 2 [file 41392_2026_2932_MOESM1_ESM.docx]

Supplementary Materials for

**CDK12/13 inhibition overcomes platinum resistance by impairing RNA metabolism**

Ilenia Pellarin, Valentina Rossi, Riccardo Giuseppe Margiotta, Javad Karimbayli, Alessio Polacchini, Ashwani Bahl, Milena Nicoloso, Maura Sonego, Sara D'Andrea, Laura Sala, Simone Canesi, Andrea Favero, Anna Nespolo, Danilo Licastro, Michele Bartoletti, Fabio Puglisi, Antonino Ditto, Vincenzo Canzonieri, Monica Schiappacassi, Iain McNeish, Guido Sanguinetti, Barbara Belletti, Gustavo Baldassarre.

Correspondence to: [gbaldassarre@cro.it](mailto:gbaldassarre@cro.it), [ilenia.pellarin@cro.it](mailto:ilenia.pellarin@cro.it)

**This PDF file includes:**

Figures S1 to S12

Tables S1 to S5

**Other Supplementary Materials for this manuscript include:**

Data S1. Supplementary Data S1_Uncropped WB images.pdf (separate file)

Figure S1

**Supplementary Figure 1. Prognostic and predictive values of CDK12 and CDK13 expression in EOC.**

**a-b** Lollipop plots showing CDK12 and CDK13 mutations and Variant of Uncertain Significance (VUS) identified in the cohort of ovarian cancer patient samples collected at our Institution (see Supplementary Table 1 and 2), colored by classification (red for pathogenic, pink for likely pathogenic, light blue for likely benign). Variants are mapped across the protein structure, highlighting the arginine/serine-rich (RS), proline-rich motifs (PRM), and kinase (KD) domains. Annotation tracks, retrieved from cBioPortal, include cancer hotspots, oncogenic mutations, post-translational modifications, and exon organisation.

**c-f** Kaplan-Meier survival curves for Progression Free Survival (PFS) and Overall Survival (OS) for CDK12 (**c** and **d**) and CDK13 (**e** and **f**) wild-type (WT), gain-of-function (GoF) and loss-of-function (LoF) genomic alterations in the cohort of ovarian cancer patient samples collected at our Institution (for clinical variables and response to therapies see Supplementary Table 2). Differences between groups were assessed using the log-rank test. Univariate hazard ratios (HRs) and 95% confidence intervals (CIs) were estimated using Cox proportional hazards regression.

**g-l** Kaplan-Meier survival curves for OS and Disease-Free Survival (DFS) for CDK12 and CDK13 mRNA (**g**, **j**) and protein expression (**h**, **i**, **k** and **l**) in TCGA-OV dataset. Differences between groups were assessed using the log-rank test. Univariate hazard ratios (HRs) and 95% confidence intervals (CIs) were estimated using Cox proportional hazards regression.

**m-n** Boxplots of CDK12 (**m**) and CDK13 (**n**) expression levels between platinum responders and non-responders ovarian cancer patients (RFS at 6 months). Receiver operating characteristic (ROC) curves were computed to assess the area under the curves (AUC) using the online tool ROC plotter (<https://rocplot.com/ovarian/index>).

**Figure S2.**

**Supplementary Figure 2. CDK12i impairs EOC cells viability and affects the DNA Damage Response.**

**a** Heatmap of Log_2_(IC50) values from nonlinear regression analysis of cell viability assays in the different cell lines reported treated with THZ531 or SR4835 for 72 hours. Data represent the mean IC50 of n=3 independent experiments.

**b** Quantification of the clonogenic assay in COV362 cells treated with THZ531 or SR4835 (150 and 300 nM) for 72 hours. Graphs report the percentage of counterstained colonies normalized to the untreated condition, with n=3 independent experiments.

**c-d** Representative IF images (c) of γ-H2AX foci and RAD51 staining, and their quantification (d) in COV362 and OVSAHO cells treated with THZ531 or SR4835 for 24 h.

**e** WB analysis evaluating the expression of the indicated proteins in TOV21G, OVCAR3, COV362 and OVSAHO cells treated with SR4835 for 24 hours. Vinculin served as a loading control.

**f** WB evaluating the expression of the indicated proteins in TOV21G, TOV112D, OVCAR3, COV362 and OVCAR8 cells silenced for CDK12 (sh-CDK12), CDK13 (sh-CDK13) or their combination (sh-CDK12+13). GAPDH was used as a loading control.

**g-h** Nonlinear regression analysis of cell viability assays in OVCAR8 (**g**) and COV362 (**h**) cells silenced for CDK12 (sh-CDK12), CDK13 (sh-CDK13) or their combination (sh-CDK12+13) treated with PT (CDDP) or PARPi (Niraparib) for 72 hours. Data represent the percentage mean (±SD, n=3 biological replicates) of viable cells relative to untreated. The IC50 and the CI are reported in the table.

**i** Nonlinear regression analysis of cell viability assays in 12370, 13699, OV206, OV213, and OV225 primary cells treated with SR4835. Data represent the percentage mean (±SD, n=3 biological replicates) of viable cells relative to untreated. The IC50 and the CI are reported in the tables.

**j** WB evaluating the basal expression of ATM, CDK12/13 and RAD51 in OV206, OV213, OV225, and OV232 primary cells. GAPDH was used as a loading control.

In the figure, the statistical significance was assessed using Fisher's exact test (**a**, **g**, **h** and **i**) or ordinary One-Way ANOVA in (**b** and **d**) and expressed with * p < 0.05, ** p < 0.01, *** p < 0.001.

**Figure S3.**

**Supplementary Figure 3. CDK12i impairs murine cells viability and increases γH2AX levels.**

**a** Nonlinear regression analysis of cell viability assays in genetically modified ID8 murine ovarian cancer cells treated with SR4835 for 72 hours. Data represent the percentage mean (±SD, n=3 biological replicates) of viable cells relative to untreated. The IC50 and the CI are reported in the table.

**b** Representative images of the clonogenic assay in ID8 murine cells treated with increasing doses of SR4835 for 72 hours.

**c** Representative IF images and quantification of γ-H2AX integrated density signal in ID8 murine cells treated with SR4835 for 24 hours, showing increased DNA damage under SR4835 treatment.

In the figure the statistical significance was calculated using Fisher's exact test (**a**) or ordinary One-Way ANOVA in (**c**) and expressed with * p < 0.05, ** p < 0.01, *** p < 0.001.

**Figure S4.**

**Supplementary Figure 4. CDK12i synergistic effect with Cisplatin (CDDP) and PARPi in PT-Res models**

**a-c** Representative dose response curves in OVCAR8 PT-Res clone#3 and #8, treated according to the schedule reported in (**a**). Cells were pre-treated with SR4835 (100 nM) or vehicle for 16 hours, followed by increasing doses of CDDP (**b**) or PARPi (Niraparib, **c**) for 48 hours. Data represent the percentage mean (±SD, n=3 biological replicates) of viable cells relative to untreated. The IC50 and the CI are reported in the tables.

**d** Tables reporting the IC50 and CI (n=3 biological replicates) of nonlinear regression analysis of cell viability assays in TOV21G, OVSAHO parental and PT-Res isogenic clones, treated as in **a**. Cells were either pre-treated with SR4835 (100 nM for TOV21G parental/PT-Res combined with CDDP and OVSAHO parental; 50 nM for TOV21G parental/PT-Res with PARPi; 200 nM for OVSAHO PT-Res) or not for 16 hours, followed by increasing doses of CDDP or PARPi (Niraparib) for 48 hours.

**e** Two-dimensional synergy maps of OVCAR8 parental and PT-Res#8 treated with SR4835 and Niraparib, highlighting synergistic (red) and antagonistic (green) dose regions. The darker red box represents the most synergic area. Highest single agent (HSA) scores are reported (HSA score > 10 = synergism; HSA score < −10 = antagonism).

**f** Representative dose response curves in OV232 PARPi-Res model, treated according to the schedule reported in (**a**). Cells were pre-treated with SR4835 (100 nM) or vehicle for 16 hours, followed by increasing doses of PARPi (Niraparib) for 48 hours. Data represent the percentage mean (±SD, n=3 biological replicates) of viable cells relative to untreated. The IC50 and the CI are reported in the tables.

**g** Tables reporting the IC50 and CI (n=3 biological replicates) of nonlinear regression analysis of cell viability assays in ID8 murine cells, treated as in **a**. Cells were either pre-treated with SR4835 (100 nM) or not for 16 hours, followed by increasing doses of CDDP or PARPi (Niraparib) or Doxorubicin for 48 hours.

The global p-value was calculated using Fisher's exact test in **b**-**d** and **f**-**g**.

**Figure S5.**

**Supplementary Figure 5. Generation and characterization of CDK12i-resistant EOC models.**

**a** Schematic representation of the treatment schedule used to obtain SR4835-resistant (SR-Res) cells (see Methods for further details).

**b-f** Nonlinear regression analysis of cell viability of OVCAR8 and COV362 parental and SR-Res cells treated for 72 hours with increasing doses of SR4835 (**b**, **c**), CDDP (**d**), Niraparib, Doxorubicin and Taxol (**e** and **f**). Data represent the percentage mean (±SD, n=3 biological replicates) of viable cells relative to untreated. The IC50 and the CI are reported in the table.

**g** IF of Acetylated or total Tubulin in OVCAR8 parental and SR-Res cells treated for 6 hours with Taxol (4 µM). Scale bars, 20 μm.

The global p-value was calculated using Fisher's exact test in **b**-**f**.

**Figure S6.**

**Supplementary Figure 6.** **Combined regimen limits tumor burden and offers survival benefit to *in vivo* EOC models.**

**a-b** Representative macroscopic images of the diaphragm and abdominal wall (**a**), and graphs reporting the number of cells in the ascites (**b**) of C57BL/6 mice injected with ID8 p53^−/−^ PTEN^−/−^ murine ovarian cells and treated according to the scheme depicted in Fig. 3a (n=4 mice per condition, endpoint 1). Combo-treated mice showed a reduced tumor burden.

**c-f** Graphs reporting the number of cells in ascites (**c**); the radar plot of total abdominal metastases (**d**); and the quantification of site-specific lesions (reproductive tract and abdominal wall) (**e**, **f**) in C57BL/6 mice injected with ID8 p53^−/−^ PTEN^−/−^ ovarian cancer cells and treated as in Fig. 3a (n=8 per group, endpoint 2).

**g** WB analysis of cells collected from ascites fluids of C57BL/6 mice injected with ID8 p53^−/−^ PTEN^−/−^ murine ovarian cells and treated according to the scheme reported in Fig. 3a (n=8 mice per condition, endpoint 2), showing increased DNA damage marker expression (γ-H2AX) and reduced RAD51 expression in the combo-treated mice.

**h-m** Representative macroscopic images of the diaphragm and abdominal wall (**h**); the graph reporting ascites volume (**i**) and ascites cell number (**j**); the Kaplan-Meier survival curve (**k**); the quantification (**l**) and the distribution (radar plot in **m**) of total abdominal metastases in NU/NU CD1 mice injected with ID8 p53^−/−^ PTEN^−/−^ ovarian cancer cells and treated as in Fig. 3a (n=5 per group).

In the figure the significance was assessed using One-Way ANOVA in (**b**, **c**, **e**, **f**, **i**, **j** and **l**) or the log-rank (Mantel-Cox) test (**k**) and expressed with * p < 0.05, ** p < 0.01, *** p < 0.001.

**Figure S7.**

**Supplementary Figure 7. RNA-seq correlation analysis of gene expression based on gene length.**

**a** Schematic representation of the high coverage RNA-seq experimental design, showing all ovarian cancer models included, treatment conditions, and the analytical workflow used for within-cell-line ("horizontal") and grouped ("vertical") differential expression analyses. Sequencing was performed using the NovaSeq platform (see Methods for details).

**b** Heatmap showing differentially expressed genes (DEGs (Log_2_(FC))>=2.5 and p.adj<=0.05) in untreated, CDDP- or SR4835-treated sensitive and PT-Res cells, pooled according to cellular background (sensitive or resistant) and treatment condition (see Supplementary Fig. 7a for detailed list of cell lines included) and divided into four categories according to gene length (very short, medium short, medium long, very long) based on the quartiles.

**c-d** Volcano plot analysis of DEGs in CDDP-treated grouped PT-Res cells compared to their untreated condition (**c**) and SR-Res compared to parental cells (**d**), obtained from (n=3) biological replicates per cell line and per treatment condition (see Supplementary Fig. 7a for detailed list of cell lines included). Significant DEGs are reported in red (adjusted p-value <0.05 and Log_2_ of the fold change expression (FC)<-2 or >+2); blue (adjusted p-value <0.05); grey (not significant).

**e-f** KEGG pathway enrichment analysis performed using DAVID on the selected high-contributing genes (|Log Fold Change| ≥ 1.5; PC2 loading percentile ≥75 or ≤25). Significance was defined as Benjamini-Hochberg FDR<0.05, n=3 biological replicates.

**g-h** Representative PCA loading plots for parental versus PT-Res OVCAR8 cells under untreated (**g**) and CDDP-treated (**h**) conditions. Labelled genes represent selected transcripts with the highest positive and negative PC2 loading values. Dot color indicates Log Fold Change, whereas dot size represents the −Log10 adjusted p-value.

**i-j** Scatterplots showing Spearman’s rank correlation between Log Fold Change (Log_2_(FC)) and gene length for differentially up- and downregulated genes in SR4835- (**e**) and CDDP-treated (**f**) OVCAR8 parental, PT-Res and SR-Res cells compared to their untreated counterparts.

**Figure S8.**

**Supplementary Figure 8. CDK12i and CDDP impact on alternative splicing events, modulating the DDR pathways.**

**a-b** Graphs representing the percentages of significantly altered splicing events (blue), with respect to all other events (grey), in CDDP-treated parental, PT-Res TOV112D and OVSAHO cells (**a**) and COV362 parental and SR-Res cells (**b**) on all annotated splicing events. Percentages are calculated out of all mapped splicing events. Data represent n=3 biological replicates.

**c-e** Graphs showing the percentage of significantly altered alternative splicing events with increased spliced-in ratio (ΔΨ>0.1) across splicing event categories, over the total number of events in each condition, comparing COV362 parental and SR-Res counterpart (**c**); CDDP-treated parental, PT-Res TOV112D, OVSAHO and primary cells to their untreated condition (**d**) and CDDP-treated parental and SR-Res COV362 cells to their untreated control (**e**). Data represent n=3 biological replicates for each condition.

**f-g** Graphs representing the percentages of significantly altered splicing events (blue), with respect to all other events (grey), in untreated and SR4835-treated parental, PT-Res TOV112D and OVSAHO cells (**f**) and COV362 parental and SR-Res cells (**g**) on all annotated splicing events. Percentages are calculated out of all mapped splicing events. Data represent n=3 biological replicates.

**h-i** Graphs showing the percentage of significantly altered alternative splicing events with increased spliced-in ratio (ΔΨ>0.1) across splicing event categories, over the total number of events in each condition, comparing SR4835-treated parental, PT-Res TOV112D, OVSAHO and primary cells to their untreated condition (**h**) and SR4835-treated parental and SR-Res COV362 cells to their untreated control (**i**). Data represent n=3 biological replicates.

**j-k** DAVID pathway enrichment analyses using the gene lists of significantly altered splicing events in each category, showing enrichment for gene sets in untreated and CDDP-treated COV362 parental (**j**), and SR-Res cells (**k**). Significance was defined as Benjamini-Hochberg FDR<0.05, n=3 biological replicates.

Figure S9.

**Supplementary Figure 9. CDK12 interplay with the SFPQ/p54^nrb^ master regulator splicing complex.**

**a** Network of significantly altered genes (Log_2_(FC)>=1 and p.adj<=0.01) belonging to KEGG_term enrichment analysis, interconnecting KEGG_Ribosome and KEGG_Spliceosome pathways. Edges represent known and predicted protein–protein interactions, and clusters denote highly interconnected modules (see Methods for further details).

**b** AlphaFold3 prediction of CDK12-SFPQ/p54^nrb^ complex structure, showing (see insets) multiple H-bonds between CDK12 and SFPQ residues, reporting global confidence metrics. Scale bars 20 pixels.

**c** Schematic representation of SFPQ and CDK12 WT and deletion mutants. RNA recognition motifs (RRM1/2), NonA/paraspeckle domain (NOPS), coiled-coil domain, low-complexity regions (GPQ and G-rich), and the DNA-binding domain (DBD) are indicated. The ΔN (Δ9–264) and ΔRRM (Δ298–429) SFPQ mutants used in this study are shown. For CDK12, full-length protein and truncation mutants are depicted: KDΔ (Δ1020–end; kinase domain deletion), PRMΔ (Δ700–end; proline-rich motifs deletion), and RSΔ (Δ386–end; arginine/serine-rich domain deletion).

**d** Co-immunoprecipitation (co-IP) analysis of the interaction between CDK12 and SFPQ/p54^nrb^ proteins in HEK293T/17 cells, overexpressing CDK12-Myc and/or SFPQ WT or Myc tagged-mutants. GAPDH was used as a loading control. Arrows indicate specific bands related to SFPQ mutants.

**e** Co-immunoprecipitation (co-IP) analysis of CDK12/SFPQ interaction in HEK293T/17 cells, overexpressing CDK12 WT and mutants-V5 tagged. GAPDH was used as a loading control. Asterisks indicate aspecific bands.

**f-g** Representative images and quantification of PLA between SFPQ-CDK12 (**g**) and p54^nrb^-CDK12 (**e**) in COV318 and COV362 cells treated with SR4835 (300 nM) for 24 hours. In (**f**) and (**g**), the graphs report the number of PLA dots/cell for each condition, expressed as mean ±SD from n=3 biological replicates. A two-tailed, unpaired Student's t-test was used to test significance. Scale bars, 20 μm.

**h** Co-immunoprecipitation (co-IP) analysis of CDK12/Cyclin K-SFPQ interaction in nuclear extracts of COV318 cells treated with CDDP. Fibrillarin was used as a loading control.

**i-j** Plots representing intrinsically disordered regions (IDRs, **i**) and molecular recognition features or elements (MoRFs, **j**) of CDK12, SFPQ, and p54^nrb^. In (**j**), the blue line indicates intrinsic disorder predictions (IDP), while the red represents the MoRF_CHiBi__*_Web_* (MCW) values (see Methods for further details).

Figure S10.

**Supplementary Figure 10. p54^nrb^ is a key mediator of CDK12-SFPQ interaction and cooperates with CDK12 to regulate the expression of DDR genes.**

**a** Tables reporting the IC50 and confidence interval (CI) (n=3 biological replicates) of nonlinear regression analyses evaluating cell viability in the indicated cell lines treated with increasing doses of SR4835 and CDDP for 72 hours. p54^nrb^ silencing renders cells less sensitive to SR4835 and more sensitive to CDDP than control cells (sh-ctrl), mirroring, though with a less pronounced effect, the SR-Res cells' sensitivity to the drugs. Data represent the mean IC50 of n=3 independent experiments.

**b** Nonlinear regression analysis evaluating cell viability in p54^nrb^-silenced (sh-p54^nrb^) COV362 cells treated for 72 hours with increasing doses of THZ531. sh-p54^nrb^ cells are less sensitive to THZ531 than control cells (sh-ctrl). Data represent the mean IC50 of n=3 independent experiments. WB analysis evaluating the expression of the indicated protein in sh-ctrl and sh-p54^nrb^ COV362 cells treated with increasing doses of THZ531 for 24 hours. THZ531 induced lower levels of DNA damage (γ-H2AX) in p54^nrb^-silenced cells than in the control counterpart. GAPDH served as a loading control.

**c** IF analysis evaluating the expression and localization of SFPQ, CDK12 and p54^nrb^ in COV362 parental cells untreated or treated with SR4835 (300 nM) for 1, 3 or 6 hours, as indicated. TO-PRO-3 iodide was used to counterstain nuclei. Scale bar of 20 μm.

**d** Graphs reporting the integrated density and the Coefficient of Variation (CV) quantification of EU-labelled newly transcribed RNAs green signal referred to the representative images reported in Fig. 6f. SR4835 treatment reduced EU incorporation and led to a more diffused signal with lower CV in all cell lines, except SR-Res. The EU signal was basally reduced in p54^nrb^-silenced (sh-p54^nrb^) parental and p54^nrb^ Knock Out (p54^nrb^-KO) PT-Res OVCAR8 cells.

**e** IF analysis of p54^nrb^ localization with EU-labelled newly transcribed RNAs in the indicated OVCAR8 cells treated with EU (1 mM, 1 hour) and SR4835 (300 nM, 6 hours). Scale bars, 20 μm.

**f-h** Graphs reporting the integrated density and the CV quantifications of p54^nrb^ red signal reported in the representative images of panel (**e**). p54^nrb^ signal was basally reduced in sh-p54^nrb^ parental and p54^nrb^-KO PT-Res OVCAR8 cells (**f**). SR4835 treatment led to a more clustered signal with higher CVs in p54^nrb^-WT OVCAR8 cells, but not in SR-Res (**g**-**h**). Scale bar of 20 μm.

**i** Graphs reporting CV quantification of EU-labelled newly transcribed RNAs, green signal, reported in the representative images of Fig. 6h, showing that actinomycin D led to a similar reduction of CV values, indicating a more diffused EU signal, in both OVCAR8 p54^nrb^-KO and Cas9 PT-Res cells.

**j** IF evaluating the expression and localization of SFPQ co-stained with EU-labelled newly transcribed RNAs in p54^nrb^-KO or control (Cas9) OVCAR8 PT-Res cells treated with either CX-5461 (750 nM, 3 hours), SR4835 (300 nM, 6 hours) or their combination. Scale bars, 20 μm. CX-5461 impairs SFPQ clustering under SR4835, while abrogating EU signal in the nucleolus.

**k** IF of Myc-tagged SFPQ-WT and SFPQ mutants (SFPQ-ΔN lacks 9-264 residues; SFPQ-ΔRRM lacks 298-429 residues) co-stained with EU-labelled newly transcribed RNAs in OVCAR8 PT-Res cells treated or not with SR4835 (300 nM, 6 hours). SFPQ ΔRRM localization remained diffused, while SFPQ-WT and SFPQ-ΔN clustered under SR4835 treatment. Scale bars, 20 μm.

**l** WB analysis evaluating the expression of the indicated proteins in OVCAR8, TOV21G, COV362 and OVSAHO cells silenced for p54^nrb^ (sh-p54^nrb^), showing reduced DDR gene expression (ATM, FANCD2 and RAD51) and increased compensatory expression of SFPQ in sh-p54^nrb^ cells compared to control (sh-ctrl) cells. GAPDH was used as a loading control.

In the figure, the statistical significance was assessed using ordinary One-Way ANOVA in (**d** and **f**-**i**) or Fisher's exact test (**a**, **b**) and expressed with * p < 0.05, ** p < 0.01, *** p < 0.001.

**Figure S11.**

**Supplementary Figure 11. CT7439 outperforms other CDK12i in impairing EOC cells' viability and potentiates CDDP treatment.**

**a** Heatmap of Log_2_(IC50) values from nonlinear regression analysis of cell viability assays in the different cell lines reported, treated with THZ531, SR4835 or CT7439 for 72 hours. Data represent the mean IC50 of n=3 independent experiments.

**b** WB analysis evaluating the expression of the indicated proteins in TOV21G, COV318 and Kuramochi cells treated with CT7439 for 6 or 24 hours, showing dose- and time-dependent decreased expression of DDR proteins (ATM, FANCD2 and RAD51), loss of CDK12 and Cyclin-K, and induction of DNA damage (γ-H2AX) and apoptosis markers (cleaved PARP1). Vinculin was used as a loading control.

**c-e** WB analysis evaluating the expression of RAD51 and Cyclin-K in parental and PT-Res isogenic clones TOV112D (**c**) and OVSAHO (**d**) and in OVCAR8 parental, SR-Res and PT-res cells (**e**) treated with CT7439 for 6 or 24 hours. Vinculin was used as a loading control.

**f** Tables reporting the IC50 and confidence interval (CI) (n=3 biological replicates) of nonlinear regression analyses evaluating cell viability in the indicated PT-Res OVCAR8 clones. Cells were pre-treated with CT7439 (7,5 nM) or vehicle for 16 hours, followed by increasing doses of CDDP or PARPi (Niraparib) for 48 hours.

**g** Two-dimensional synergy maps of OVCAR8 parental and PT-Res#8 treated with CT7439 and Niraparib, highlighting synergistic (red) and antagonistic (green) dose regions. The darker red box represents the most synergic area. Highest single agent (HSA) scores are reported (HSA score > 10 = synergism; HSA score < −10 = antagonism).

**h** WB analysis evaluating the expression of the indicated protein in genetically modified ID8 murine ovarian cancer cells treated with CT7439 for 24 hours, showing decreased RNA Pol II Ser2 phosphorylation, RAD51 expression, CDK12 loss, and induction of DNA damage (γ-H2AX). Vinculin was used as a loading control.

**i** Tables reporting the IC50 and confidence interval (CI) (n=3 biological replicates) of nonlinear regression analyses evaluating cell viability in cells described in panel (**h**). Cells were pre-treated with CT7439 (2,5 nM) or vehicle for 16 hours, followed by increasing doses of CDDP or PARPi (Niraparib) for 48 hours.

**j** Nonlinear regression analysis evaluating cell viability in p54^nrb^-KO cells treated for 72 hours with increasing doses of CT7439 or CDDP. p54^nrb^-KO renders cells less sensitive to CT7439 and more sensitive to CDDP than control cells (Cas9). Data represent the mean IC50 of n=3 independent experiments

**k** WB analysis evaluating the expression of the indicated protein in Cas9 and p54^nrb^-KO OVCAR8 PT-Res cells treated with CDDP (50 μM) for 6 hours and then released into CDDP-free medium for 16 (+16h rel.) or 24 hours (+24h rel.) to allow DNA repair. CDDP induced higher levels of DNA damage (γ-H2AX) and response (RAD51) and increased apoptosis (cleaved PARP1) in p54^nrb^-KO cells than in the control counterpart. GAPDH served as a loading control.

**l** IF analysis and Coefficient of Variation (CV) quantification of SFPQ localization with or without EU-labelled newly transcribed RNAs co-staining in control (Cas9) and p54^nrb^-KO OVCAR8 PT-Res cells treated with CT7439 (100 nM, 6 hours) ± EU (1 mM, 1 hour). CT7439 induced SFPQ condensation only in p54^nrb^-WT (Cas9) cells. Scale bars, 20 μm.

In the figure the statistical significance was calculated using ordinary One-Way ANOVA in (**l**) or Fisher's exact test (**a**, **f**, **i** and **j**) and expressed with * p<0.05, ** p<0.01, *** p<0.001.

**Figure S12.**

**Supplementary Figure 12. Combined regimen strongly limits tumor burden and offers survival benefit to EOC mice models.**

**a-e** Representative macroscopic images of the diaphragm and abdominal wall (**b**); graphs reporting the quantification of cells into ascites (**c**) and of site-specific lesions (reproductive tract and diaphragm/abdominal wall) (**d**, **e**) in C57BL/6 mice injected with ID8 p53^−/−^ PTEN^−/−^ ovarian cancer cells and treated as depicted in the schema in panel (**a**) (n = 5 per group). Combo-treated mice showed a reduced tumor burden.

**f-l** Representative macroscopic images of the diaphragm and abdominal wall (**g**); ascites volume (**h**) and cell count in it (**i**); quantification of site-specific lesions (reproductive tract and diaphragm/abdominal wall) (**j**, **k**) and necrotic areas within tumor deposits (**l**) in C57BL/6 mice injected with ID8 p53^−/−^ PTEN^−/−^ ovarian cancer cells and treated as depicted in (**f**) (n = 5 per group). Combo-treated mice showed reduced tumor burden and larger areas of necrosis.

**m-q** Representative macroscopic images of the diaphragm and abdominal wall (**m**); graph reporting the ascites volume (**n**), their cell and spheroid number (**o**, **p**) and the quantification of reproductive tract lesions (**q**) in NSG mice injected with the PT-resistant PDX#OV218.3 and treated according to the scheme depicted in Fig. 7k (n=5 per condition). CT7439- and combo-treated mice showed reduced metastatic spreading.

In the figure the statistical significance was calculated using One-Way ANOVA (**c**-**e**, **h**-**k**, **n**-**q**) or Krustal-Wallis test (**l**) and expressed with * p < 0.05, ** p < 0.01, *** p < 0.001.

**Supplementary Table 1. Cohort of altered EOC patients,**

**(mutations and/or copy number variations, CNV). Related to Fig. 1a-b.**

In the table Copy Number Variation (CNV) values are reported as ratios relative to a normal diploid genome; copy gain (light red) is considered > 2.5 (and 1.25 as ratio) and copy loss < 1.5 (and 0.75 as ratio) (light blue). Variant of Uncertain Significance (VUS) are indicated in orange, while pathogenic mutations are indicated in red.

(MANE Select annotation: CDK5, NM_004935.4; CDK8, NM_001260.3; CDK9, NM_001261.4; CDK10, NM_052988.5; CDK12, NM_016507.4; CDK13, NM_003718.5; CDK15, NM_001261435.1; CDK16, NM_006201.5; CDK18, NM_212502.3).

Missense variant effect predictions were performed using AlphaMissense. The following protein/transcript identifiers were used as input queries:

CDK13 (CDK13_HUMAN, Q14004, ENST00000181839.10); CDK9 (CDK9_HUMAN, P50750, ENST00000373264.5); CDK12 (CDK12_HUMAN, Q9NYV4, ENST00000447079.5);

CDK10 (CDK10_HUMAN, Q15131, ENST00000353379.12). Variants predicted as likely benign are indicated in light blue, whereas variants predicted as likely pathogenic are shown in dark red.

**Supplementary Table 2. Correlation of CDK12 and CDK13 genomic alterations with clinical variables and response to therapies. Related to Supplementary Fig. 1a-f.**

The table reports the clinical variables and response to therapies, related to patient’s samples evaluated in Supplementary Fig. 1a-f.

**Abbreviations:** ***NA***: Not Available; ***PLD***: Pegylated Liposomal Doxorubicin; ***PARPi***: PARP inhibitors; ***WT***: Wild Type; ***GoF and LoF***: Gain of Function and Loss of Function.

**Supplementary Table 3. Table reporting the cell lines evaluated for CDK12i sensitivity. Related to Supplementary Fig. 2a/11a.**

p53 and BRCA1 and 2 mutational status are indicated according to literature data and experimental analysis (AmoyDx HRD panel).

**Abbreviations:** ***EOC***: Epithelial Ovarian Cancer; ***BC***: Breast Cancer; ***HGSOC***: High Grade Serous Ovarian Cancer; ***WT***: Wild Type; ***KO***: Knock Out; ***HER2***: Human Epidermal Growth Factor Receptor 2; ***IHC***: Immunohistochemistry

**Supplementary Table 4**. **Genomic Profiling of OVCAR8 and COV362 parental and SR-Res models. Related to Fig. 2d-i.**

Copy number alterations are indicated as copy number ratio to ploidy. Sequence variants are classified by ACMG (American College of Medical Genetics and Genomics) guidelines.

Variant of Uncertain Significance (VUS) are indicated in light-blue; benign and likely benign in green; likely pathogenic in yellow and pathogenic variants in red.

**Abbreviations:** ***MMR***: Mismatch Repair; ***MS stable***: MicroSatellite stable (<0.0041); ***MSI-H***: MicroSatellite Instability–High (>0.0124); ***gLOH***: genomic Loss of Heterozygosity (positive ≥0.16); **HRD-positive**: Homologous Recombination Deficiency (score ≥0.7); ***TMB-high***: Tumor Mutational Burden (>10 mutations/Mb);

**Supplementary Table 5. Genes contributing to Fanconi Anemia and Base Excision Repair pathway enrichments. Related to Fig.5j-n and to Supplementary Fig. 8j-k.**

The table reports gene symbols, gene and transcript length, MANE annotation, pathway assignment, functional role, splicing events affected in each condition, and gene length category.

**OVCAR8 Parental (SR4835)**

**OVCAR8 PT-Res (SR4835)**

**OVCAR8 SR-Res (SR4835)**

**OVCAR8 SR-Res (CDDP)**

**COV362 Parental (CDDP)**

**COV362 SR-Res (CDDP)**

**Notes: *Gene length category**: <15kb: short; 15kb-100kb: mid-length; >100kb: long as defined according to Reference 109 in the main text: Brown, J. C. Role of Gene Length in Control of Human Gene Expression: Chromosome-Specific and Tissue-Specific Effects. *International Journal of Genomics* **2021**, 8902428 (2021)

**Abbreviations:** ***Core FA***: fork replication block sensor due to cross-linked DNA; ***HR***: Homologous Recombination; ***TLS***: Translesion Syntesis; ***MMR***: Mismatch Repair.
